# Chemical Characterization of Lipophilic Constituents in the Skin of Migratory Adult Sea Lamprey from the Great Lakes Region

**DOI:** 10.1371/journal.pone.0168609

**Published:** 2016-12-19

**Authors:** Amila A. Dissanayake, C. Michael Wagner, Muraleedharan G. Nair

**Affiliations:** 1 Department of Horticulture, Michigan State University, East Lansing, Michigan, United States of America; 2 Department of Fisheries and Wildlife, Michigan State University, East Lansing, Michigan, United States of America; 3 College of Food and Agriculture Sciences, Chair of Date Palm Research, King Saud University, Riyadh, Saudi Arabia; Visva-Bharati, INDIA

## Abstract

The sea lamprey (*Petromzons marinus*) is an invasive ectoparasite of large-bodied fishes that adversely affects the fishing industry and ecology of the Laurentian Great Lakes. Lipid content in the whole sea lamprey and muscles, liver and kidney of metamorphosing larval stages has been reported. Similarly, the fatty acid profile of the rope tissues of sexually-mature male sea lampreys has also been reported. The average body weight of a sub-adult migratory sea lamprey is 250 g, which includes 14.4% skin (36 g). Our preliminary extraction data of an adult sea lamprey skin revealed that it contained approximately 8.5% of lipophilic compounds. Lamprey skin is home to a naturally aversive compound (an alarm cue) that is being developed into a repellent for use in pest management. As part of an ongoing investigation to identify the chemical structure of the sea lamprey alarm cue, we extracted the skin with water and methanol, respectively. The methanolic extract (1.55%) contained exclusively lipophilic compounds and did not include the alarm cue. We chemically characterized all compounds present in the methanolic extract as cholesterol esters (CE), tri- and di-glycerides (TG and DG), cholesterol, free fatty acids (FFA) and minor amounts of plasticizers. The free fatty acids fraction was composed of saturated (41.8%), monounsaturated (40.7%) and polyunsaturated (17.4%) fatty acids, respectively. The plasticizers characterized were phthalate and benzoate and found to be 0.95 mg and 2.54 mg, respectively, per adult sea lamprey skin. This is the first report of the chemical characterization of all the lipophilic constituents in the skin of sub-adult migratory sea lamprey. The CEs isolated and characterized from sea lamprey skin are also for the first time.

## Introduction

Lampreys, along with hagfishes, represent the two extant groups of basal vertebrates. These most primitive cartilaginous fishes evolved from jawless vertebrates more than 400 million years ago and include several parasitic species [1,2]. Among these the sea lamprey (*Petromzons marinus)* has become a critically important model species for the study of behavior, developmental biology, evolutionary biology, and biomedical research [3,4]. The sea lamprey is native to the Atlantic basin, and its complex life-cycle consists of larval, parasitic juvenile and anadromous or potamodromous adult stages [5–7]. After a parasitic feeding stage that lasts 12–18 months, the sub-adult sea lamprey migrates into rivers from lakes or the coastal ocean to mature, spawn and die. At the onset of the reproductive migration the sea lamprey permanently ceases feeding and persists on residual energy stores [8]. The larvae of the next generation reside in river sediments, feeding on organic matter and algae until a radical metamorphosis into the parasitic form and out-migration to lakes or oceans [9].

In North America, the sea lamprey colonized the Laurentian Great Lakes via artificial connections (shipping canals) to the St. Lawrence seaway that allowed migrants to bypass the Niagara Falls. Because of its parasitic nature and lack of natural predators in the Great Lakes, the adverse effects on fishing industry and local ecosystems continues [10]. In particular, populations of lake trout (*Salvelinus namaycush*) and lake whitefish (*Coregonus clupeaformis*), mainstays of a recreational fishery valued at several billion dollars annually, have been severely impacted by sea lamprey when compared to population density in the 1940s and 1950s [11]. As a result, the Great Lakes Fisheries Commission (GLFC) conducts numerous research and control programs to reduce the sea lamprey population. The primary means of control is the application of selective pesticides (lampricides) to rivers to reduce larval populations before metamorphosis into the harmful parasitic life-stage [11]. At a current annual cost of ~ $20 million per year, the pesticide management program treats 20–25% of the streams that house larval lampreys each year.

Lipids, and lipid metabolism, are thought to play an important role as an energy source during metamorphosis to the parasitic form, and may function directly in the complex metamorphosis process that includes the elaboration of sensory systems as well as gut and feeding apparatus [9]. Interestingly, the migratory sea lamprey is a delicacy with high economic value in several European countries [12–14] for centuries; probably due in part to its high lipid content. More recently, lipid storage, and its role in the energetics of feeding and migration in particular, are topics of emerging interest. For example global climate change is predicted to alter the energetics of host-parasite interactions in favor of the parasite [15]. A recent bioenergetics model suggests the sea lamprey, when subject to warming regimes, will increase feeding rate, ultimately growing larger and more fecund, resulting in increased mortality among host fishes [16]. It is reasonable to anticipate the additional growth, and lipid storage, will also result in a greater migratory ability in the species, leading to greater recruitment to the larval stage. In the Great Lakes, this may result in exacerbation of an already difficult invasive species management problem. However, in its native range, the sea lamprey may enjoy greater ability to surmount energetically-costly barriers to migration (e.g. fish passage devices), leading to positive conservation outcomes [17].

There are also several reports on total fatty acid profiles from muscles from sub-adult sea lampreys during migration from river basins in Portugal [12,18,19], and a fatty acid profile of the rope tissues from sexually-mature sea lampreys from Lake Huron, Michigan, thought to play a role in mating [20]. We are working to characterize numerous compounds present in migratory sea lamprey skin, including an alarm cue thought to be important in the detection and avoidance of predation during migration [21]. To isolate the sea lamprey alarm cue molecule in its skin, we sequentially extracted the adult migratory sea lamprey skin, devoid of other tissues and blood, with water and methanol. Field trials indicated the methanolic extract was not deterrent to sea lamprey, but a preliminary analyses revealed that it contained only lipophilic compounds. No report is available addressing the chemical characterization of the lipid-soluble constituents in sea lamprey skin. Given the importance of this species as a model organism, and as a species of conservation concern, this report is focused on the determination of chemical identities of lipid-soluble constituents in the skin of sub-adult migratory sea lamprey skin that may prove important in the bioenergetics of migration and responses to climate change induced warming in its native and introduced ranges.

## Materials and Methods

### General procedures for chromatographic purification and spectroscopic analyses

ACS reagent grade solvents were purchased from Sigma–Aldrich Chemical Company (St. Louis, MO, USA) and used for all isolation and purification steps. Merck silica gel (60 mesh size, 35−70 μm) with particle size of 60 μm was used for preparative medium-pressure liquid chromatography (MPLC). Silica gel plates (250 μm; Analtech, Inc., Newark, DE, USA) were used for preparative thin-layer chromatography (TLC). After developing, TLC plates were observed under UV light at 254 and 366 nm in a Spectroline CX-20 ultraviolet fluorescence analysis cabinet (Spectroline Corp., Westbury, NY, USA) and sprayed with 10% sulfuric acid solution. NMR spectra were recorded on 500 MHz (Varian Unity ±500, ^1^H NMR) and 125 MHz (Varian Unity ±500, ^13^C NMR) VRX instruments. ESIMS spectra were recorded on a Waters Xevo G2-S Q-TOF LC mass spectrometer (Waters Corporation, Milford, MA, USA) and GCMS on Thermo DSQ-II GC/single quadrupole mass spectrometer (Thermo Electron Corporation, Austin, TX USA).

### Collection of adult migratory sea lamprey and its skin samples

We obtained 429 live, actively migrating male and female sea lampreys from the U.S. Fish and Wildlife Service (USFWS) in May and June 2015. The lampreys were captured in traps placed near dams in two tributaries to Lake Huron (the Cheboygan and Ocqueoc Rivers) and one tributary to Lake Michigan (Manistique River), in Michigan, USA. Following capture, the subjects were transported to the Hammond Bay Biological Station (Millersburg, Michigan, USA) in aerated live-wells and placed into 1000 L holding tanks that received a continuous flow of fresh Lake Huron water (100% exchange every 2 h). Prior to removal of the skin, each subject was euthanized via cervical dislocation with a scalpel. A single incision was made around the circumference of the body immediately anterior to the posterior gill opening. The skin was peeled from anterior to posterior in a single piece, cleaned of any muscle tissue, and rinsed for several minutes in distilled water. The skins were placed into plastic bags and frozen at -20°C until use in the extraction protocol. All procedures for lamprey maintenance, euthanasia, and processing were approved by the Michigan State University Institutional Animal Care and Use Committee (permit # AUF 01/14-007-00).

### Hydrolysis and methylation of triglycerides and GCMS analyses of resulting fatty acid methyl esters

An aliquot of (1 mg) sample was stirred with KOH in MeOH (3M, 3 h), acidified with HCl and evaporated under vacuum. The resulting fatty acids were then methylated with CH_2_N_2_ separately to afford fatty acid methyl esters, according to the reported procedure [22]. The methyl esters thus obtained from samples were analyzed separately by GC using a capillary column, Agilent J&W VF-5ms GC Column, 30 m x 0.25 mm, 0.25 *μ*m film thickness attached to a 10 m EZ-Guard column with a 7-inch cage. The conditions for the analyses were 1 *μ*L sample dissolved in chloroform, helium carrier gas at a flow rate of 1.5 mL/min and temperature gradient with an injector port temperature at 40°C, held for 1 min, raised to 160°C at a rate of 40°C and then raised to 210°C at a rate of 3°C followed by raising the temperature up to 250°C at a rate of 40°C. Triplicate GC analyses were preformed and the results expressed in GC area percent as mean ± SD. Chromatographic peaks were identified by comparing retention times with a standard mixture containing 37 fatty acid methyl esters (Supelco^®^ 37 Component FAME Mix, 47885-U, Bellefonte, PA, USA).

### Methylation of FFA and GCMS analyses of resulting fatty acid methyl esters

Free fatty acids were methylated with CH_2_N_2_ to afford corresponding fatty acid methyl esters, according to the reported procedure [22].

### Extraction of whole sea lamprey and skin

Frozen sea lamprey skins 1.88 kg (180 skins) were cut in to small pieces and sequentially extracted with water (2 L, 3x) and methanol (2 L, 3x). Water extract was lyophilized to yield a water-soluble powder (32 g). Evaporation of methanol under vacuum afforded an oily extract (29.2 g). Similarly, a whole sea lamprey (frozen, 292 g) was sliced and sequentially extracted with water (500 mL × 3, 5 h) and MeOH (500 mL × 3, 5 h). Water extract was lyophilized to yield a water-soluble powder (12 g). Methanolic extract was partitioned with hexane and evaporation of hexane under vacuum afforded the total lipids (21.4 g). Also, whole frozen sea lampreys (5.09 Kg) were cut to small pieces and lyophilized (1.37 Kg). An aliquot of the lyophilized whole sea lamprey (399 g) was sequentially extracted with hexane, ethyl acetate, methanol and water. Removal of solvent afforded 118.9, 4.85, 45.6 and 10.2 g of solvent-free extracts, respectively. The hexane and ethyl acetate extracts contained the total lipids.

### Chromatographic purification and isolation of pure compounds in sea lamprey skin extract

**Fig 1:**
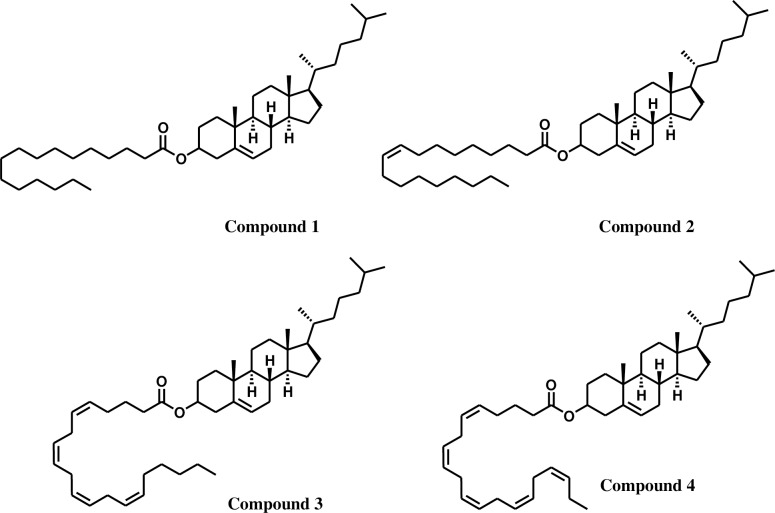
Chemical structures of cholesterol esters (CE) isolated from methanolic extract: Compound 1 (cholesteryl palmitate), compound 2 (cholesteryl oleate), compound 3 (cholesteryl arachidonate) and compound 4 (cholesteryl eicosapentaenoate).

An aliquot of the methanolic extract (4.45 g) was fractionated by medium pressure liquid chromatography (MPLC) on a silica gel column (4x250 cm glass column, silica particle size 60μ) by eluting with hexane:acetone (15:1, 4:1, v/v), acetone (100%) and methanol 100% to yield fractions **A** (113 mg), **B** (1.35 g), **C** (1.13 g) and **D** (1.76 g), respectively. An aliquot of the fraction **A** (89 mg) was further purified by preparative TLC (pentane:chloroform, 6:1, v/v, three runs) to afford compound **1** (cholesteryl palmitate [23], 10 mg, Figures A-F S1 File), compound **2** (cholesteryl oleate [24], 18 mg, Figures G-L in S1 File), compound **3** (cholesteryl arachidonate [25], 15 mg, Figures M-R in S1 File) and compound **4** (cholesteryl eicosapentaenoate [26], 14 mg, Figures S-X in S1 File), respectively (**Fig 1, Table 1**).

**Table 1:** ^1^H and ^13^C NMR spectral data of cholesterol esters (CE) 1–4^a^^,^^b^.

no.	1^a^		2^a^		3^a^		4 ^a^	
	*δ*_H_ (multi, *J* in Hz)	*δ*_C_	*δ*_H_ (multi, *J* in Hz)	*δ*_C_	*δ*_H_ (multi, *J* in Hz)	*δ*_C_	*δ*_H_ (multi, *J* in Hz)	*δ*_C_
1	1.15 (m)/1.35 (m)^c^	23.8^c^	1.14 (m)/1.37 (m)^c^	23.8^c^	1.13 (m)/1.35 (m)^c^	23.8^c^	1.13 (m)/1.34 (m)^c^	23.8^c^
2	1.56 (m)/ 1.87 (m)	27.8	1.54 (m)/ 1.85 (m)	27.8	1.52 (m)/ 1.84 (m)	27.8	1.50 (m)/ 1.86 (m)	27.8
3	4.61 (m)	73.6	4.59 (m)	73.7	4.62 (m)	73.7	4.60 (m)	73.9
4	2.26–2.31 (m, 2H)^c^	38.1	2.27–2.31 (m, 2H)^c^	38.1	2.26–2.32 (m, 2H)^c^	38.1	2.28–2.31 (m, 2H)^c^	38.1
5		139.7		139.7		139.6		139.6
6	5.34 (m)	122.6	5.31 (m)	122.5	5.31 (m)	122.6	5.33 (m)	122.6
7	1.45 (m, 2H)^c^	31.8	1.45 (m, 2H)^c^	31.8	1.44 (m, 2H)^c^	31.8	1.43 (m, 2H)^c^	31.8
8	1.48 (m)^c^	31.9	1.48 (m)^c^	31.9	1.47 (m)^c^	31.8	1.47 (m)^c^	31.8
9	0.94 (m)^c^	49.9	0.96 (m)^c^	49.9	0.95 (m)^c^	49.9	0.94 (m)^c^	49.9
10		36.5		36.6		36.6		36.6
11	1.49 (m, 2H) ^c^	21.0	1.51 (m, 2H) ^c^	21.0	1.50 (m, 2H) ^c^	21.0	1.52 (m, 2H) ^c^	21
12	1.16 (m)^c^/ 1.98 (m)^c^	39.7	1.13 (m)^c^/ 1.94 (m)^c^	39.7	1.12 (m)^c^/ 1.97 (m)^c^	39.7	1.14 (m)^c^/ 1.94 (m)^c^	39.7
13		42.3		42.3		42.3		42.3
14	1.03 (m)^c^	56.6	1.06 (m)^c^	56.7	1.05 (m)^c^	56.7	1.05 (m)^c^	56.6
15	1.15 (m)^c^ /1.58 (m)^c^	24.3	1.12 (m)^c^ /1.61 (m)^c^	24.3	1.13 (m)^c^ /1.60 (m)^c^	24.3	1.13 (m)^c^ /1.62 (m)^c^	24.2
16	1.27 (m)^c^ /1.89 (m)^c^	28.2	1.28 (m)^c^ /1.86 (m)^c^	28.2	1.26 (m)^c^ /1.84 (m)^c^	28.2	1.27 (m)^c^ /1.86 (m)^c^	28.2
17	1.08 (m)^c^	56.1	1.10 (m)^c^	56.1	1.07 (m)^c^	56.1	1.09 (m)^c^	56.1
18	0.67 (s)	11.8	0.65 (s)	11.8	0.65 (s)	11.8	0.65 (s)	11.8
19	1.02 (s)	19.3	0.99 (s)	19.3	0.99 (s)	19.3	0.98 (s)	19.3
20	1.41 (m)	35.7	1.40 (m)	35.8	1.41 (m)	35.8	1.40 (m)	35.8
21	0.91 (d, 6.4)	18.7	0.88 (d, 6.4)	18.7	0.89 (d, 6.4)	18.7	0.88 (d, 6.4)	18.7
22	1.15 (m)^c^/1.30 (m)^c^	36.2	1.17 (m)^c^/1.33 (m)^c^	36.2	1.16 (m)^c^/1.33 (m)^c^	36.1	1.15 (m)^c^/1.32 (m)^c^	36.1
23	1.22 (m, 2H) ^c^	23.8^c^	1.21 (m, 2H) ^c^	23.8^c^	1.21 (m, 2H) ^c^	23.8^c^	1.20 (m, 2H) ^c^	23.8^c^
24	1.25 (m, 2H) ^c^	36.9	1.27 (m, 2H) ^c^	36.9	1.27 (m, 2H) ^c^	36.9	1.25 (m, 2H) ^c^	36.9
25	1.52 (m)	28.0	1.50 (m)	28.0	1.51 (m)	28.0	1.49 (m)	28
26	0.87 (d,2.5)	22.8	0.86 (d,2.5)	22.7	0.88 (d,2.5)	22.8	0.87 (d,2.5)	22.8
27	0.85 (d, 1.9)	22.5	0.83 (d, 1.9)	22.6	0.85 (d, 1.9)	22.6	0.87 (d, 1.9)	22.6
1′		173.3		173.3		173.0		173.0
2′	2.26 (dd. 2H, 7.4)	34.7	2.22 (dd. 2H, 7.4)	34.7	2.21 (dd. 2H, 7.4)	34.0	2.25 (dd. 2H, 7.4)	34.0
3′	1.25–1.39 (m, 26H)^c^	25.1	1.25–1.39 (m, 10H)^c^	31.9	1.82 (m)	31.9	1.82 (m)	31.9
4′		29.1		29.7	2.24 (m, 2H) ^c^	36.2	2.26 (m, 2H) ^c^	36.1
5′		29.2		29.6	5.28–5.42 (m, 2H) ^c^	129.0–127.5	5.24–5.40 (m, 2H) ^c^	129.1–126.9
6′		29.3		29.5				
7′		29.3 29.4 29.4 29.5 29.6 29.6 29.7 29.7 29.8		29.7	2.77–2.81 (m, 2H) ^c^	34.0	2.78–2.82 (m, 2H) ^c^	34.0
8′			1.92–2.05 (m, 2H) ^c^	31.8	5.28–5.42 (m, 2H) ^c^	129.0–127.5	5.24–5.40 (m, 2H) ^c^	129.1–126.9
9′			5.36–5.32 (m, 2H)	129.7				
10′				129.9	2.77–2.81 (m, 2H) ^c^	35.7	2.78–2.82 (m, 2H) ^c^	35.7
11′			1.92–2.05 (m, 2H) ^c^	31.8	5.28–5.42 (m, 2H) ^c^	129.0–127.5	5.24–5.40 (m, 2H) ^c^	129.1–126.9
12′			1.25–1.39 (m, 12H)^c^	29.7				
13′				29.3	2.77–2.81 (m, 2H) ^c^	36.9	2.78–2.82 (m, 2H) ^c^	36.9
14′				29.1	5.28–5.42 (m, 2H) ^c^	129.0–127.5	5.24–5.40 (m, 2H) ^c^	129.1–126.9
15′		22.7		27.1				
16′	0.87 (m)	14.1		25.0	2.77–2.81 (m, 2H) ^c^	36.6	2.78–2.82 (m, 2H) ^c^	36.6
17′				22.8	1.25–1.36 (m, 6H)^c^	25.6	5.24–5.40 (m, 2H) ^c^	129.1–126.9
18′			0.87 (m)	14.1		24.8		
19′						22.6	1.96 (m, 2H) ^c^	39.4
20′					0.85 (m)	14.1	0.93 (m)	14.3

^a^ Data were measured in CDCl_3_.

^b^ Compound **1** Figures A-F in S1 File, compound **2** Figures G-L in S1 File, compound **3,** Figures M-R in S1 File, compound **4** Figures S-X in S1 File.

^c^ Overlapped signals.

**Fig 2:**
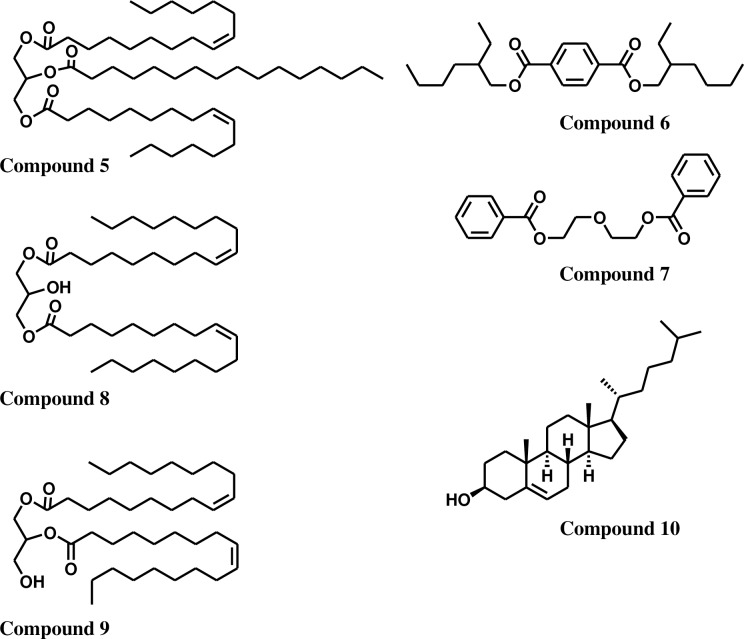
Chemical structures of isolates from methanolic extract: Compund 5 ((1,3-Di(cis-9-hexadecenoyl)-2-hexadecanoyl-glycerol), compound 6 (bis-(2-ethylhexyl) terephthalate), compound 7 (diethylene glycol dibenzoate), compound 8 (1,3-di(cis-9-octadecenoyl)glycerol), compound 9 (1,2-di(cis-9-octadecenoyl)glycerol), compound 10 (cholesterol).

Fraction **B** (125 mg) was fractionated by silica gel MPLC by eluting with pentane:acetone (20:1 and 4:1, v/v) to yield two fractions **E** (58 mg) and **F** (55 mg). Fraction **E** was identified as compound **5** (1,3-Di(cis-9-hexadecenoyl)-2-hexadecanoyl-glycerol [27,28], Figures A-F in S2 File) (**Fig 2**). Components in fraction **F** was in separable and identified by ^1^H NMR spectral data as a mixture of triglycerides. Characterization of fatty acid composition and identity of triglycerides in fraction **F** was achieved by GCMS analyses of the methylated product from its hydrolysis. Data revealed that it was esters of myristic (C14:0), palmitic (C16:0), palmitoleic (C16:1), oleic (C18:1), arachidonic (C20:4), eicosapentaenoic (C20:5), and docosahexaenoic acids (C22:6) with glycerol (Figures G-H in S2 File).

Similarly, fraction **C** (388 mg) was fractionated on silica MPLC by gradient elution with chloroform:methanol (50:1, 4:1, v/v,) to yield fractions **G** (123 mg) and **H** (237 mg), respectively. An aliquot of the fraction **G** (123 mg) was further purified by preparative TLC (hexane:isopropanol, 25:1, v/v, two runs) to afford three fractions **I** (10 mg), **J** (25 mg) and **K** (53 mg), respectively. An aliquot of fraction **I** was further purified by preparative TLC (CHCl_3_-MeOH, 200:1 v/v) to afford compound **6** (9 mg), bis-(2-ethylhexyl) terephthalate (Figures I-L in S2 File) [29]. Similarly, purification of fraction **J** (24 mg) yielded compound **7** (18 mg), diethylene glycol dibenzoate (**Fig 2**) (Figures M-Q in S2 File) [30]. An aliquot of the fraction **K** (40 mg) was further purified by preparative TLC (hexane:acetone:acetic acid, 4:1:0.01, v/v, four runs) to afford compounds **8** (15 mg) and **9** (14 mg) and identified as 1,3-di(cis-9-octadecenoyl)glycerol (Figures R-S in S2 File) and 1,2-di(cis-9-octadecenoyl)glycerol (Figures T-U in S2 File) respectively (**Fig 2**) [27,28], respectively. An aliquot of fraction **H** (20 mg) was further purified by preparative TLC (CHCl_3_-MeOH, 50:1 v/v, two runs) to afford compound **10** (16 mg) and identified as cholesterol (Figure V in S2 File) (**Fig 2**) [31].

Lipid components in the hexane extract of the whole sea lamprey were identified by ^1^H NMR spectral data and showed it as a mixture of lipids. It contained mixtures of glycerides and FFAs that were identical to the lipid components characterized in the methanolic extract of sea lamprey skin (Figures A-B in S3 File). Characterization of fatty acid composition was achieved by GCMS analyses of the methylated products from its hydrolysis [22]. GCMS Data revealed that fatty acid profiles were identical to the lipids obtained from whole sea lamprey and its skin separately. (Figure C in S3 File).

## Results

The chemical identity of all pure compounds isolated from sea lamprey skin were determined by ^1^H- and ^13^C-NMR and MS analyses. Identity of FFA was achieved by its methylation using diazomethane to yield methyl esters followed by GC-MS analyses. Compound **1**, a colorless oil, possessed the molecular formula of C_43_H_76_O_2_, as determined by HRESIMS ions at *m*/*z* 647.5790 ([M + Na]^+^(calcd for C_45_H_78_O_2_Na, 647.5743) (**Fig 1**) (Figure E in S1 File). The NMR data of compound **1** revealed that it contained 6 methyl, 25 methylene and 8 methine groups along with 4 quaternary carbons (**Table 1**). Compound **1** displayed characteristic oxygenated methine group (C-3 *δ*_C_ 73.6 *δ*_H_ 4.61), unsaturated methine group (C-6 *δ*_C_ 122.6 *δ*_H_ 5.34) and 6 tertiary methyls (*δ*_H_ 1.02, 0.91, 0.87, 0.85, and 0.67) of a cholesteryl moiety [31]. In addition, the NMR spectra of compound **1** showed proton and carbon signals with chemical shifts that were characteristic of long chain fatty acid esters. The fatty acid ester was assigned at C-3 of the cholesterol moiety on the basis of HMBC correlations observed between C3 (*δ*_C_ 73.6) to C1′ (*δ*_C_ 173.3) (Figure D in S1 File). Compound **1** was hydrolyzed and methylation of the resulting product afforded the corresponding fatty acid methyl ester [22]. The molecular ions at *m*/*z* 270 (methyl palmitate) in its HRESIMS further confirmed the identity of the fatty acid moiety in **1** and its proposed structure (**Fig 1**) [23].

Similarly, compounds **2**, a colorless oil, showed the molecular formula of C_45_H_78_O_2_, as determined by HRESIMS ion at *m*/*z* 673.5901 ([M + Na]^+^(calcd for C_45_H_78_O_2_Na, 673.5899) with the characteristic cholesteryl moiety in the structure (**Table 1, Fig 1**) (Figure K in S1 File). In addition to signals for a cholesteryl moiety, the NMR spectra of compound **2** showed proton and carbon signals with chemical shifts that were characteristic of long chain fatty acid esters with one unsaturation (*δ*_C_ 129.7, 129.8 and *δ*H 5.32–5.36) (Figures G-J in S1 File). The fatty acid ester was assigned at C-3 of the cholesterol moiety on the basis of HMBC correlations observed between C3 (*δ*_C_ 73.7) to C1′ (*δ*_C_ 173.3) (Figure J in S1 File). Compound **2** was hydrolyzed and methylation of the resulting product afforded the corresponding fatty acid methyl ester [22]. GCMS and HRESIMS analyses of the resulting fatty acid ester gave molecular ions at *m*/*z* 270 (methyl oleate) and further confirmed the proposed structure of **2** (**Fig 1**) [24].

As in the case of compounds **1** and **2**, compound **3** was also a colorless oil. It showed the molecular formula of C_45_H_78_O_2_, as confirmed by HRESIMS ion at *m*/*z* 695.5746 ([M + Na]^+^ (calcd for C_47_H_76_O_2_Na, 695.5743), and a cholesteryl moiety in its structure (**Table 1, Fig 1**) (Figure Q in S1 File). In addition to signals for a cholesterol moiety, NMR spectra of compound **3** showed proton and carbon signals with chemical shifts that were characteristic of long chain fatty acid esters with four degrees of unsaturation (*δ*_C_ 127.5–129 and *δ*H 5.28–5.42) (Figures M-O in S1 File). The fatty acid ester moiety was assigned at C-3 of the cholesterol moiety on the basis of HMBC correlations observed between C3 (*δ*_C_ 73.7) to C1′ (*δ*_C_ 173) (Figure P in S1 File). Compound **3** was hydrolyzed and methylation of the resulting product afforded the corresponding fatty acid methyl esters [22]. GCMS and HRESIMS analyses of the resulting fatty acid ester gave molecular ions at *m*/*z* 318 (methyl arachidonate) and further confirmed the proposed structure of **3** (**Fig 1**) [25].

Compound **4** was also a colorless oil and showed the molecular formula of C_45_H_78_O_2_, as confirmed by the molecular ion at *m*/*z* 693.5581 ([M + Na]^+^(calcd for C_47_H_74_O_2_Na, 693.5586) in its HRESIMS. It also indicated the characteristic cholesteryl moiety in the structure (**Table 1, Fig 1**) (Figure W in S1 File). In addition to signals for a cholesterol moiety, NMR spectra of compound **4** showed proton and carbon signals with chemical shifts that were characteristic of long chain fatty acid ester with five degrees of unsaturation (*δ*_C_ 126.9–129.1 and *δ*H 5.24–5.40) (Figures S-U in S1 File). This fatty acid ester moiety was assigned at C-3 of the cholesterol moiety on the basis of HMBC correlations observed between C3 (*δ*_C_ 73.9) to C1′ (*δ*_C_ 173) (Figure V in S1 File). Compound **4** was hydrolyzed and methylation of the resulting product afforded the corresponding fatty acid methyl esters [22]. GCMS and HRESIMS analyses of the resulting fatty acid ester gave a molecular ion at *m*/*z* 316 (methyl eicosapentaenoate) and further confirmed proposed structure of **4** (**Fig 1**) [26].

Compound **5**, was isolated as colorless oil and the NMR data of compound **5** displayed characteristic oxygenated methine group (C-2 *δ*_C_ 68.8, H-2 *δ*_H_ 5.21−5.26) and two oxygenated methylene group (C-1 and 3 *δ*_C_ 62.1 *δ*_H_ 4.25–4.28 (dd, *J* = 11.7, 4.4 Hz) and 4.10–4.13 (*J* = 11.8, 5.9 Hz), a symmetrical tri-substituted glycerol moiety. In addition, the NMR spectra of compound **5** showed proton and carbon signals with chemical shifts that were characteristic of long chain fatty acid esters (Figures A-B in S2 File). The fatty acid esters were assigned at C-1 and C-3 of the triglyceride moiety on the basis of HMBC correlations observed between C-1 and C-3 (*δ*_C_ 62.1) to C-1′ and C-1′′′ (*δ*_C_ 173.3). Similarly, C-2 of the triglyceride moiety was assigned on the basis of HMBC correlations observed between C-2 (*δ*_C_ 68.8) to C-1′′ (*δ*_C_ 173.2) (Figure C in S2 File). Furthermore, GCMS analysis of fatty acid esters resulting from its hydrolysis and methylation gave molecular ions at *m*/*z* 270 and 268. This confirmed methyl palmitate and methyl palmitoleate moieties at 2:1 ratio and further helped to confirm the structure of compound **5** as 1,3-Di(cis-9-hexadecenoyl)-2-hexadecanoyl-glycerol [27,28] (**Fig 2**). Also, hydrolysis of fraction **F** containing the triglyceride mixture gave peaks at R_t_ = 6.57, 8.44, 8.81, 11.43, 16.45, 17.61, and 21.34 min with *m/z* values at 242, 270, 268, 296, 318, 316, and 342, respectively, for methyl esters of myristic, palmitic, palmitoleic, arachidonic, eicosapentaenoic and docosahexaenoic acids (Figure H in S2 File).

Compound **6**, isolated as a colorless oil, showed the molecular formula of C_24_H_38_O_4_, as determined by its molecular ion at *m*/*z* 391 [M+H] (Figure L in S2 File)_._ The ^1^H-NMR spectrum provided the evidence for the substitution at 1 and 4 positions of the aromatic moiety (*δ*_H_ 8.11, singlet) (Figure I in S2 File). Five additional methylene (*δ*_C_ 67.7, 30.5, 28.9, 23.9, and 22.9), one methine (*δ*_C_ 38.9) and two methyl groups (*δ*_C_ 14.0, and 11.0) were also observed that exhibited chemical shifts values that coincided with those for a 2-ethylhexyl moiety (Figures J-K in S2 File). Therefore, based on the spectral data, compound **6** was identified as bis-(2-ethylhexyl) terephthalate [29]. Compound **7**, isolated as colorless oil, showed an AMX spin system with proton chemical shifts at *δ*_H_ 8.02 (dd, 8.3, 1 Hz), 7.72 (dd, 7.7, 1.5 Hz), and 7.36 (dd, 7.9, 2 Hz) in its H-NMR spectrum. This indicated that it contained a mono-substituted aromatic ring. The carbonyl group in the molecule was assigned to a benzoate moiety (C-7, *δ*_C_ 166.5) on the basis of HMBC correlations observed between C7 (*δ*_C_ 166.5) to C1 (*δ*_C_ 129.9) (Figures M-P in S2 File). In addition, oxygenated methylenes (*δ*_C_ 69.2 and 63.9, *δ*_H_ 4.48 (dd, 5.9, 3.4 Hz) and 3.87 (dd, 5.9, 3.5 Hz)) belonging to diethylene glycol moiety were linked to the carbonyl (*δ*_C_ 166.5) of the benzoate at C-1, as indicated by HMBC correlations between C7 (*δ*_C_ 166.5) and C8 (*δ*_C_ 63.9) (Figure P in S2 File). According to the spectral data, compound **7** was identified as diethylene glycol dibenzoate [30] (**Fig 2**).

Compound **8** was identified as 1,3 di-substituted glycerol based on the characteristic signals observed for a tertiary hydroxyl group (C-2 *δ*_C_ 68.4, *δ*_H_ 4.09) in its H-NMR spectrum [27,28]. The chemical shift of the carbonyl group in its ^13^C-NMR at *δ*_C_ 173.9 revealed that the glycerol moiety was substituted with identical fatty acids at 1 and 3 (Figures R-S in S2 File). Hydrolyses and methylation [22] of the resulting products from compound **8** afforded a single fatty acid methyl ester. The GCMS analysis of this fatty acid ester gave molecular ion at *m*/*z* 296 and confirmed it as methyl oleate and further confirmed the identity of compound **8**. As in the case of compound **8**, based on NMR and GCMS analyses, compound **9** was identified as 1,2 di-substituted glycerol. Its NMR spectra showed C3-position of the glycerol moiety at *δ*_H_ 3.72 and *δ*_C_ 61.5 and carbonyl group at C-1′ (*δ*_C_ 173.4). This conformed that the glycerol was substituted with identical fatty acids at 1 and 2 [27,28] (Figures T-U in S2 File). Hydrolyses and methylation of compound **9** afforded only one fatty acid methyl ester [22]. GCMS analysis of this fatty acid ester (R_t_ = 11.56 min) gave a molecular ion at *m*/*z* 296 and confirmed it as methyl oleate. Compound **10** was isolated as a white powder and identified as cholesterol [31] (Figure V in S2 File) (**Fig 2**).

### Characterization of free fatty acids (FFA) in sea lamprey skin

**Fig 3:**
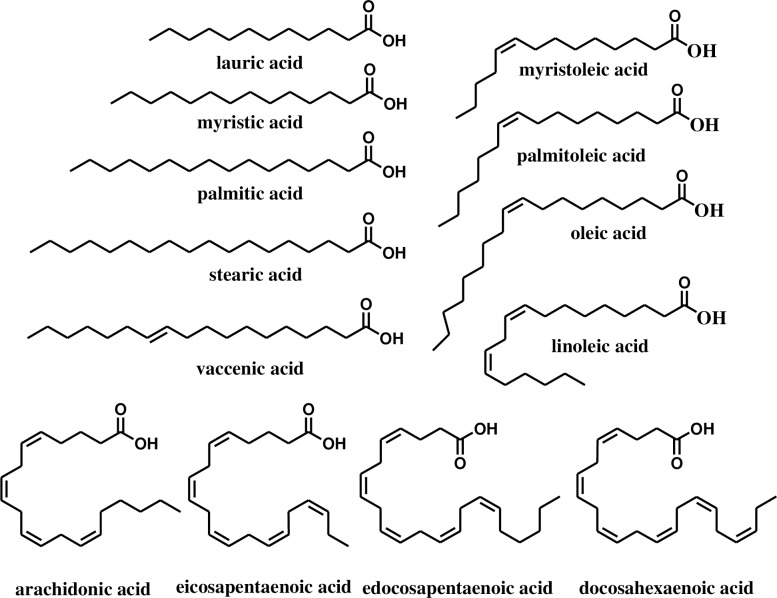
Chemical structures of free fatty acids (FFA) characterized in sea lamprey skin.

Components of the fraction **D** were identified by the ^1^H NMR spectral data as a mixture of FFAs (Figure D in S3 File). The chemical characterization of the FFA was carried out by GCMS analyses. The observed peaks in the GC profile at R_t_ = 5.35, 6.57, 6.90, 8.44, 8.81, 11.06, 11.43, 11.55, 12.26, 16.45, 17.61, 21.21 and 21.34 min gave molecular ions at *m/z* values of 214, 242, 240, 270, 268, 284, 312, 296, 294, 318, 316, 344, and 342, respectively, for methyl esters of lauric (C12:0), myristic (C14:0), myristoleic (C14:1), palmitic (C16:0), palmitoleic (C16:1), stearic (C18:0), oleic (C18:1, (Z)), vaccenic (C18:1, (E)), linoleic (C18:2), arachidonic (C20:4), eicosapentaenoic (C20:5), docosapentaenoic (C22:5), and docosahexaenoic (C22:6) acids (**Fig 3**). The relative abundance of these fatty acids in fraction **D** was calculated from the GC profile (**Table 2**) (Figures E-N in S3 File).

**Table 2:** Relative abundance of free fatty acids in sea lamprey skin.

Fatty acid^a^	Relative abundancy %^b^
C12:0	9.01 ± 0.09
C14:0	14.38 ± 0.17
C14:1 (Z)	0.753 ± 0.003
C16:0	15.25 ± 0.11
C16:1 (Z)	24.58 ± 0.28
C18:0	3.09 ± 0.03
C18:1 (Z)	12.74 ± 0.13
C18:1 (E)	2.62 ± 0.02
C18:2 (all-Z)	0.71 ± 0.003
C20:4 (all-Z)	2.77 ± 0.02
C20:5 (all-Z)	1.44 ± 0.006
C22:5 (all-Z)	1.43 ± 0.002
C22:6, (all-Z)	11.12 ± 0.09

^a^Fatty acid identity; lauric acid (C12:0, R_t_ = 5.35 min), myristic (C14:0, R_t_ = 6.57 min), myristoleic (C14:1 (Z), R_t_ = 6.90 min), palmitic (C16:0, R_t_ = 8.44 min), palmitoleic (C16:1 (Z), R_t_ = 8.81 min), stearic (C18:0, R_t_ = 11.06 min), oleic (C18:1, (Z), R_t_ = 11.43 min), vaccenic (C18:1 (E), R_t_ = 11.55 min), linoleic (C18:2, (all-Z), R_t_ = 12.26 min), arachidonic (C20:4, (all-Z), R_t_ = 16.45 min), eicosapentaenoic (C20:5, (all-Z), R_t_ = 17.61 min), docosapentaenoic (C22:5, (all-Z), R_t_ = 21.21 min), and docosahexaenoic acids (C22:6, (all-Z), R_t_ = 21.34 min) (Figure E in S3 File).

^b^Data represent means ± SD of three replicates.

#### Spectroscopic characterization of compounds 5–9

Compound **5**: Colorless oil; (1,3-Di(cis-9-hexadecenoyl)-2-hexadecanoyl-glycerol) [27,28]. ^1^H NMR (500 MHz, CDCl_3_): *δ* 5.29−5.41 (4H, m, H-9′, 9′′′, 10′, 10′′′), 5.21−5.26 (1H, m, H-2), 4.25–4.28 (2H, dd, *J* = 11.7, 4.4 Hz, H-1a, 3a), 4.10–4.13 (2H, dd, *J* = 11.8, 5.9 Hz, H-1b, 3b), 2.30 (6H, m, H-2′, 2′′,2′′′), 1.99 (8H, m, H-8′, 8′′′, 11′, 11′′′), 1.59 (6H, m, H-3′, 3′′,3′′′), 1.23−1.39 (56H, m, H-4′-7′, H-12′-17′, H-4′′-17′′, H-4′′′-7′′′, H-12′′′-17′′′), 0.87 (9H, m, H-18′, 18′′,18′′′); ^13^C NMR (125 MHz, CDCl_3_): *δ* 173.3 (C-1′,C-1′′′), 173.2 (C-1′′) 130 (C-9′,C-9′′′), 129.6 (C-10′,C-10′′′), 68.8 (C-2), 62.1 (C-1, 3), 34.1 (C-2′,C-2′′′), 34.0 (C-2′′), 31.9 (C-2′,C-2′′′), 31.7 (C-2′′), 29.7−29.0 (C-3′′ to C-14′′, C-4′ to C-7′, C-12′ to C-14′, C-4′′′ to C-7′′′, C-12′′′ to C-14′′′), 27.2 (C-8′, C-8′′′), 27.1 (C-10′,C-10′′′), 24.8 (C-3′, C-3′′, C-3′′′), 22.7 (C-15′,C-15′′′), 22.6 (C-15′′), 14.1 (C-16′, C-16′′,C-16′′′) (Figures A-F in S2 File).

Compound **6**: Colorless oil; bis-(2-ethylhexyl) terephthalate [29]; *m*/z 391 [M+H]^+^; ^1^H NMR (500 MHz, CDCl_3_): *δ* 8.11 (4H, s, H-3, 4, 6, 7), 4.28 (4H, m, H_2_-1′, 1′′), 1.75 (2H, m, H-2′, 2′′), 1.30−1.50 (16H, m, H_2_-3′, 3′′, 4′, 4′′, 5′, 5′′, 7′, 7′′), 0.96 (6H, t, *J* = 7.5 Hz, H_3_-8′, 8′′), 0.92 (6H, t, *J* = 7.1 Hz, H_3_-6′, 6′′); ^13^C NMR (125 MHz, CDCl_3_): *δ* 165.9 (C-1, 8), 134.2 (C-2, 5), 129.4 (C-3, 4, 6, 7), 67.7 (C-1′, 1′′), 38.9 (C-2′, 2′′), 30.5 (C-3′, 3′′), 28.9 (C-4′, 4′′), 23.9 (C-7′, 7′′), 22.9 (C-5′, 5′′), 14.0 (C-6′, 6′′), 11.0 (C-8′, 8′′) (Figures I-M in S2 File).

Compound **7**: Colorless oil; diethylene glycol dibenzoate [30]; ^1^H (500 MHz, CDCl_3_): *δ* 8.02 (2H, dd, 8.3, 1 Hz, H-2,6), 7.52 (1H, dd, 7.7, 1.5 Hz, H-4), 7.36 (2H, dd, 7.9, 2 Hz, H-3,5), 4.48 (2H, dd, 5.9, 3.4 Hz, H_2_-8), 3.87 (2H, dd, 5.9, 3.5 Hz, H_2_-9); ^13^C NMR (125 MHz, CDCl_3_): *δ* 166.5 (C-7), 132.9 (C-4), 129.9 (C-1), 129.6 (C-2,6), 128.3 (C-3,5), 69.2 (C-9), 63.9 (C-8). MS EI *m/z* 77, 105, and 149 (Figures N-Q in S2 File).

Compound **8**: Colorless oil; 1,3-di(cis-9-octadecenoyl)glycerol [27,28]; ^1^H (500 MHz, CDCl_3_): *δ* 5.39−5.32 (4H, m, H-9′, 9′′, 10′, 10′′), 4.18 (2H, dd, *J* = 11.4, 4.6 Hz, H-1a, 3a), 4.14 (2H, dd, *J* = 11.6, 5.6 Hz, H-1b, 3b), 4.09 (1H, m, H-2), 2.32 (4H, m, H-2′, 2′′), 2.01 (8H, m, H-8′, 8′′, 11′, 11′′), 1.66 (4H, m, H-3′, 3′′), 1.44−1.28 (40H, m, H-4′-7′, H-12′-17′, H-4′′-7′′, H-12′′-17′′), 0.85 (6H, m, H-18′, 18′′); ^13^C NMR (125 MHz, CDCl_3_): *δ* 173.9 (C-1′, 1′′) 130.0 (C-9′, 9′′), 129.7 (C-10′, 10′′), 68.4 (C-2), 65.0 (C-1, 3), 34.1 (C-2′, 2′′), 31.8, (C-8′, 8′′), 31.7, (C-11′, 11′′), 29.7−28.9 (C-4′-7′, C-12′-16′, C-4′′-7′′, C-12′′-16′′), 24.5 (C-3′, 3′′), 22.6 (C-17′, 17′′), 14.1 (C-18′, 18′′) (Figures R-S in S2 File).

Compound **9**: Colorless oil; 1,2-di(cis-9-octadecenoyl)glycerol [27,28]; ^1^H (500 MHz, CDCl_3_): *δ* 5.38−5.32 (4H, m, H-9′, 9′′, 10′, 10′′), 4.16 (1H, dd, *J* = 11.4, 4.6 Hz, H-1a), 4.12 (1H, dd, *J* = 11.6, 5.6 Hz, H-1b), 4.06 (1H, m, H-2), 3.72 (2H, m, H-3), 2.36 (4H, m, H-2′, 2′′), 2.01 (8H, m, H-8′, 8′′, 11′, 11′′), 1.62 (4H, m, H-3′, 3′′), 1.48−1.29 (40H, m, H-4′-7′, H-12′-17′, H-4′′-7′′, H-12′′-17′′), 0.89 (6H, m, H-18′, 18′′); ^13^C NMR (125 MHz, CDCl_3_): *δ* 173.4 (C-1′, 1′′) 130.0 (C-9′, 9′′), 129.7 (C-10′, 10′′), 65.0 (C-2), 61.9 (C-1), 61.5 (C-3), 34.2 (C-2′, 2′′), 31.9, (C-8′, 8′′), 31.8, (C-11′, 11′′), 29.7−28.9 (C-4′-7′, C-12′-16′, C-4′′-7′′, C-12′′-16′′), 24.8 (C-3′, 3′′), 22.6 (C-17′, 17′′), 14.1 (C-18′, 18′′) (Figures T-U in S2 File).

## Discussion

Liver and intestinal tissues from metamorphosing larvae of sea lamprey in the Great Lakes region have been reported to contain cholesterol ester and lipids, identified by GC analysis of the tissue extract [32]. In this study, we report the isolation of pure cholesterol esters from adult sea lamprey skin extract and its characterization by NMR and GCMS methods (Table 1). The acid moiety of the four cholesterol esters (CE) characterized in the extract from adult sea lamprey skin were palmitic (C16:0), oleic (C18:1), arachidonic (C20:4) and eicosapentaenoic (C20:5) acids. These CEs contributed to 2.53% of the total lipophilic constituents of adult migratory sea lamprey skin. Kaoa et al, reported an increase in monounsaturated CEs while decreasing the saturated CEs in liver and intestine during the larval metamorphosis of sea lamprey [32]. Our results on adult sea lamprey skin showed high amounts of monounsaturated cholesterol esters relative to saturated CEs, which is in support of the turnover of saturated CEs in larvae during metamorphosis to adult. In addition, our results also confirmed significantly high amounts polyunsaturated cholesterol esters (ω-6 C20:4, and ω-3 C20:5) compared to metamorphosing larvae, where trace amounts or absence of polyunsaturated cholesterols reported from liver and intestine [32].

During metamorphosis, larvae of sea lamprey begin to store significant amounts of triglycerides in kidney, liver, fat column, subcutaneous tissues and myosepta as energy reserves for migration, which requires maximum energy input [33,34]. Our lipid analysis of sea lamprey skin showed 30.3% of triglycerides in the total lipophilic compounds isolated. Among this total triglyceride mixture, 1,3-di(cis-9-hexadecenoyl)-2-hexadecanoyl-glycerol (compound **5**) was found to be the major component (Fig 2). Apart from the major triglyceride, compound **5**, other triglycerides characterized from the skin extract were glycerol esters of SFAs (C14:0 and C16:0), MUFAs (C16:1 and C18:1), and polyunsaturated fatty acids (PUFAs) (ω-6 C20:4, ω-3 C20:5, and ω-3 C22:6) (Fig 2, supplemental data for spectroscopic and chromatographic data). Significant amounts of free cholesterol and diglycerides of C18:1 ω-9 acids, 20.1 mg and 5.6 mg per animal respectively, have also been isolated and characterized along with TGs from the migratory sea lamprey skin (Fig 2). Hydrolysis of CEs and TGs could result in the production of these compounds.

Like teleost fishes, triglyceride composition in sea lamprey depends on the lipid metabolism, nutritional status, environmental (thermal), and physiological (hormonal) factors [35]. Changes in triglyceride composition have been reported on several parasitic sea lamprey species such as *Mordacia mordax* [36], *Geotria australis* [37] and landlocked parasitic sea lamprey *Petromzons marinus* [38]. Therefore, composition of the triglycerides in sea lamprey depends on the migratory state and the specific organ of the animal. Nevertheless, the adult migratory sea lamprey skin contain high amounts of triglycerides of PUFAs (ω-6 C20:4, ω-3 C20:5, and ω-3 C22:6), which are essential nutrients. As reported in the case of larvae [33,34], these triglycerides of saturated fatty acids (SFAs) and monounsaturated fatty acids (MUFAs) may serve as essential energy pool via lipid metabolism during the migration for the adult sea lamprey.

Two synthetic compounds that are lipid soluble and isolated along with TGs from sea lamprey skin were a phthalate and a benzoate. Recently, parabene derivatives, widely used as consumer product preservatives and pharmaceuticals [39,40], have been reported to accumulate in fish, birds and bears in United States [41]. The phthalate, bis-(2-ethylhexyl) terephthalate (compound **6**) [29] and benzoate, diethylene glycol dibenzoate (compound **7**) [30] isolated from the adult migratory sea lamprey skin (Fig 2), 0.95 mg and 2.54 mg per animal respectively, are common plasticizers. Phthalate has been rated as priority pollutant by USA Environmental Protection Agency [42,43]. Its use has been restricted in European Union as well. Benzoate plasticizers are introduced as an alternative to phthalate plasticizers due their low toxicity and high rate of biodegradations [43,44]. However, recent reports indicate that diethylene glycol dibenzoates (such as compound **7**) metabolizes to monobenzoates, which exhibit higher toxicity than dibenzoates [45–48] and accumulate in tissues. Bioaccumulation of these plasticizers in sea lamprey could be attributed to its infaunal larval stage, or the parasitic feeding stage where it targets larger, older individuals that are enriched in environmental pollutants [49]. Nevertheless, accumulation of these plasticizers in the sea lamprey skin provides good indication of the pollution levels in the Great Lakes and surrounding regions. Importantly, these pollutants will be deposited back into rivers at the conclusion of the reproductive migration, shunting the chemicals from the top of the lake food web to the base of the river food web.

The free fatty acid fraction (FFA), Fraction **D**, was 39.5% of the total lipid-soluble extract isolated from the sea lamprey skin. The composition of the FFA mixture was determined by GC analyses of the methyl esters of FFAs as per reported methods from our laboratory [22]. Free fatty acids have been implicated as a nutritional marker for migrating adult sea lamprey [19]. Our result on FFA in adult sea lamprey skin showed high proportions of C16:1 (ω-9) (24%). Other major FFAs characterized were C16:0, 14:0, and C18:1 at 15.2%, 14.3% and 12.7%, respectively, in the total FFA (TFA) mixture (**Table 2**). In addition, fatty acids C12:0, C18:1 (Z), C18:2 and PUFAs (ω-6) C20:4 (ω-6), C22:5 (ω-6), C20:5 (ω-3) and C22:6 (ω-3) were also characterized by GCMS in the TFA mixture from sea lamprey skin. The amount of these fatty acids comprised of SFAs, MUFAs and PUSAs were 41.8%, 40.7%, and 17.4%, respectively, in the TFA mixture. Previous research on extracts of whole sea lamprey or tissues (muscles, liver and intestines) showed little or no C12:0 fatty acid present in the FFA profiles [18–20,32]. Our findings showed a high content of C12:0 fatty acid (9%) in the TFA mixture from the adult migratory sea lamprey skin (**Table 2**). Very good antibacterial activity [50] has been reported for FFAs. Among these FFAs, C12:0 (lauric acid) showed good anti-algal [51], antibacterial [52,53], anti-fungal [54] and antiviral [55] activities. Also recent studies have demonstrated that ω-3 PUFAs influences cutaneous wound healing process [56]. Therefore, accumulation of large amount of C12:0 and ω-3 FFA in sea lamprey skin could act as a defense to combat secondary infection of wounds acquired during migration [57]. Although fish alarm cues are thought to have evolved from anti-microbial compounds [57], subsequent behavioral assays with the methanolic extracts (C.M. Wagner, unpub. data) showed no repellent response.

The FAs in glycerides and FFAs in the total lipid extract of the whole sea lampreys were identical to FAs and FFAs characterized from the sea lamprey skin. Also, our findings revealed that frozen and lyophilized adult sea lamprey afforded 7.34% and 8.55% of total lipids, respectively. The skin from the frozen sea lamprey gave 1.55% of total lipids. The difference in total lipid yields from fresh and lyophilized sea lamprey was due to the extraction protocols. That is, lipids can be extracted from the lyophilized sample much better than from a fresh or frozen sample. Also, total lipid content in adult sea lampreys could vary and depends on the maturity of the animals, nutritional status, and environmental factors [35].

The fatty acid composition in fish oil from common fish species with high economical values such as mackerel (*Rastrilliger kanagurta)*, [58] salmon (*Salmo salar L*), [59] sardines (*Sardinella Brasiliensis*), [60] Atlantic cod (*Gadus morhua L*) [61] and anchovy (*Engraulis encrasicolus* L) [62] have been reported. Although data on total lipids from whole fish is not available, salmon (*Salmo salar L*) [59] fillet reportedly contain about 7% of total lipids to its body weight. The relative amount of fatty acids in the fish oil from these fishes was comprised of SFAs, MUFAs and PUSAs at 23.2%, 45.6% and 26.2%, respectively, and identical to the lipids characterized in sea lamprey. MUFAs content reported in salmon fillet was similar to our finding of MUFAs in sea lamprey. The FA composition in fish was depended on the FA content of the feed and environmental factors. Studies on total fatty acid content in wild and farm raised salmon [59] indicated that fish diet can alter the fatty acid composition significantly. The total lipids in the farm raised salmon was found to be 12% of its body weight while wild salmon contained only 6% of total lipids. This significant increase in total lipid content in the farm raised salmons was attributed to the high vegetable oil content in its diet [59]. Sea lampreys feed only on blood of its host fish. Therefore, a comparison of its total lipid to the total lipid in fish species that feed on diets other than blood may not be relevant.

In summary, adult migratory sea lamprey skin contains significant amounts of triglycerides, cholesterol and free fatty acids relative to its skin mass. These lipid-soluble compounds are important sources of energy that may support migration, sexual maturation and spawning. In addition, we believe the large amount of TGs in sea lamprey skin may reduce frictional energy loss during swimming by increasing the water-repellency of the epidermis (an “easy to glide” benefit) that would also inhibit microbial attachment to the animal’s surface, perhaps establishing a strong selective pressure for accumulation of these water-repellent triglycerides in its skin. The presence of plasticizer pollutants in sea lamprey skin is an indication of the accumulation of contaminants in the Great Lakes regional waterways. Therefore, further studies are warranted to determine the bioaccumulation and biotransfer pathways of these pollutants, as well as any human health concerns since these compounds are likely accumulated in salmon, trout and other fame fish in the Great Lakes basin.

## Supporting Information

S1 File Nuclear Magnetic Resonance (NMR) and High Resolution Mass Spectrometry (HRMS) data of isolated compounds 1–4. (PDF)

S2 File Nuclear Magnetic Resonance (NMR) and Gas Chromatography Mass Spectrometry (GCMS) data of isolated compounds 5–10. (PDF)

S3 File Nuclear Magnetic Resonance (NMR) and Gas Chromatography Mass Spectrometry (GCMS) data of fatty acid mixtures. (PDF)
