# Supplementary material for: Chemical Characterization of Lipophilic Constituents in the Skin of Migratory Adult Sea Lamprey from the Great Lakes Region
Source: PLoS One. 2016 Dec 19;11(12):e0168609. doi: 10.1371/journal.pone.0168609 (PMC5167404; doi:10.1371/journal.pone.0168609)
Supplement: S1 File — (PDF) [file pone.0168609.s001.pdf]

Chemical Characterization of Lipophilic Constituents in the Skin of Migratory Adult Sea Lamprey from the Great Lakes Region

Amila A. Dissanayake,[1] Muraleedharan G. Nair,[1,3*]

Bioactive Natural Products and Phytoceuticals Laboratory, Department of Horticulture, Michigan State University, East Lansing, Michigan 48824, USA

Supplemental data

For compound **1**
Figure A. ^{1}H NMR spectrum of 1 in $CDCl_3$
Figure B. ^{13}C NMR spectrum of **1** in $CDCl_3$
Figure C. DEPT spectrum of **1** in $CDCl_3$
Figure D. HMBC spectrum of **1** in $CDCl_3$
Figure E. ESIMS (positive) spectrum **1**
Figure F. GCMS of the hexadecanoic acid, methyl ester

For compound **2**
Figure G. ^{1}H NMR spectrum **2** in $CDCl_3$
Figure H. ^{13}C NMR spectrum **2** in $CDCl_3$
Figure I. DEPT spectrum **2** in $CDCl_3$
Figure J. HMBC spectrum **2** in $CDCl_3$
Figure K. ESIMS (positive) spectrum **2**
Figure L. GCMS of the 9-octadecenoic acid (Z)-, methyl ester

For compound **3**
Figure M. ^{1}H NMR spectrum **3** in $CDCl_3$
Figure N. ^{13}C NMR spectrum **3** in $CDCl_3$
Figure O. DEPT spectrum **3** in $CDCl_3$
Figure P. HMBC spectrum **3** in $CDCl_3$
Figure Q. ESIMS (positive) spectrum **3**
Figure R. GCMS of the arachidonic acid, methyl ester

For compound **4**
Figure S. ^{1}H NMR spectrum **4** in $CDCl_3$
Figure T. ^{13}C NMR spectrum **4** in $CDCl_3$
Figure U. DEPT spectrum **4** in $CDCl_3$
Figure V. HMBC spectrum **4** in $CDCl_3$
Figure W. ESIMS (positive) spectrum **4**
Figure X. GCMS of the eicosapentaenoic acid, methyl ester

Figure A

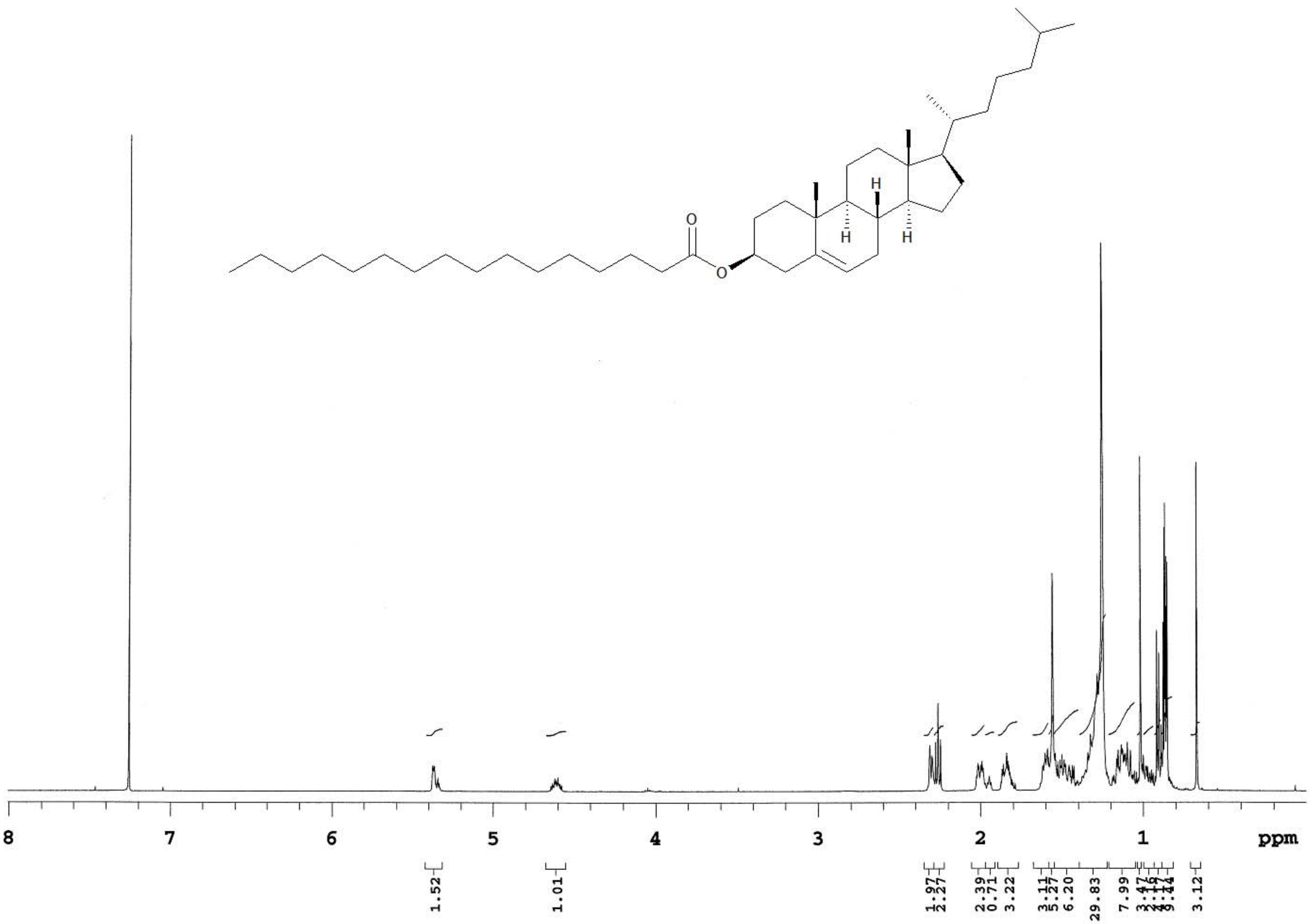

O
O
H
H
H
8
7
6
5
4
3
2
1
ppm
1.52
1.01
1.97
2.27
2.39
0.71
3.22
3.11
5.27
6.20
29.83
7.99
3.12

Figure B

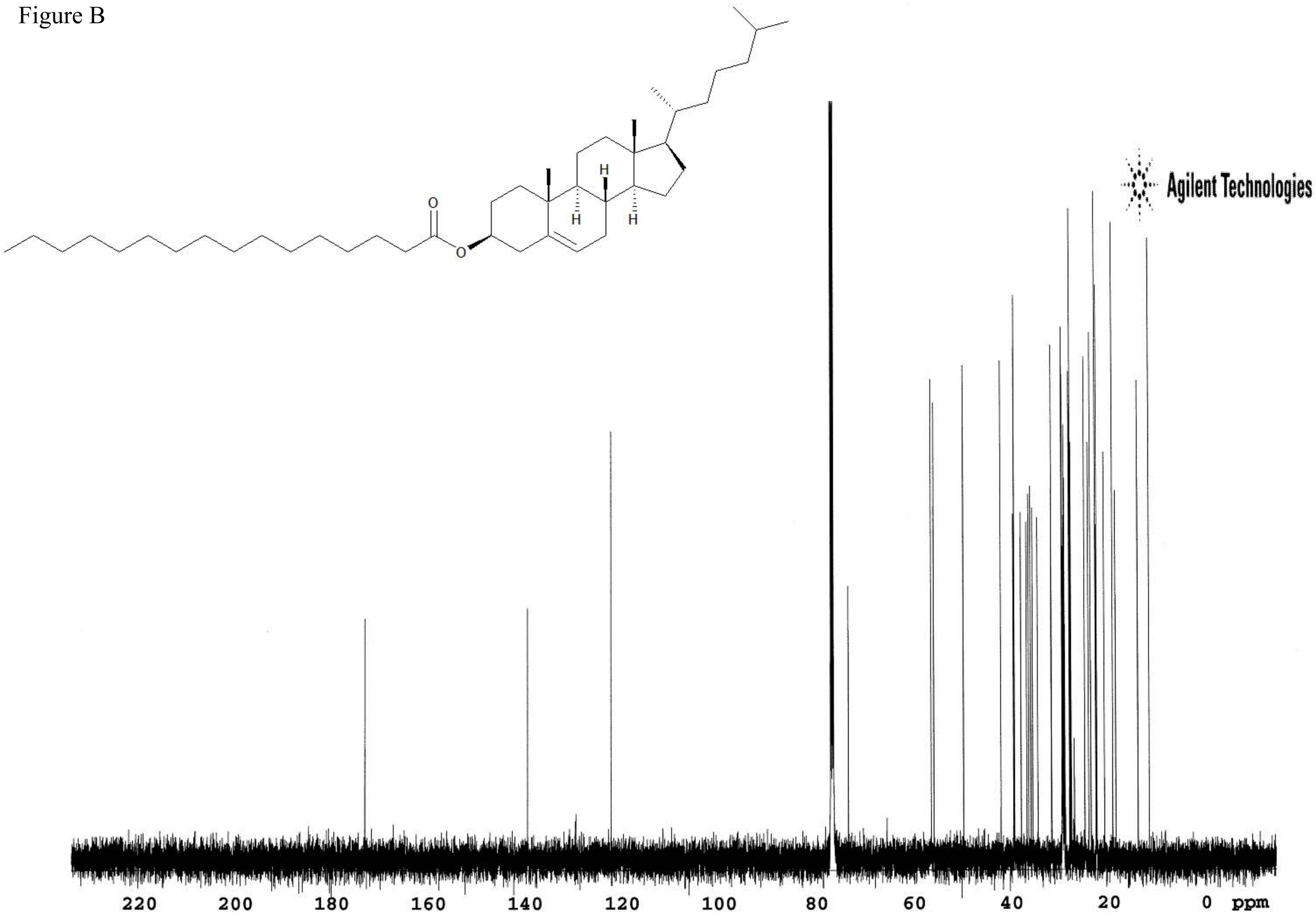

O
O
H
H
H
Agilent Technologies
220
200
180
160
140
120
100
80
60
40
20
0
ppm

Figure C

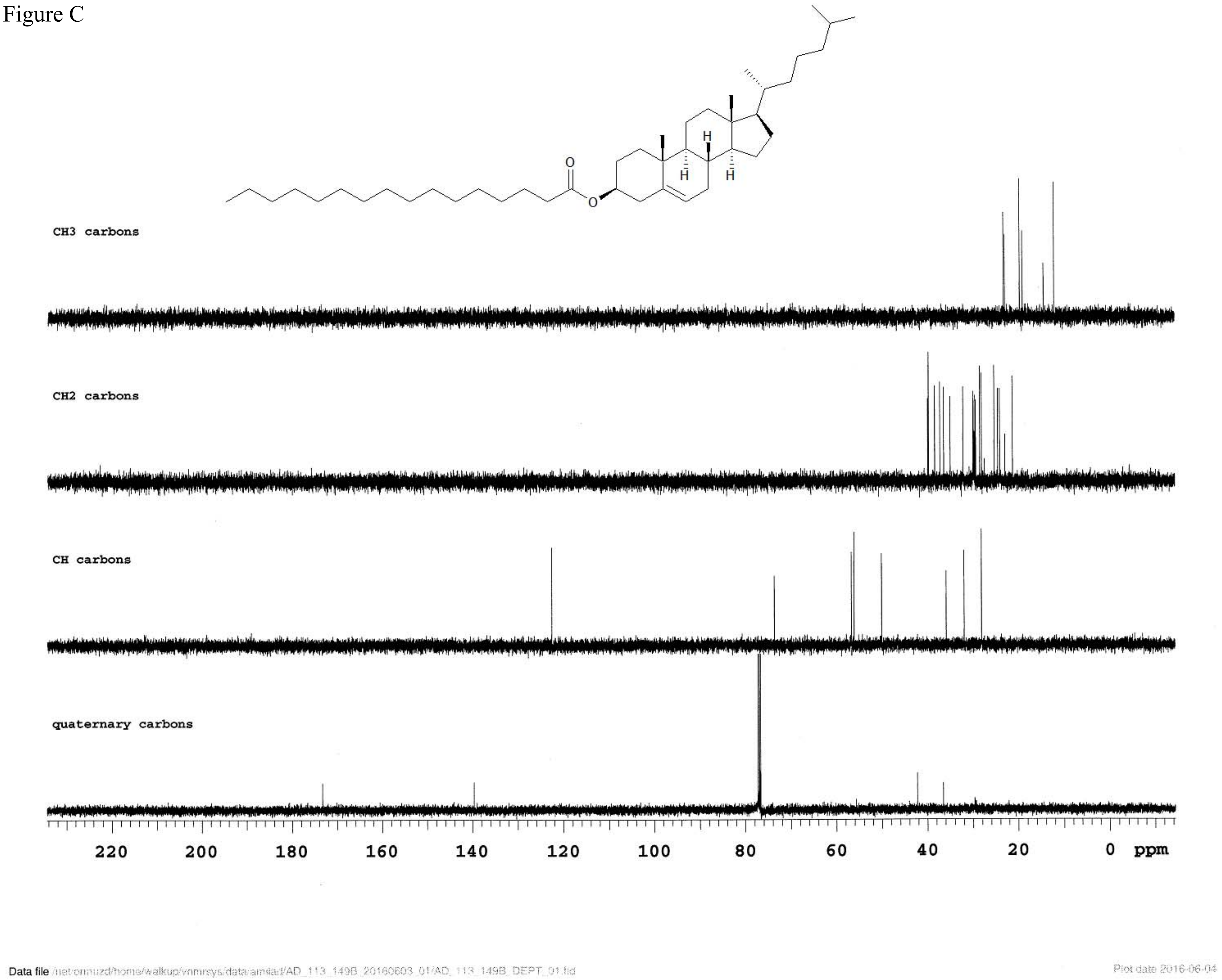

O
H
H
H
O
CH3 carbons
CH2 carbons
CH carbons
quaternary carbons
220
200
180
160
140
120
100
80
60
40
20
0
ppm
Data file /net/ormuzd/home/walkup/vnmrsys/data/amiad/AD_113_149B_20160603_01/AD_113_149B_DEPT_01.fid
Plot date 2016-06-04

Figure D

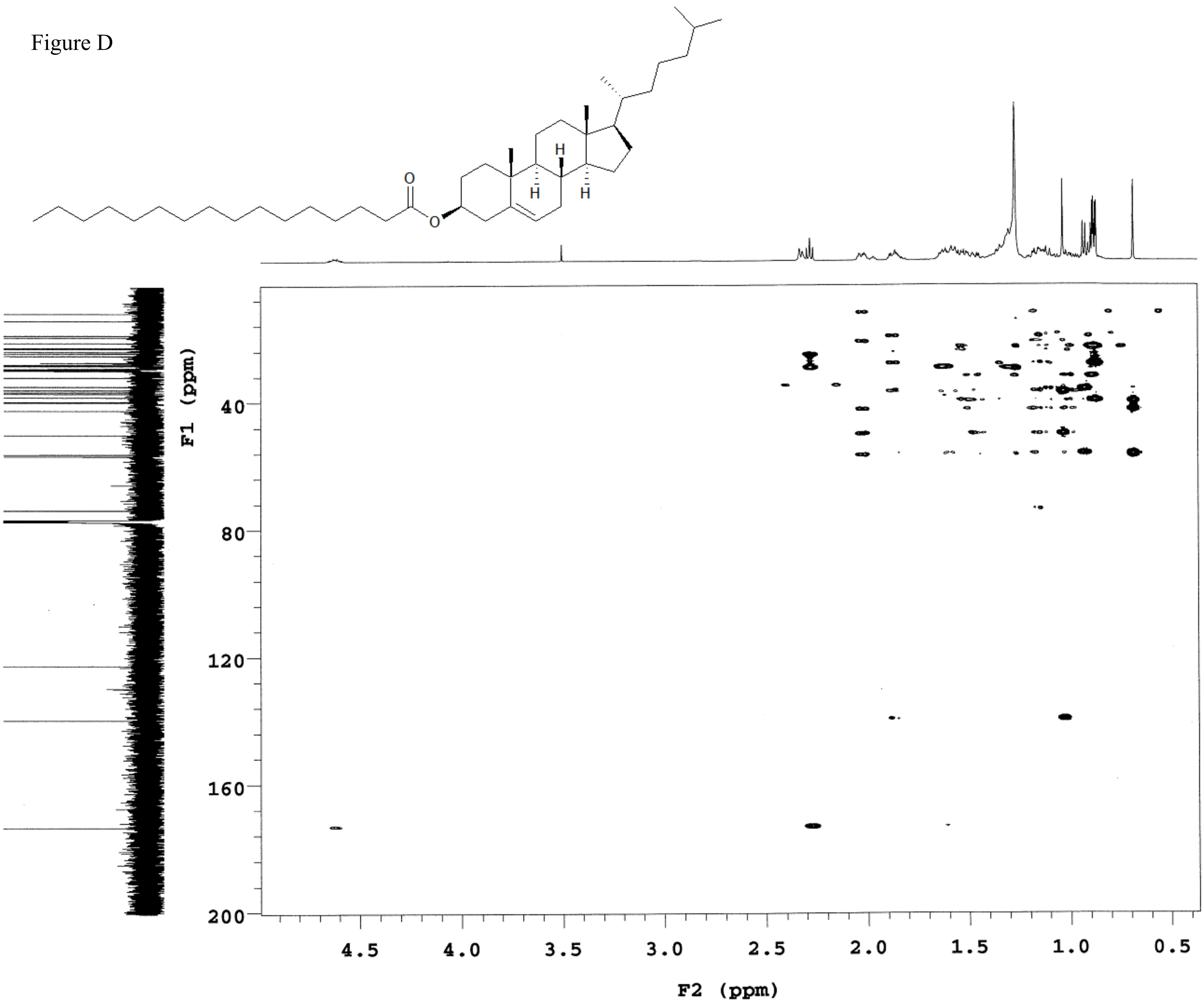

Figure E

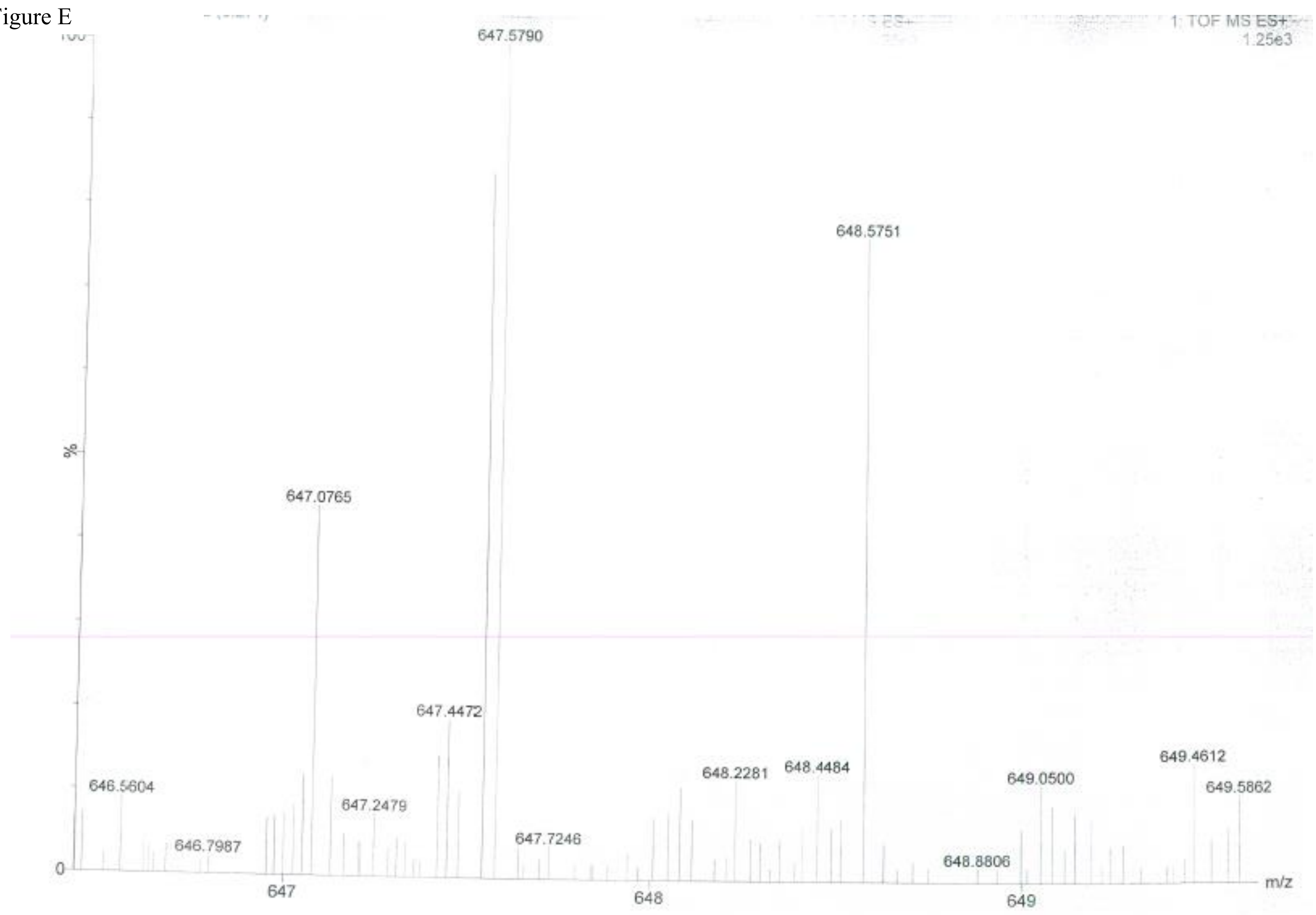
1: TOF MS ES+
1.25e3
647.5790
648.5751
%
647.0765
647.4472
646.5604
648.2281
648.4484
649.4612
649.0500
649.5862
647.2479
646.7987
647.7246
648.8806
0
647
648
649
m/z

Figure F

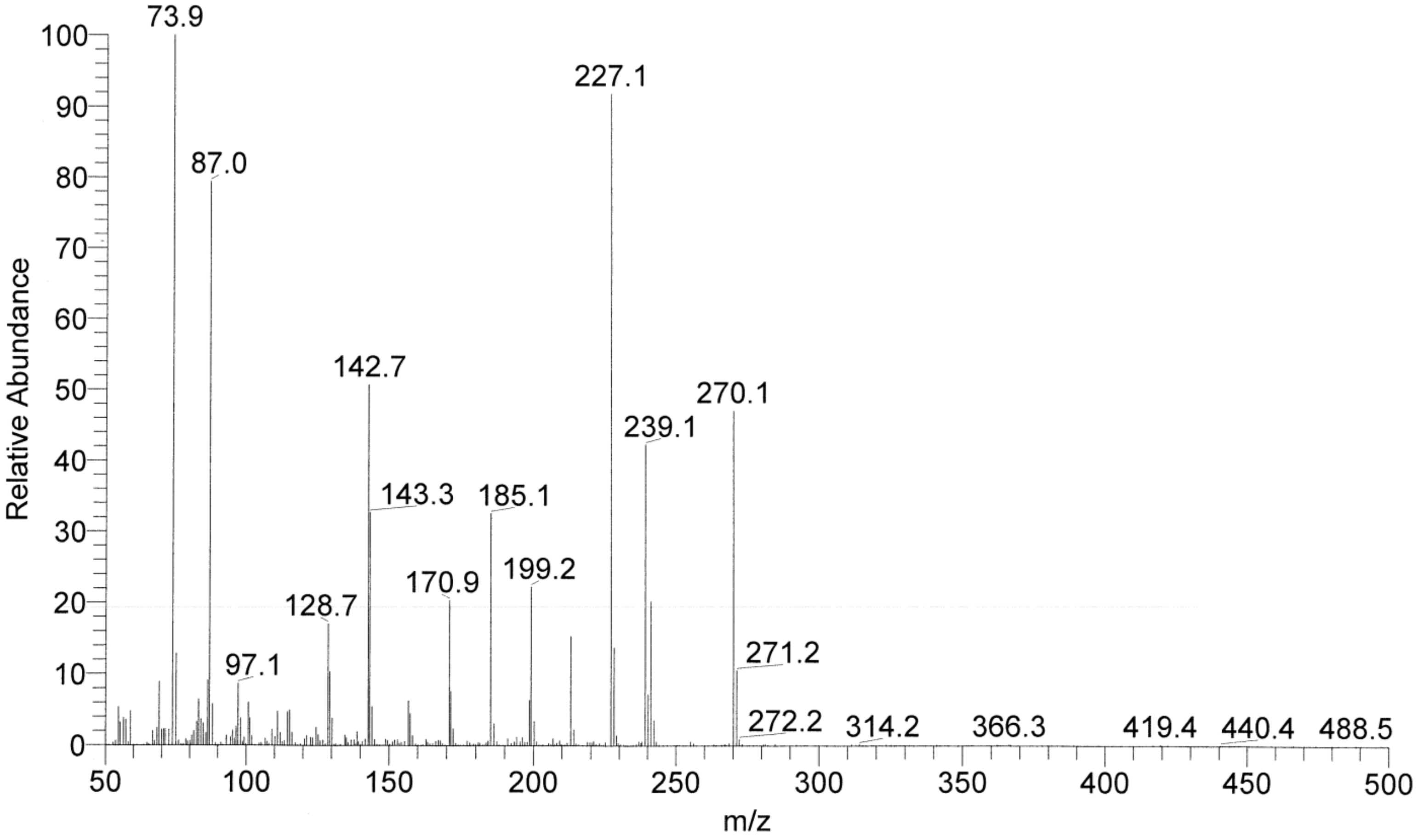

73.9
227.1
87.0
142.7
270.1
239.1
143.3
185.1
199.2
170.9
128.7
97.1
271.2
272.2
314.2
366.3
419.4
440.4
488.5
Relative Abundance
100
90
80
70
60
50
40
30
20
10
0
50
100
150
200
250
300
350
400
450
500
m/z

Figure G

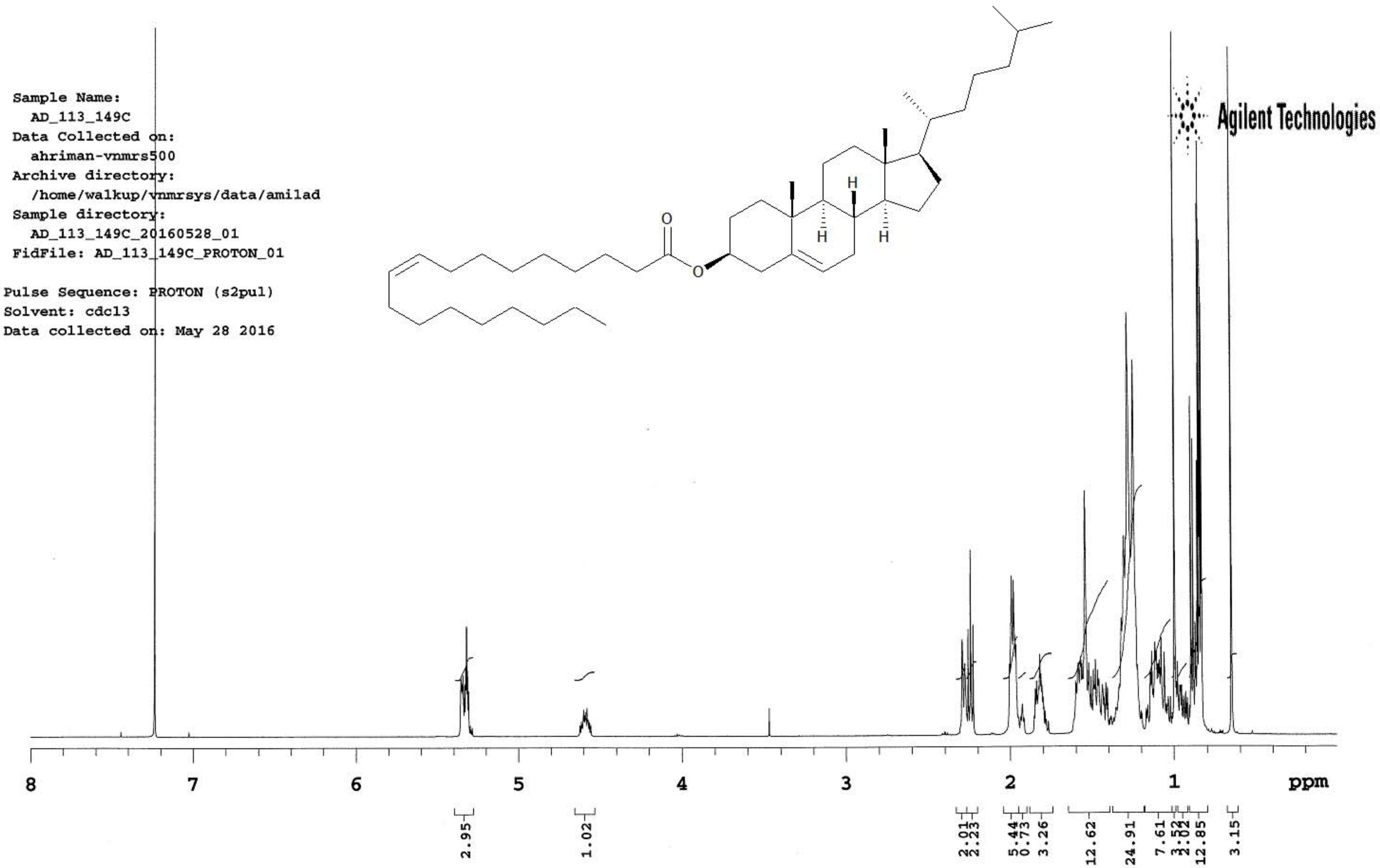
Sample Name:
AD_113_149C
Data Collected on:
ahriman-vnmrs500
Archive directory:
/home/walkup/vnmrsys/data/amilad
Sample directory:
AD_113_149C_20160528_01
FidFile: AD_113_149C_PROTON_01
Pulse Sequence: PROTON (s2pul)
Solvent: cdcl3
Data collected on: May 28 2016
Agilent Technologies
O
O
H
H
H
8
7
6
5
4
3
2
1
ppm
2.95
1.02
2.01
2.23
5.44
0.73
3.26
12.62
24.91
7.61
3.52
3.02
12.85
3.15

Figure H

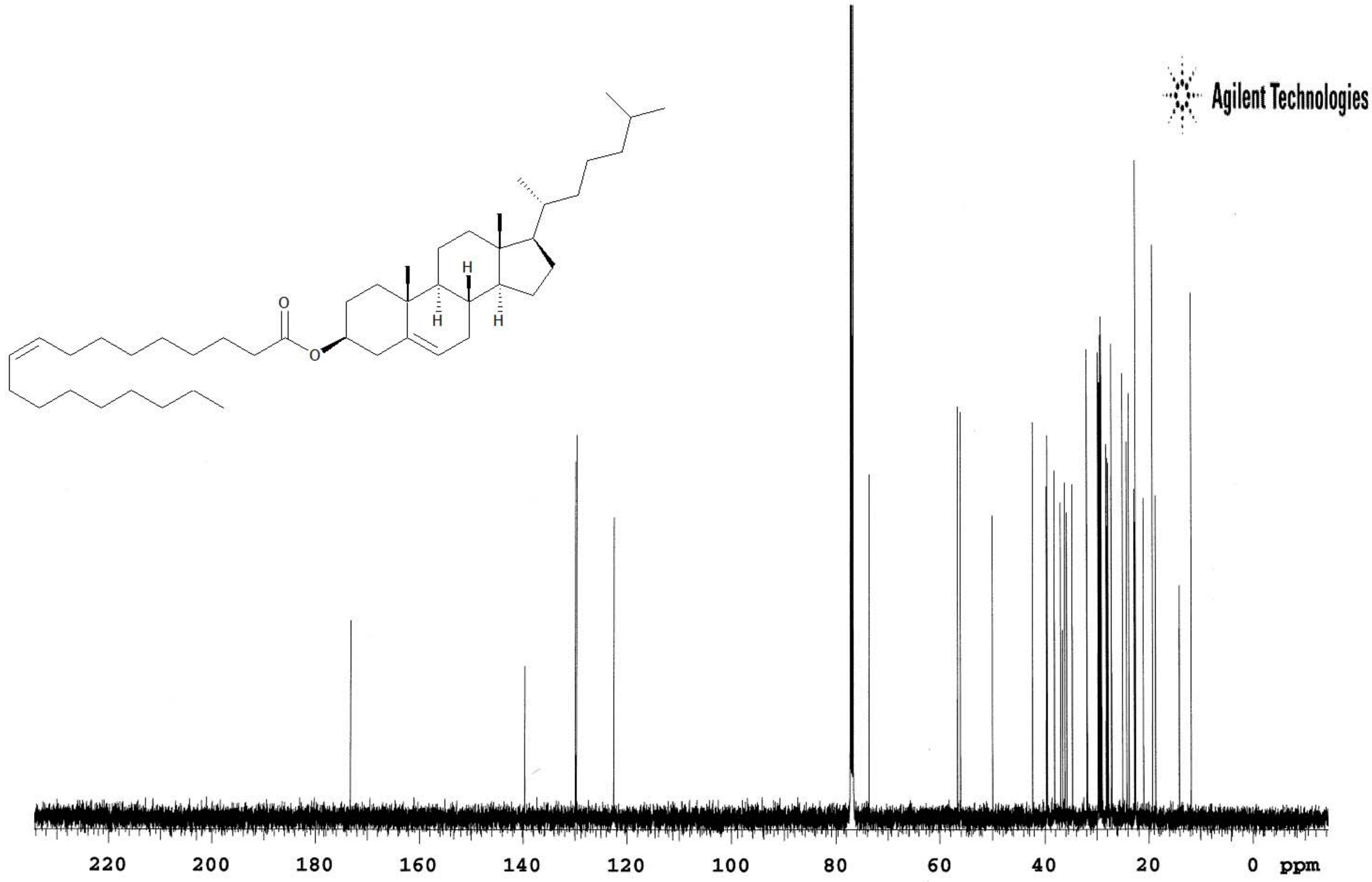

Figure I

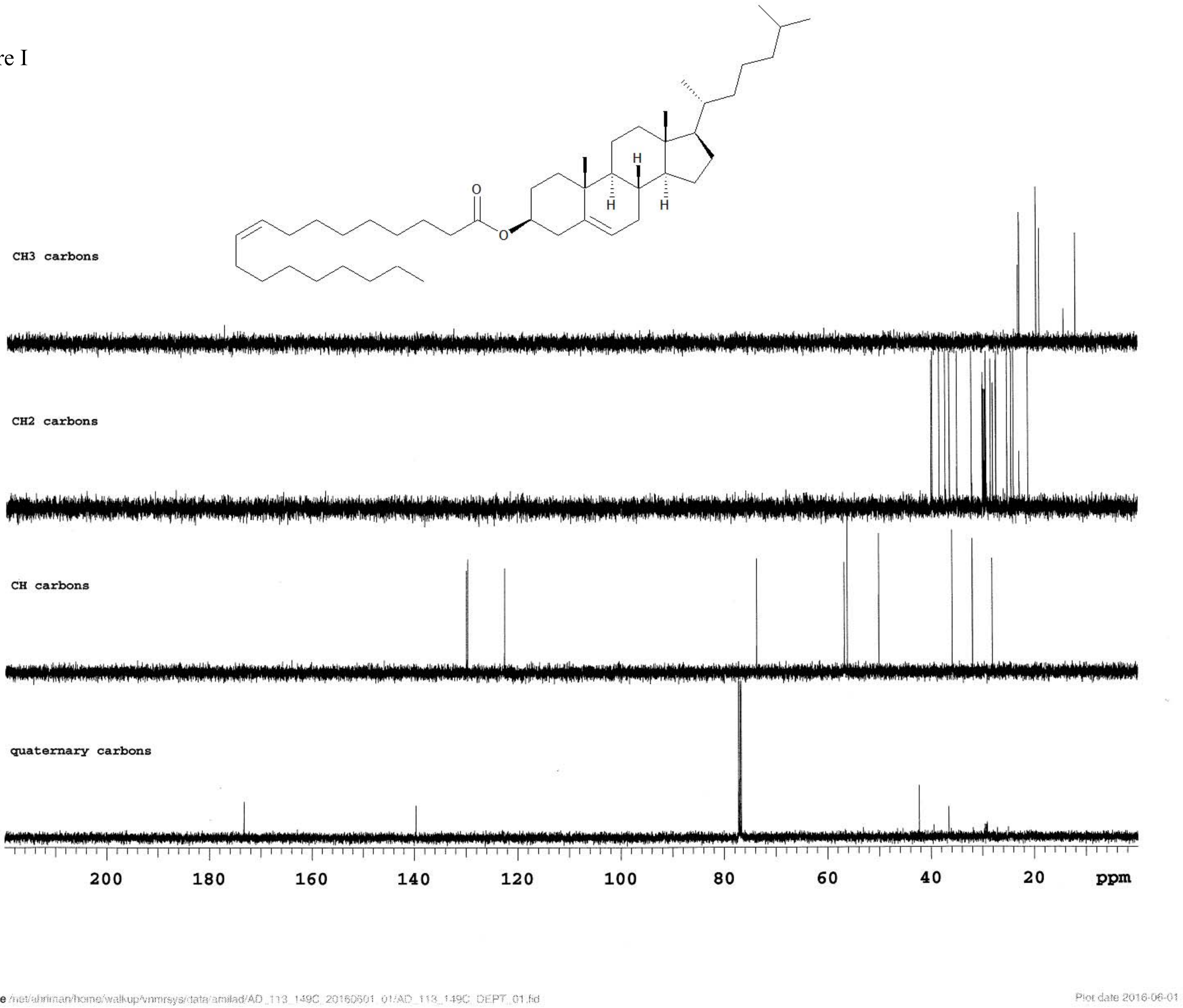
O
O
H
H
H
CH3 carbons
CH2 carbons
CH carbons
quaternary carbons
200
180
160
140
120
100
80
60
40
20
ppm
Data file /net/ahriman/home/walkup/vnmrsys/data/amilad/AD_113_149C_20160601_01/AD_113_149C_DEPT_01.fid
Plot date 2016-06-01

Figure J

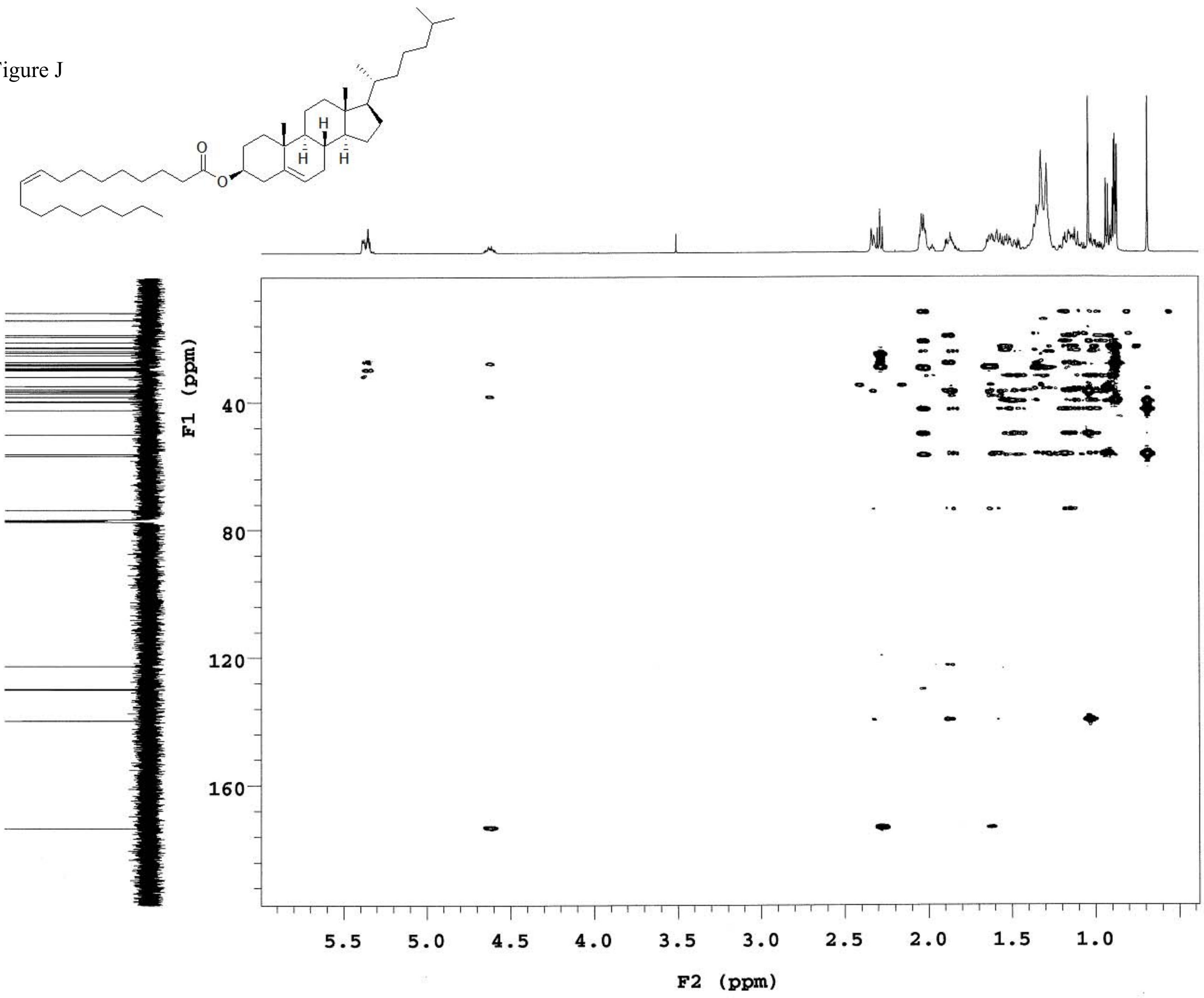

Figure K

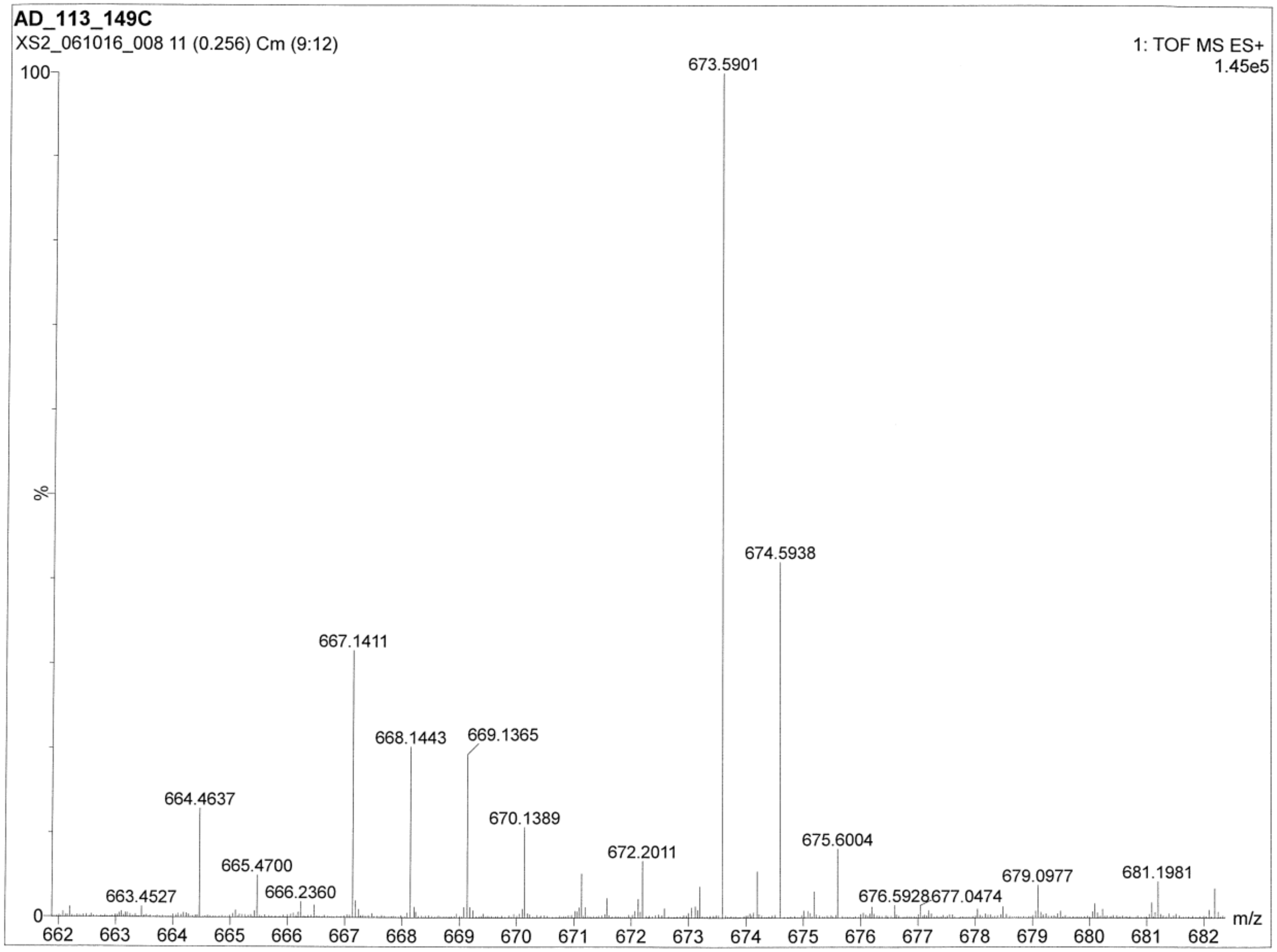
AD_113_149C
XS2_061016_008 11 (0.256) Cm (9:12)
1: TOF MS ES+
1.45e5
673.5901
674.5938
667.1411
668.1443
669.1365
664.4637
670.1389
672.2011
675.6004
665.4700
666.2360
663.4527
676.5928
677.0474
679.0977
681.1981
100
%
0
662
663
664
665
666
667
668
669
670
671
672
673
674
675
676
677
678
679
680
681
682
m/z

Figure L

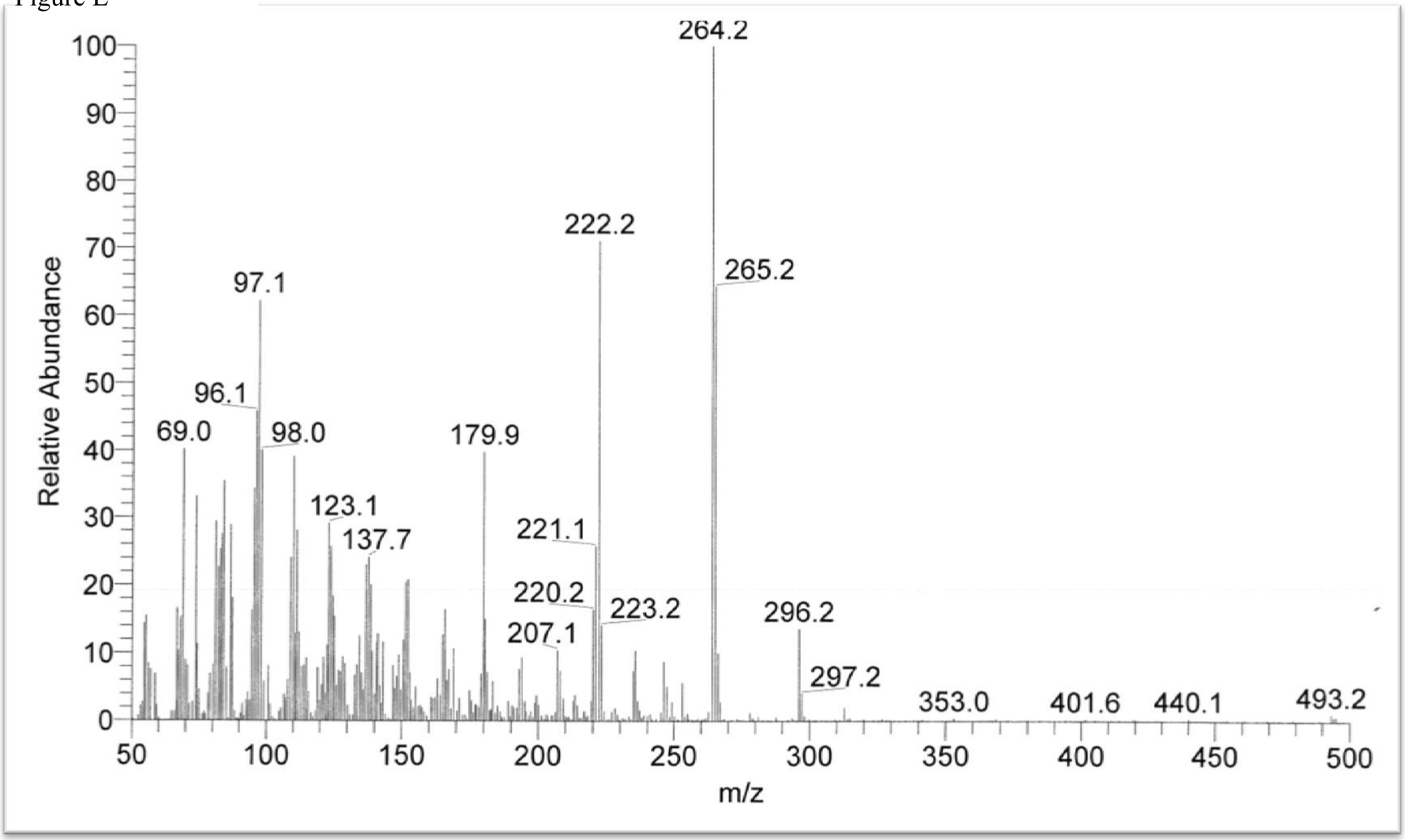

264.2
222.2
265.2
97.1
96.1
69.0
98.0
179.9
123.1
137.7
221.1
220.2
223.2
207.1
296.2
297.2
353.0
401.6
440.1
493.2
Relative Abundance
m/z

Figure M

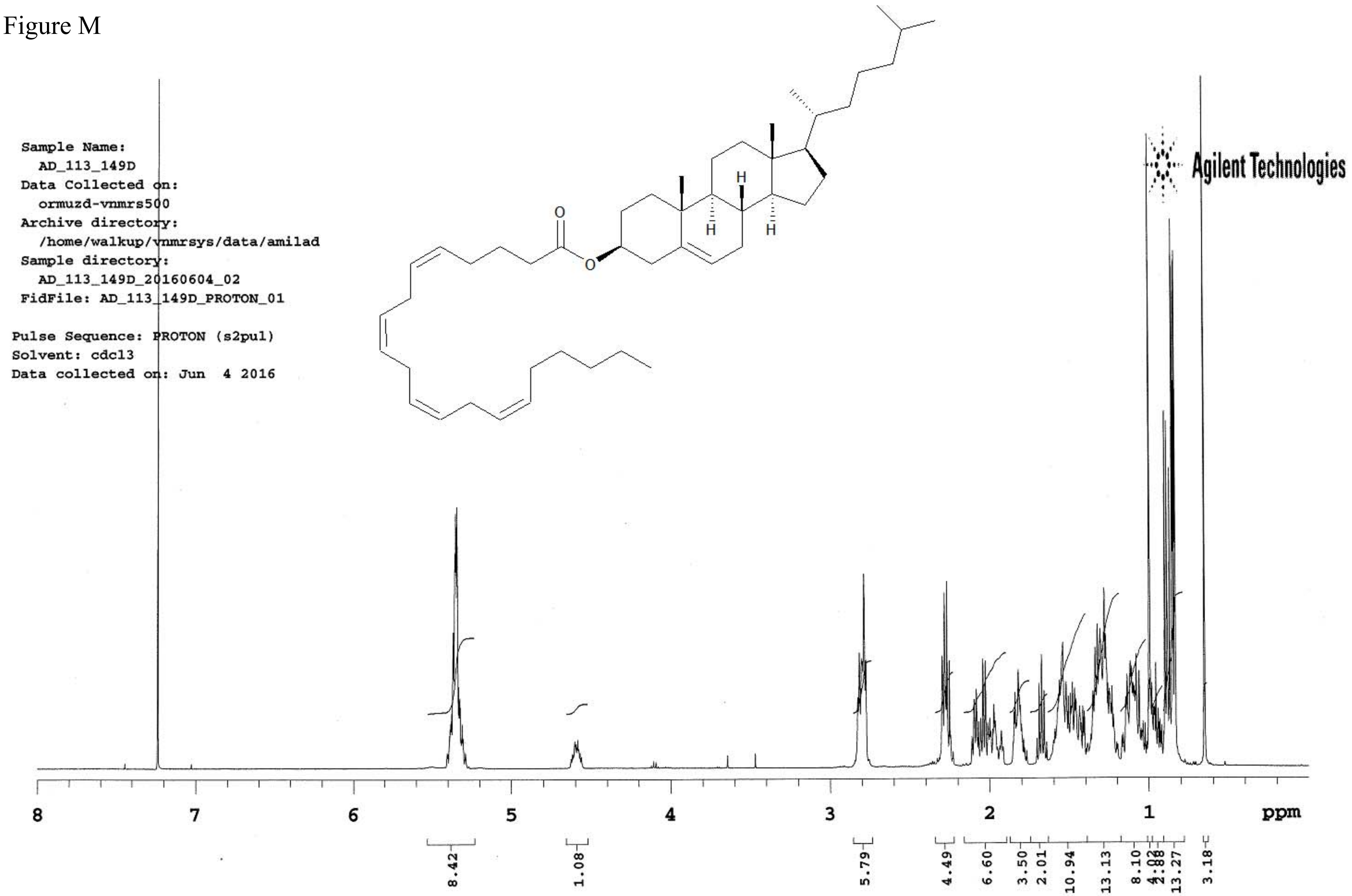
Sample Name:
AD_113_149D
Data Collected on:
ormuzd-vnmrs500
Archive directory:
/home/walkup/vnmrsys/data/amilad
Sample directory:
AD_113_149D_20160604_02
FidFile: AD_113_149D_PROTON_01
Pulse Sequence: PROTON (s2pul)
Solvent: cdcl3
Data collected on: Jun 4 2016
Agilent Technologies
8
7
6
5
4
3
2
1
ppm
8.42
1.08
5.79
4.49
6.60
3.50
2.01
10.94
13.13
8.10
13.27
3.18

Figure N

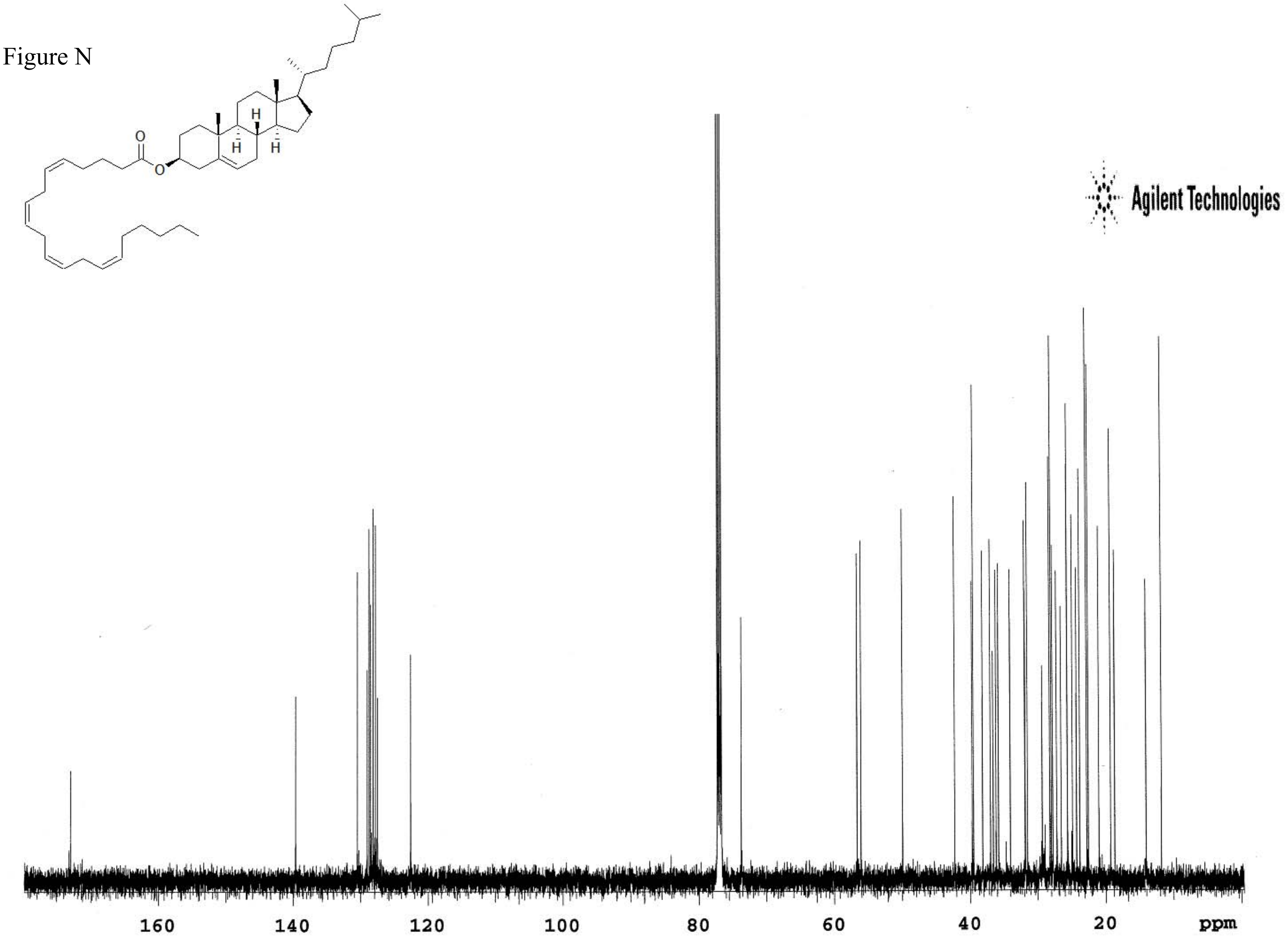

Figure O

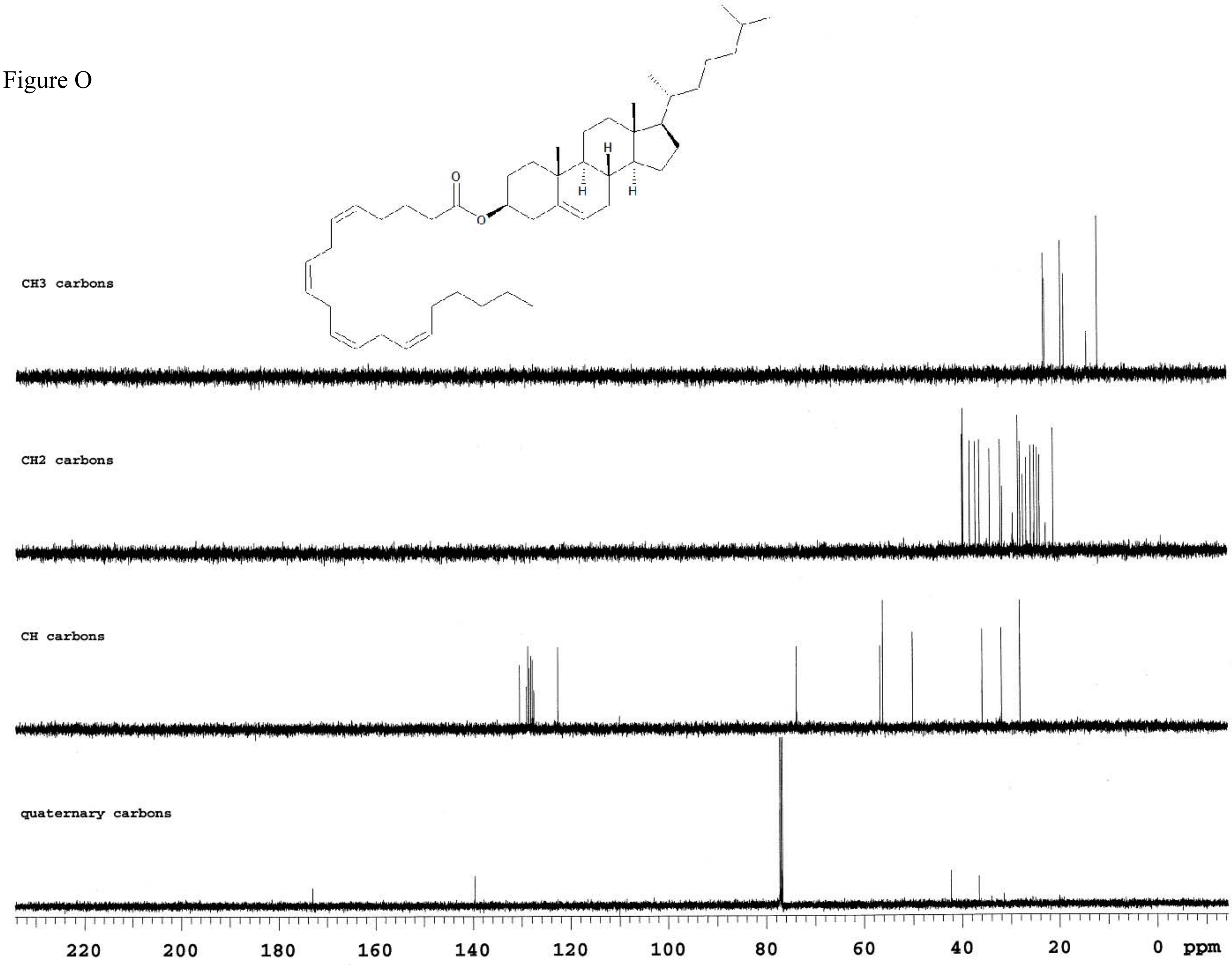

Data file /net/ormuzd/home/walkup/vnmrsys/data/amied/AD_113_149D_20160604_02/AD_113_149D_DEPT_01.fid

Plot date 2016-06-05

Figure P

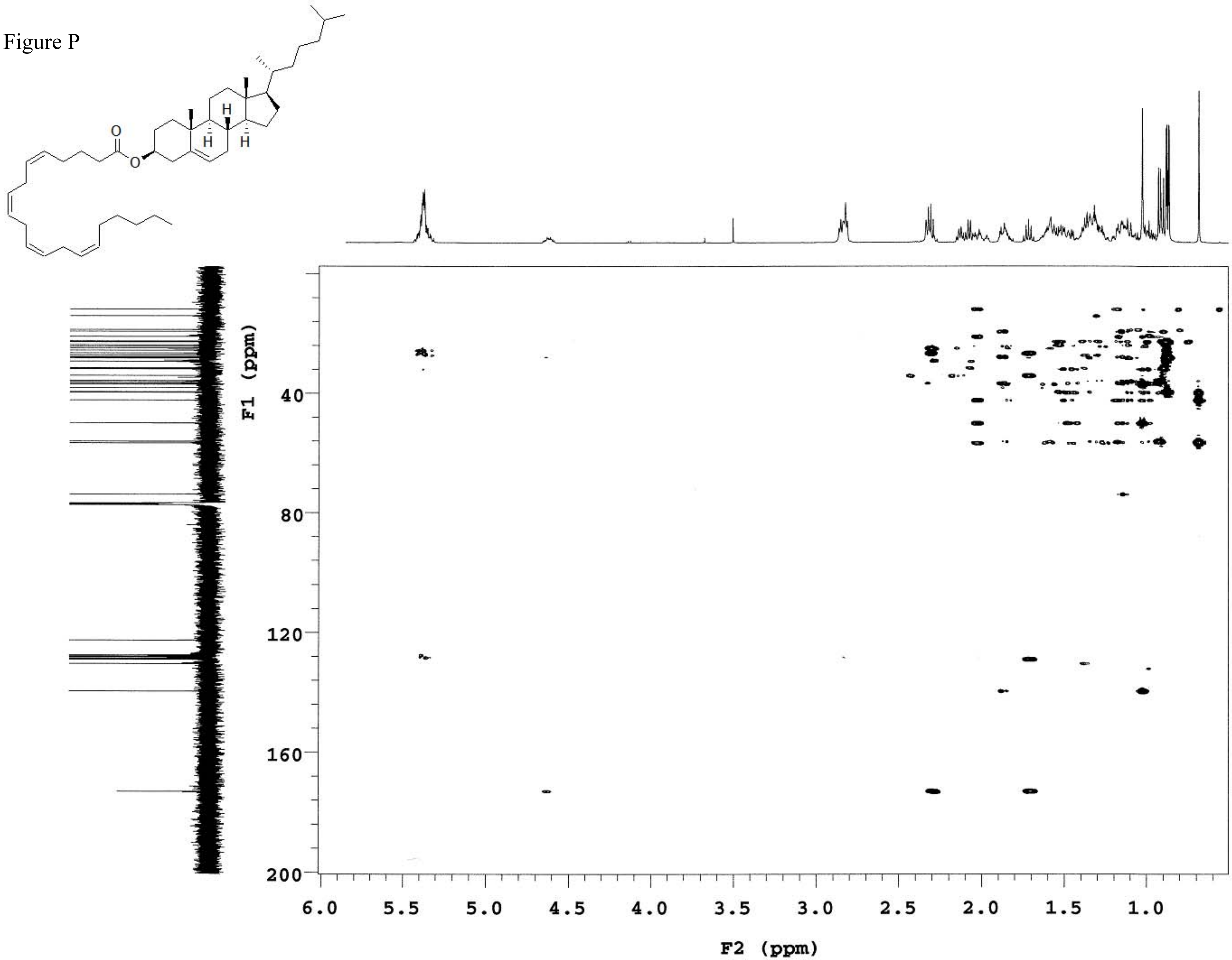

Figure Q

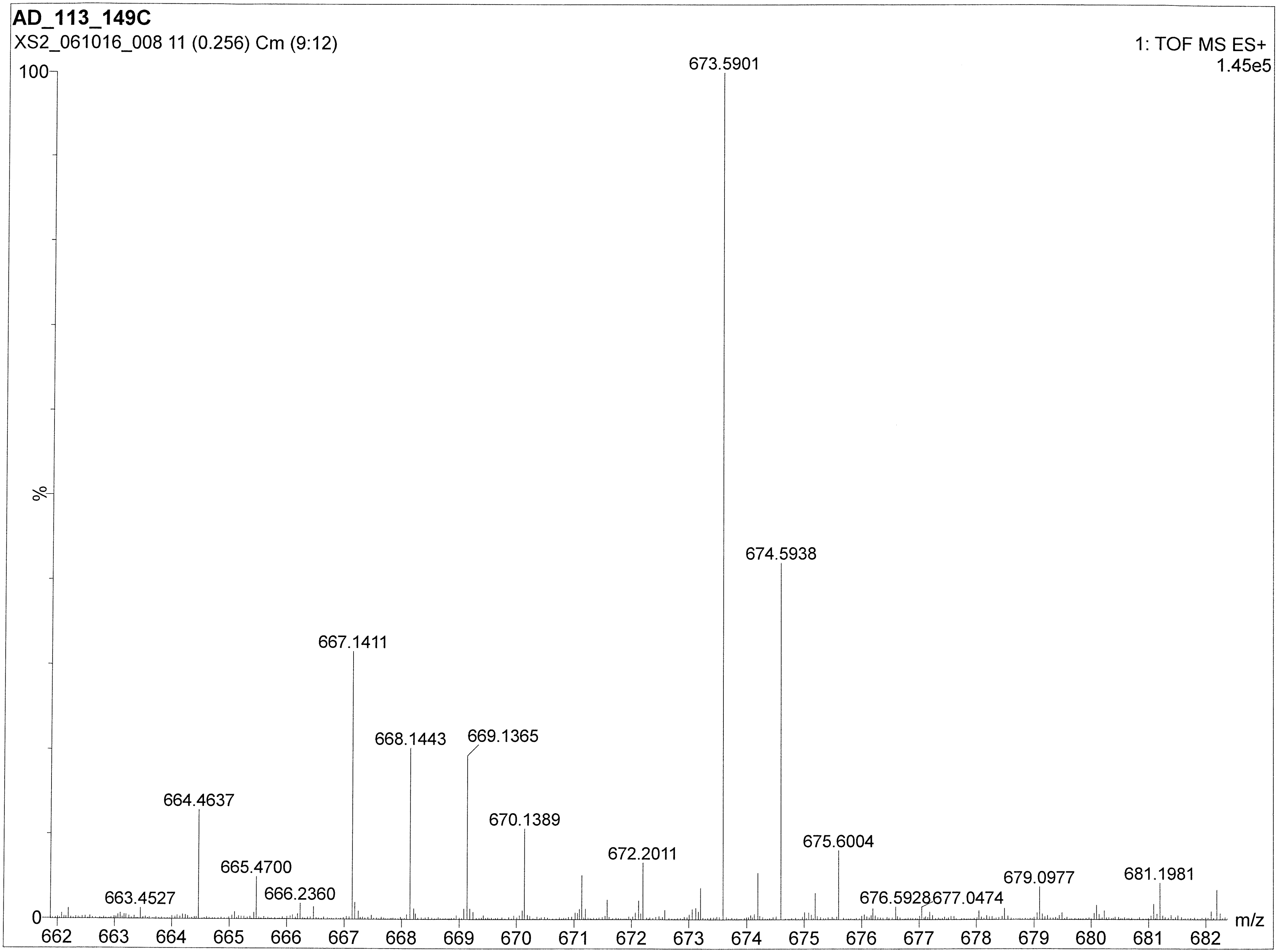

AD_113_149C
XS2_061016_008 11 (0.256) Cm (9:12)
1: TOF MS ES+
1.45e5
100
%
0
673.5901
674.5938
667.1411
668.1443
669.1365
664.4637
670.1389
672.2011
675.6004
665.4700
666.2360
663.4527
676.5928
677.0474
679.0977
681.1981
m/z
662
663
664
665
666
667
668
669
670
671
672
673
674
675
676
677
678
679
680
681
682

Figure R

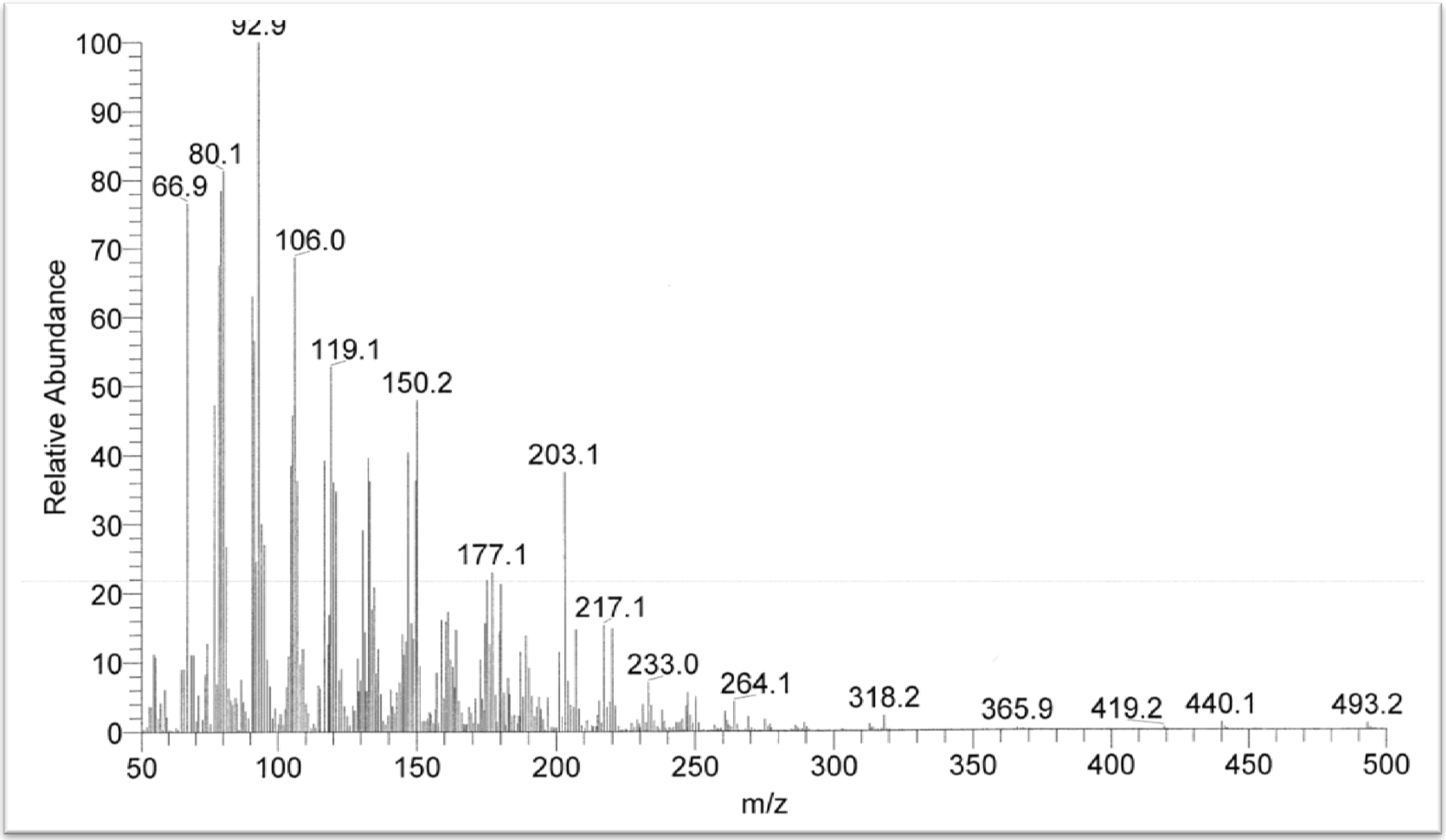

92.9
80.1
66.9
106.0
119.1
150.2
203.1
177.1
217.1
233.0
264.1
318.2
365.9
419.2
440.1
493.2
Relative Abundance
m/z
100
90
80
70
60
50
40
30
20
10
0
50
100
150
200
250
300
350
400
450
500

Figure S

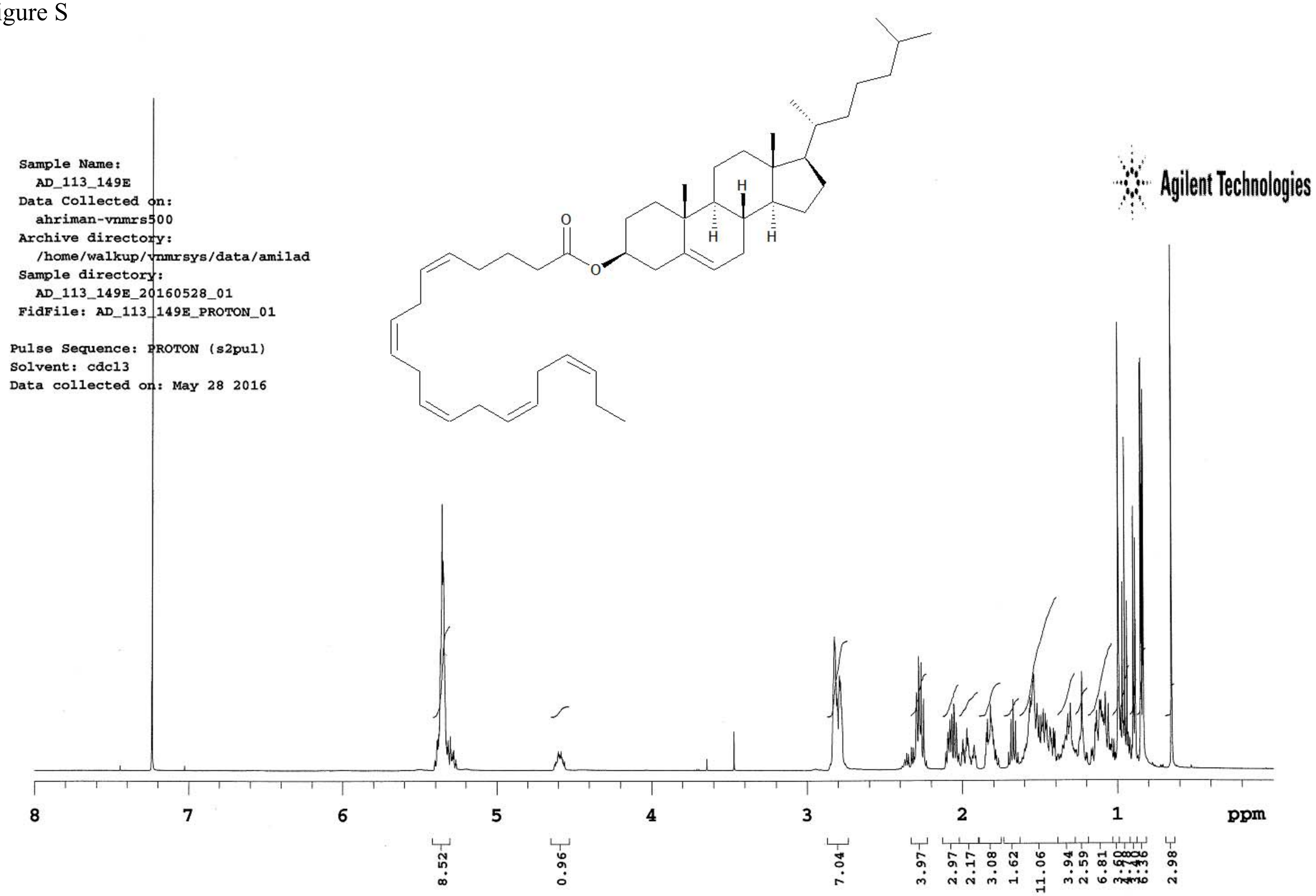
Sample Name:
AD_113_149E
Data Collected on:
ahriman-vnmrs500
Archive directory:
/home/walkup/vnmrsys/data/amilad
Sample directory:
AD_113_149E_20160528_01
FidFile: AD_113_149E_PROTON_01
Pulse Sequence: PROTON (s2pul)
Solvent: cdcl3
Data collected on: May 28 2016
Agilent Technologies
O
O
H
H
H
8
7
6
5
4
3
2
1
ppm
8.52
0.96
7.04
3.97
2.97
2.17
3.08
1.62
11.06
3.94
2.59
6.81
2.98

Figure T

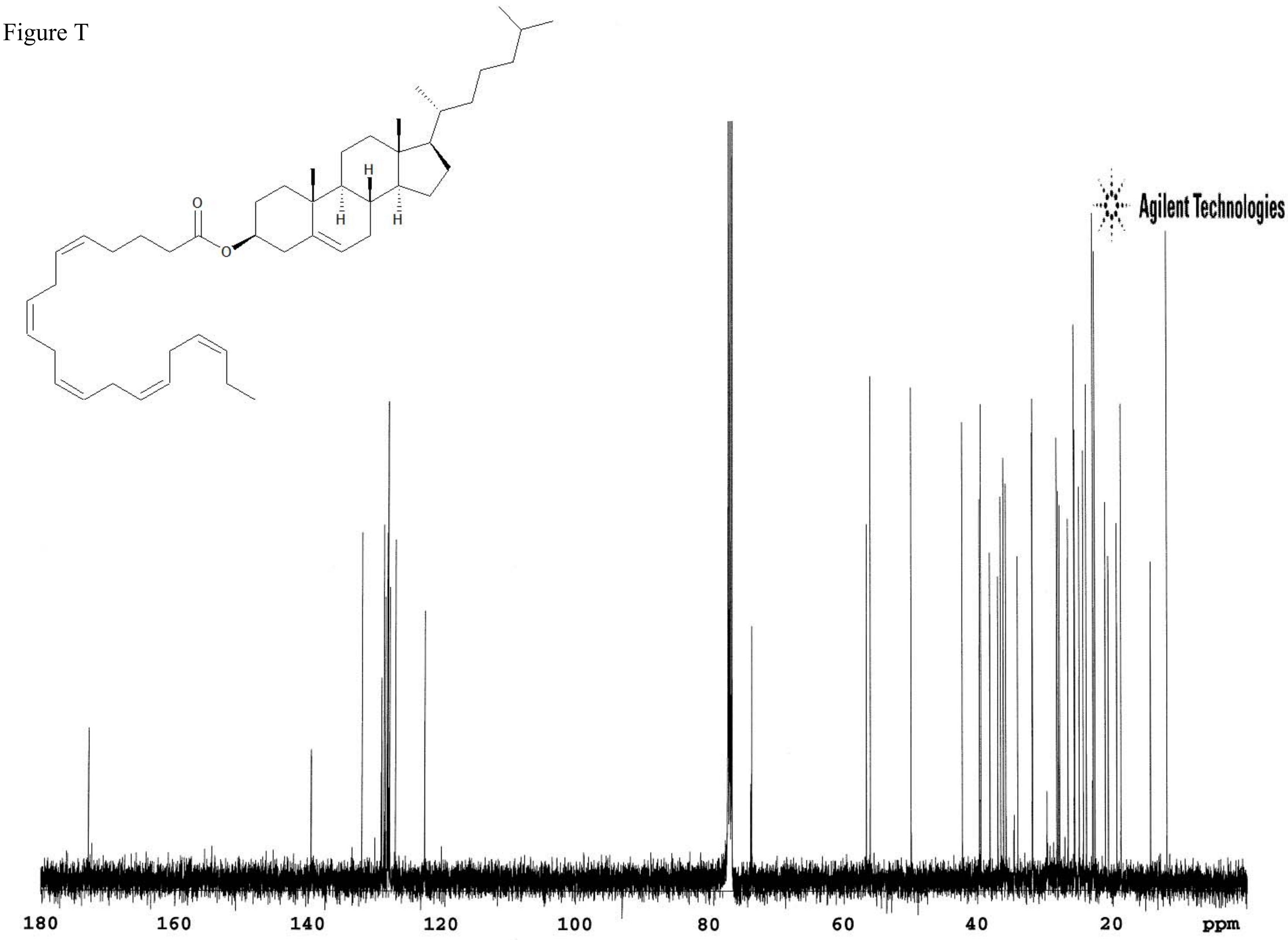

Figure U

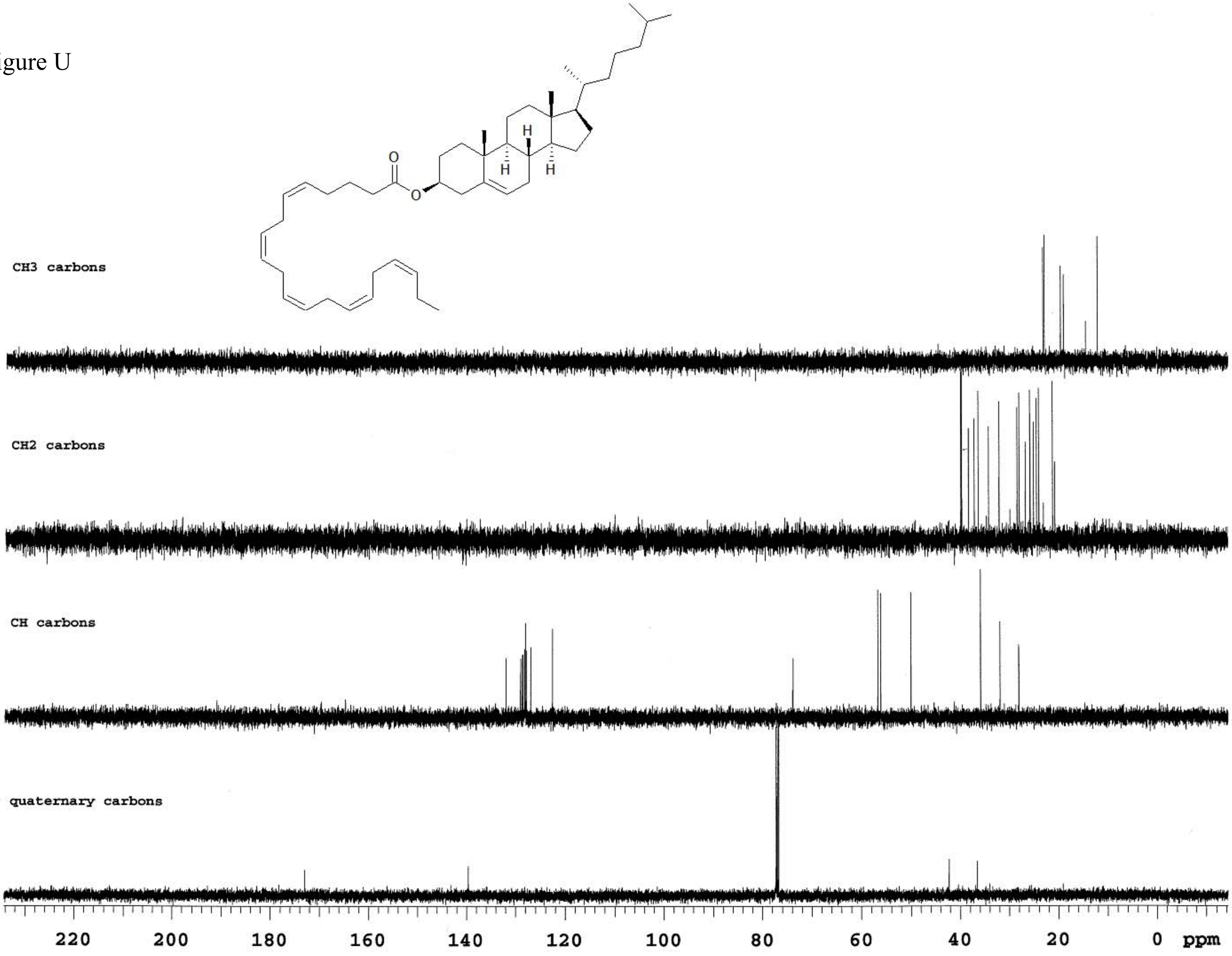

Data file /net/ahriman/home/walkup/vnmrsys/data/amilad/AD_113_149E_20160601_01/AD_113_149E_DEPT_01.fid

Plot date 2016-06-01

Figure V

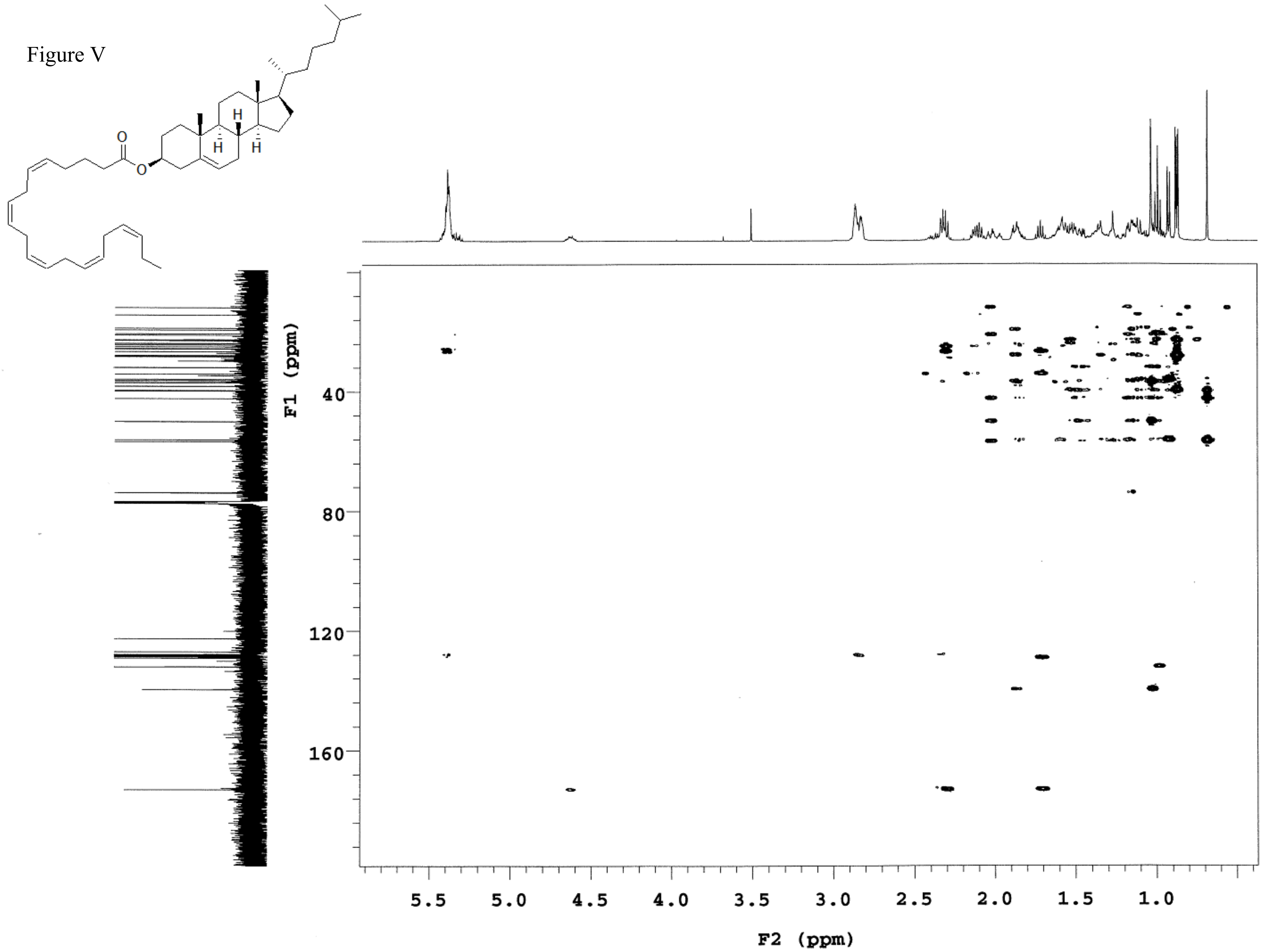

Figure W

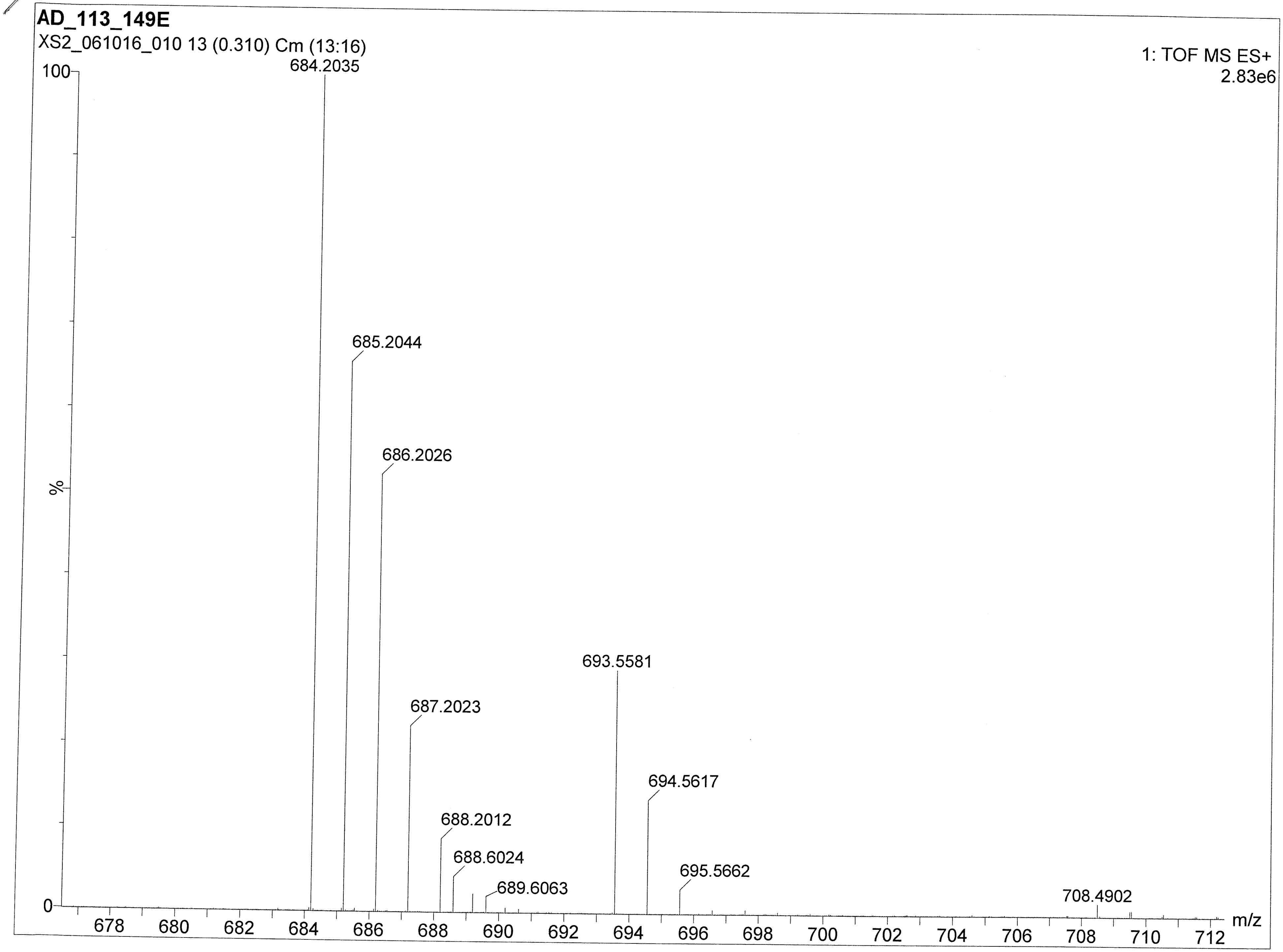

AD_113_149E
XS2_061016_010 13 (0.310) Cm (13:16)
1: TOF MS ES+
2.83e6
100
%
0
684.2035
685.2044
686.2026
687.2023
688.2012
688.6024
689.6063
693.5581
694.5617
695.5662
708.4902
678
680
682
684
686
688
690
692
694
696
698
700
702
704
706
708
710
712
m/z

Figure X

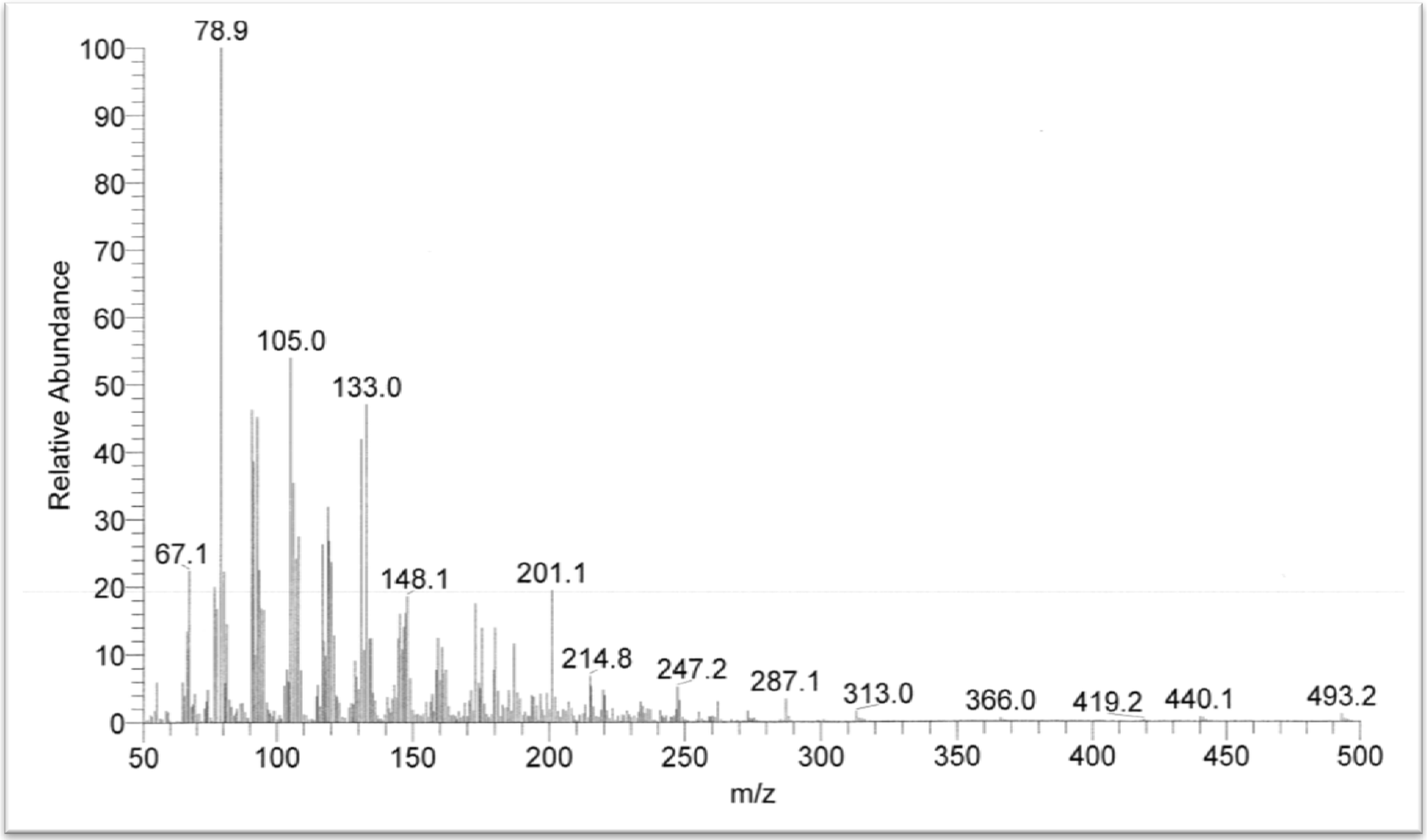

Relative Abundance
m/z
78.9
105.0
133.0
67.1
148.1
201.1
214.8
247.2
287.1
313.0
366.0
419.2
440.1
493.2
100
90
80
70
60
50
40
30
20
10
0
50
100
150
200
250
300
350
400
450
500
