# Supplementary material for: Chemical Characterization of Lipophilic Constituents in the Skin of Migratory Adult Sea Lamprey from the Great Lakes Region
Source: PLoS One. 2016 Dec 19;11(12):e0168609. doi: 10.1371/journal.pone.0168609 (PMC5167404; doi:10.1371/journal.pone.0168609)
Supplement: S2 File — (PDF) [file pone.0168609.s002.pdf]

Chemical Characterization of Lipophilic Constituents in the Skin of Migratory Adult Sea Lamprey from the Great Lakes Region

Amila A. Dissanayake,[1] Muraleedharan G. Nair,[1,3*]

Bioactive Natural Products and Phytoceuticals Laboratory, Department of Horticulture, Michigan State University, East Lansing, Michigan 48824, USA

Supplemental data

For compound **5**
Figure A. ^{1}H NMR spectrum **5** in $CDCl_3$
Figure B. ^{13}C NMR spectrum of **5** in $CDCl_3$
Figure C. HMBC spectrum **5** in $CDCl_3$
Figure D. GC profile of methyl esters of compound **5**
Figure E. GCMS of the hexadecanoic acid, methyl ester

For triglyceride mixture **F**
Figure F. GCMS of the 9-hexadecenoic acid, methyl ester (Z)
Figure G. ^{1}H NMR spectrum of triglyceride mixture **F** in $CDCl_3$
Figure H. GC profile of methyl esters of triglyceride mixture

For compound **6**
Figure I. ^{1}H NMR spectrum **6** in $CDCl_3$
Figure J. ^{13}C NMR spectrum **6** in $CDCl_3$
Figure K. HSQC spectrum **6** in $CDCl_3$
Figure L. GCMS of compound **6**

For compound **7**
Figure M. ^{1}H NMR spectrum **7** in $CDCl_3$
Figure N. ^{13}C NMR spectrum **7** in $CDCl_3$
Figure O. HSQC spectrum **7** in $CDCl_3$
Figure P. HMBC spectrum **7** in $CDCl_3$
Figure Q. GCMS of the compound **7**

For compound **8**
Figure R. ^{1}H NMR spectrum **8** in $CDCl_3$
Figure S. ^{13}C NMR spectrum **8** in $CDCl_3$

For compound **9**
Figure T. ^{1}H NMR spectrum **9** in $CDCl_3$
Figure U. ^{13}C NMR spectrum **9** in $CDCl_3$

For compound **10**
Figure V. ^{1}H NMR spectrum of **10** in $CDCl_3$

Figure A

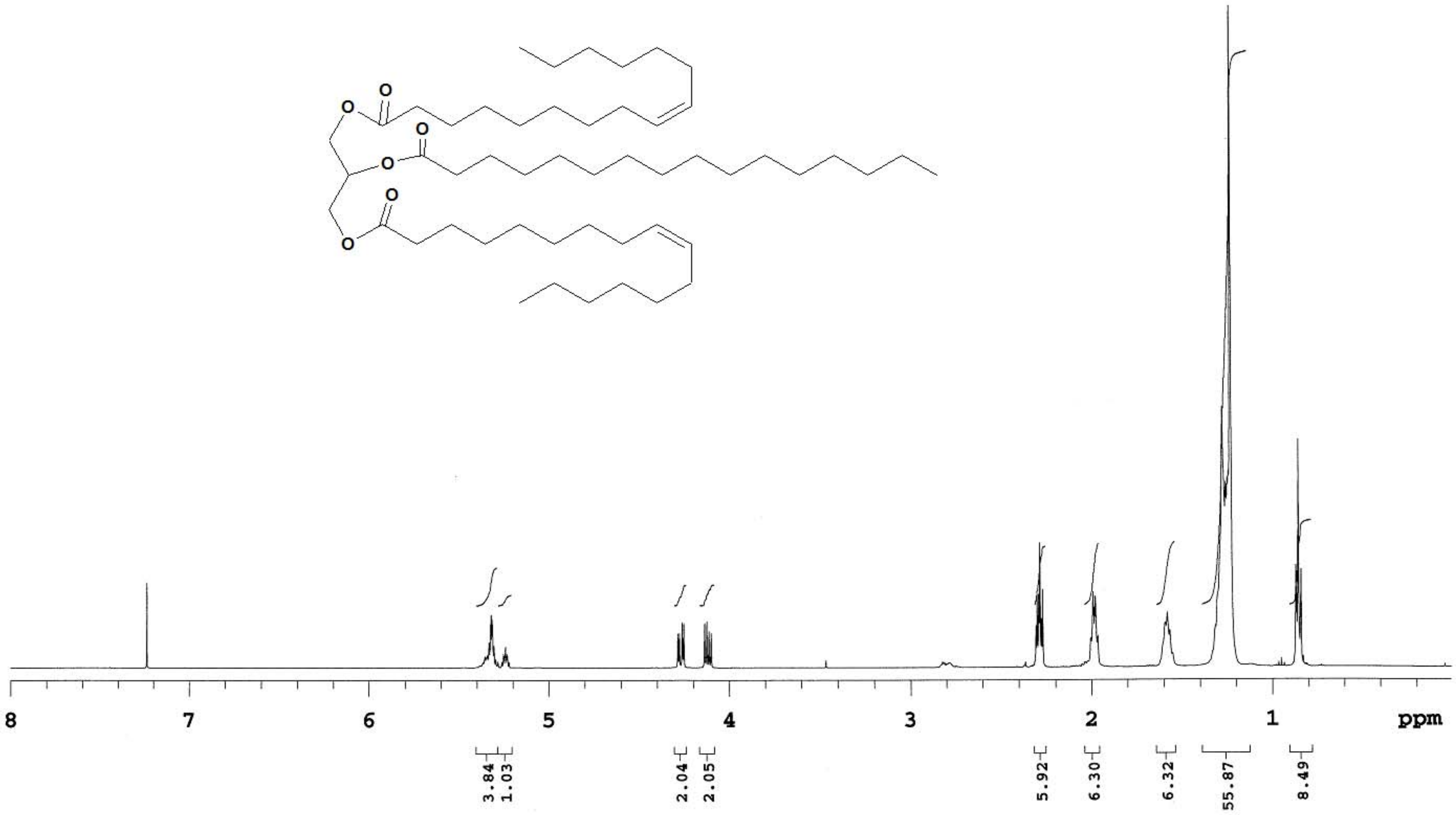

Figure B

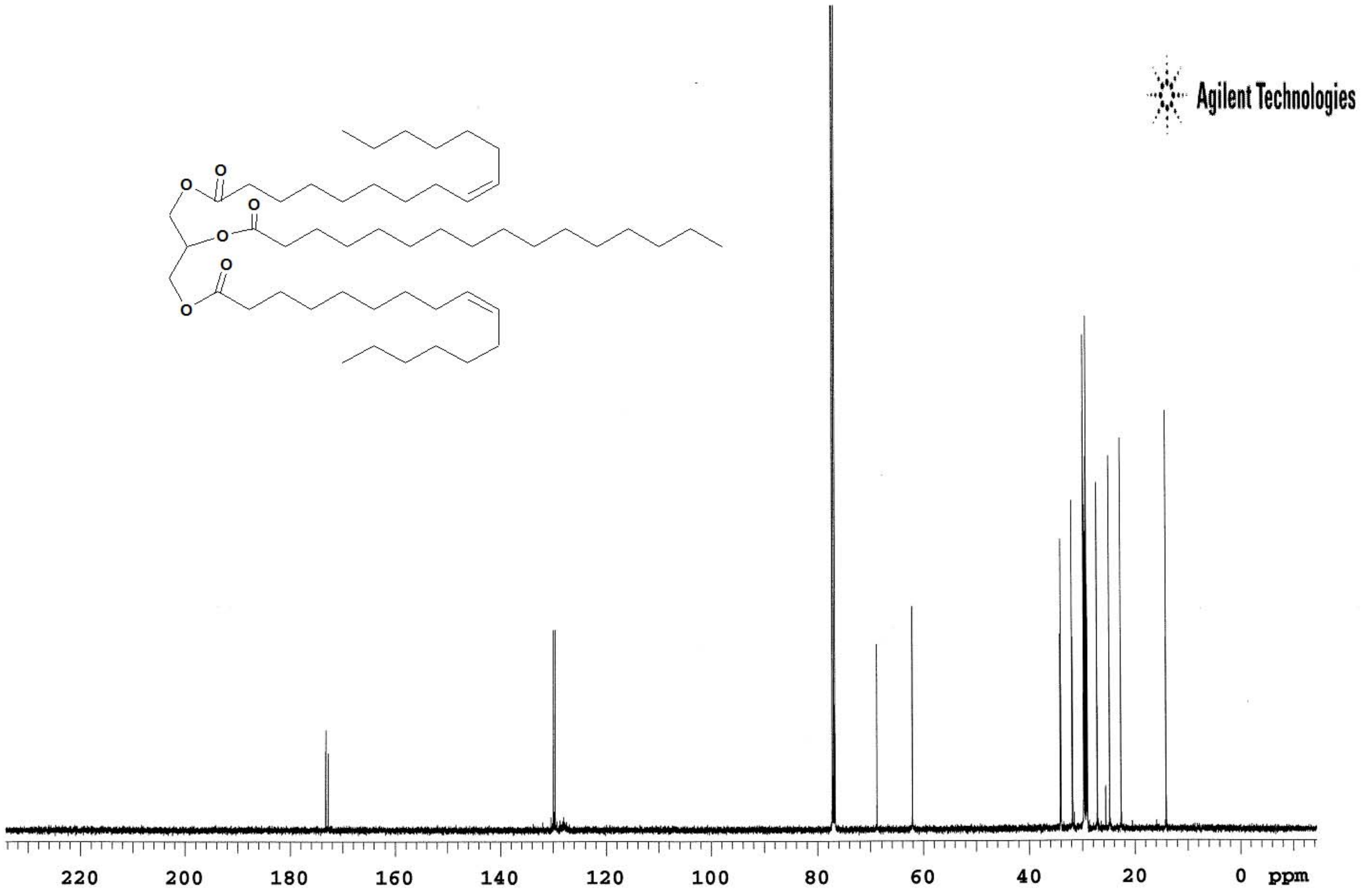

Agilent Technologies
O
220
200
180
160
140
120
100
80
60
40
20
0 ppm

Figure C

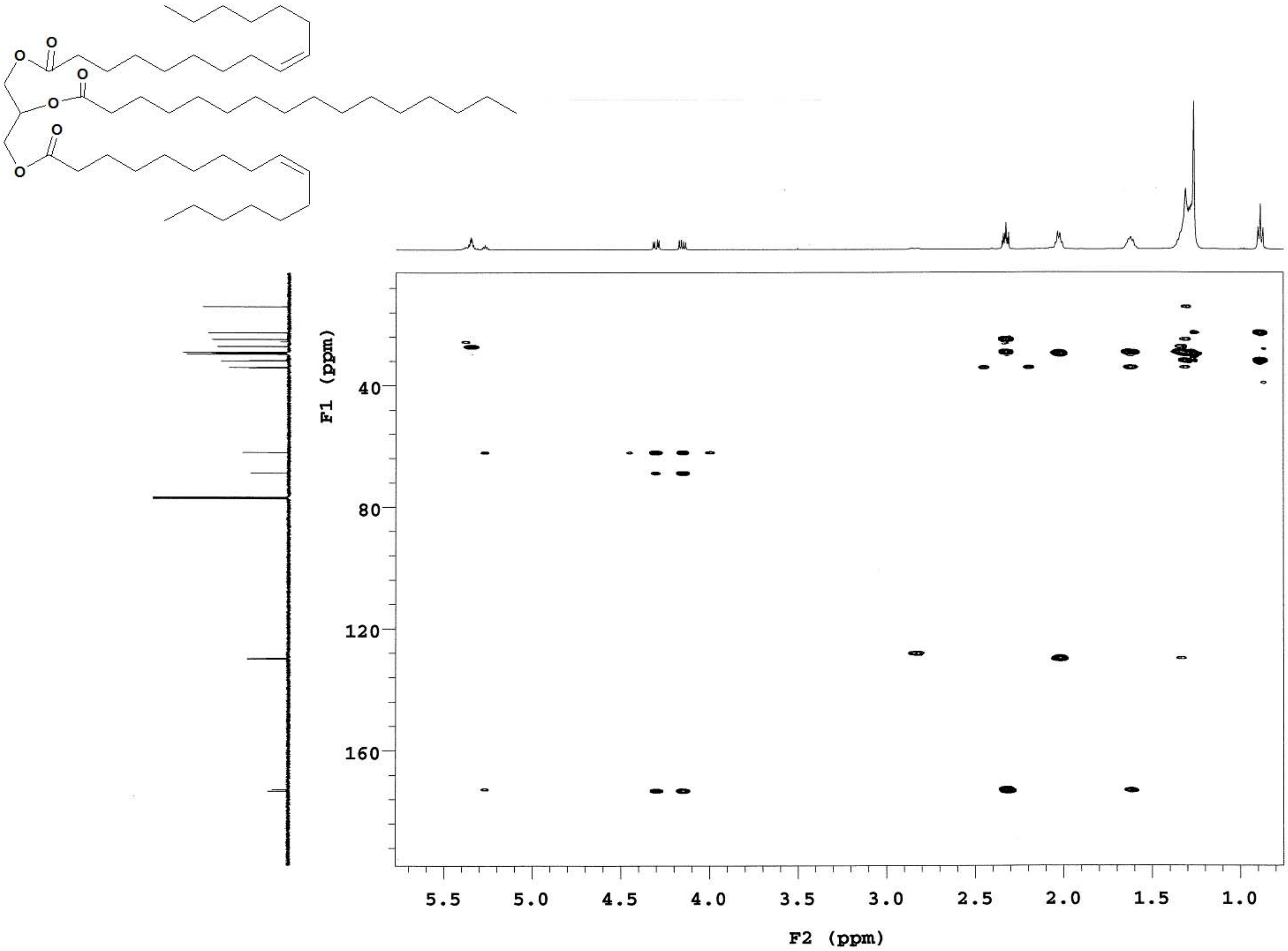

Data file /net/ormuzd/home/walkup/vnmrsys/data/amilad/AD_113_141C_20160504_01/AD_113_141C_gHMBCAD_01.fid Plot date 2016-05-05

Figure D

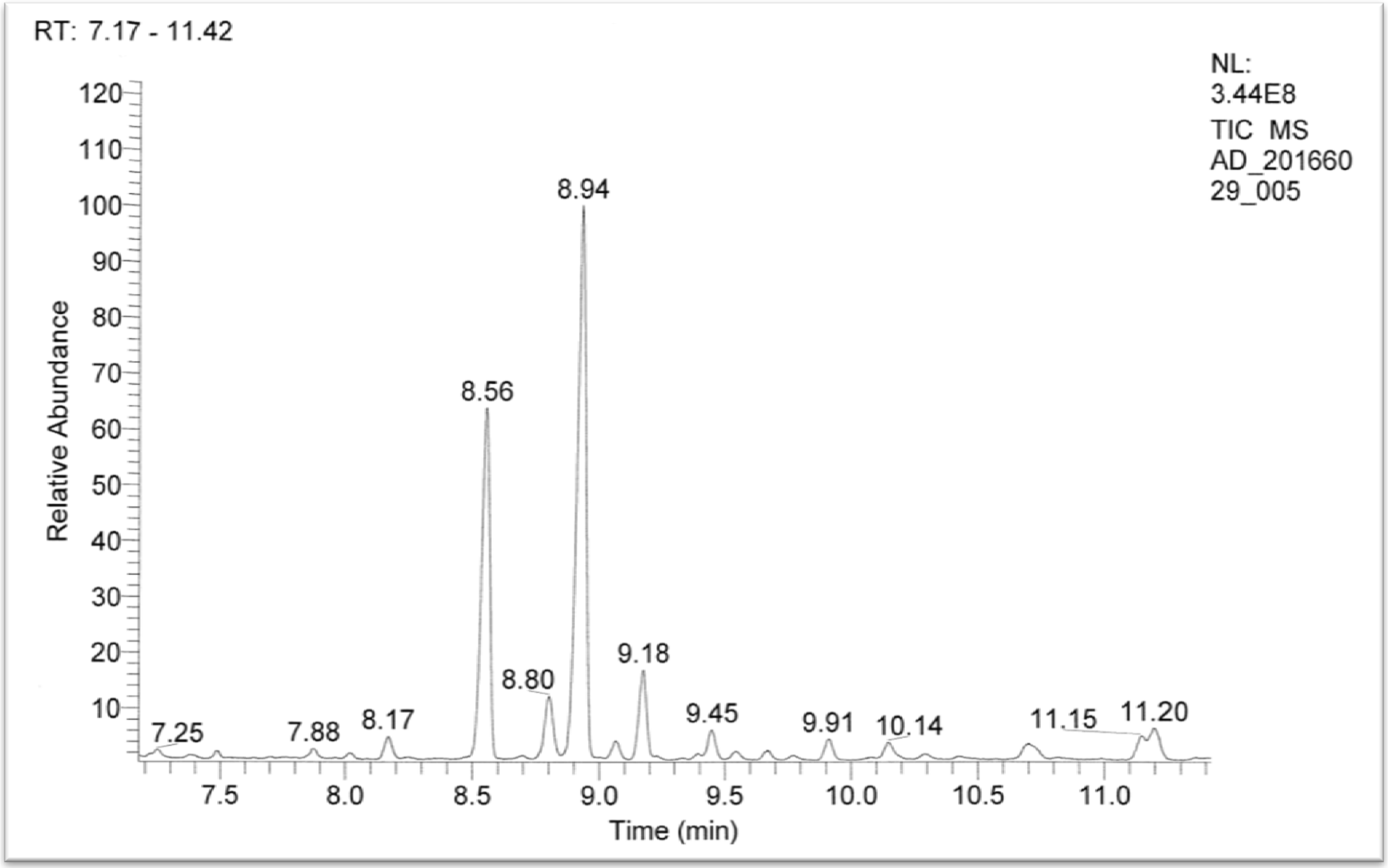
RT: 7.17 - 11.42
NL:
3.44E8
TIC MS
AD_201660
29_005
Relative Abundance
Time (min)
8.94
8.56
9.18
8.80
9.45
9.91
10.14
11.15
11.20
8.17
7.88
7.25

Figure E

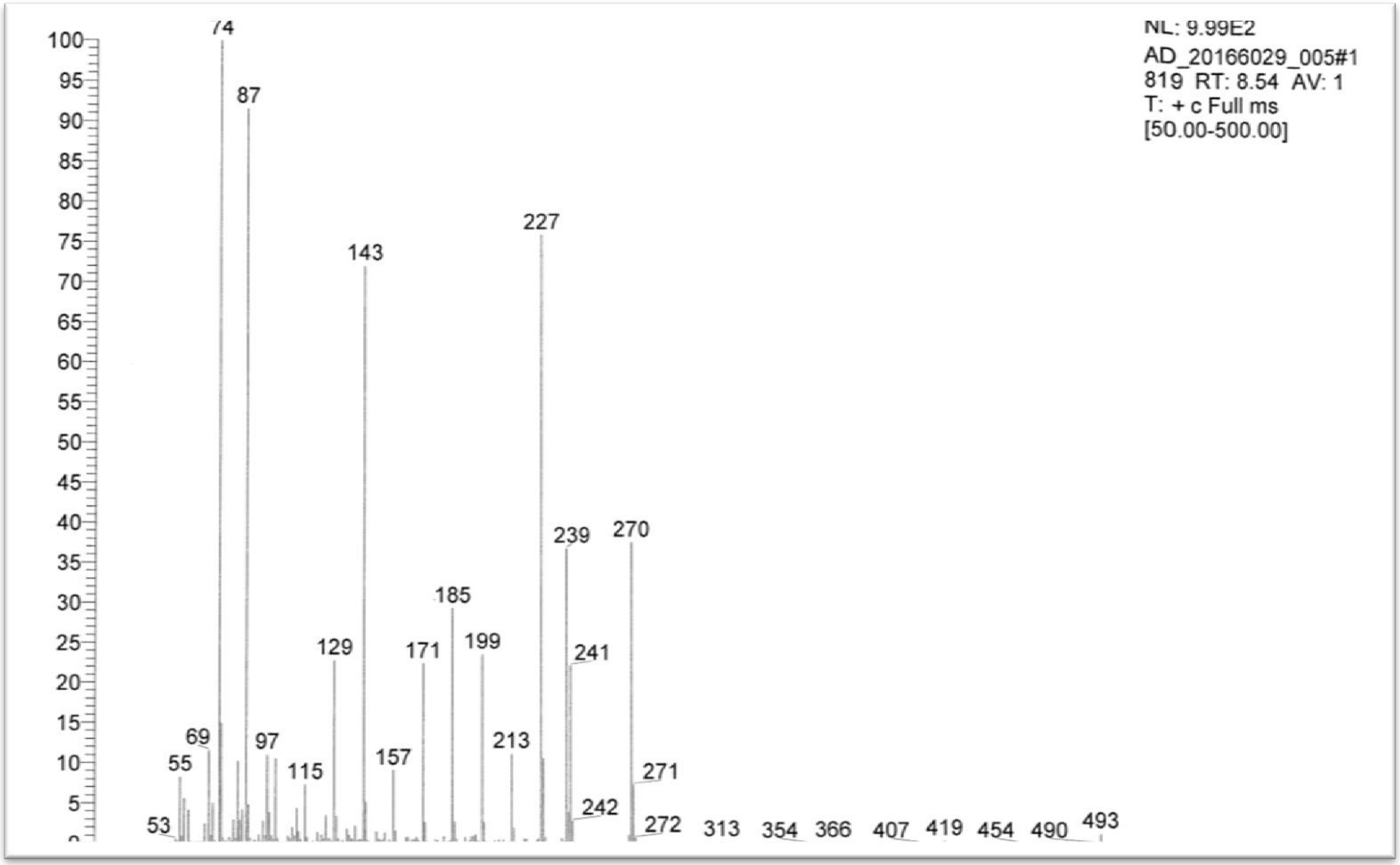

NL: 9.99E2
AD_20166029_005#1
819 RT: 8.54 AV: 1
T: + c Full ms
[50.00-500.00]
100
95
90
85
80
75
70
65
60
55
50
45
40
35
30
25
20
15
10
5
74
87
143
227
239
270
185
129
171
199
241
69
55
97
115
157
213
271
242
53
272
313
354
366
407
419
454
490
493

Figure F

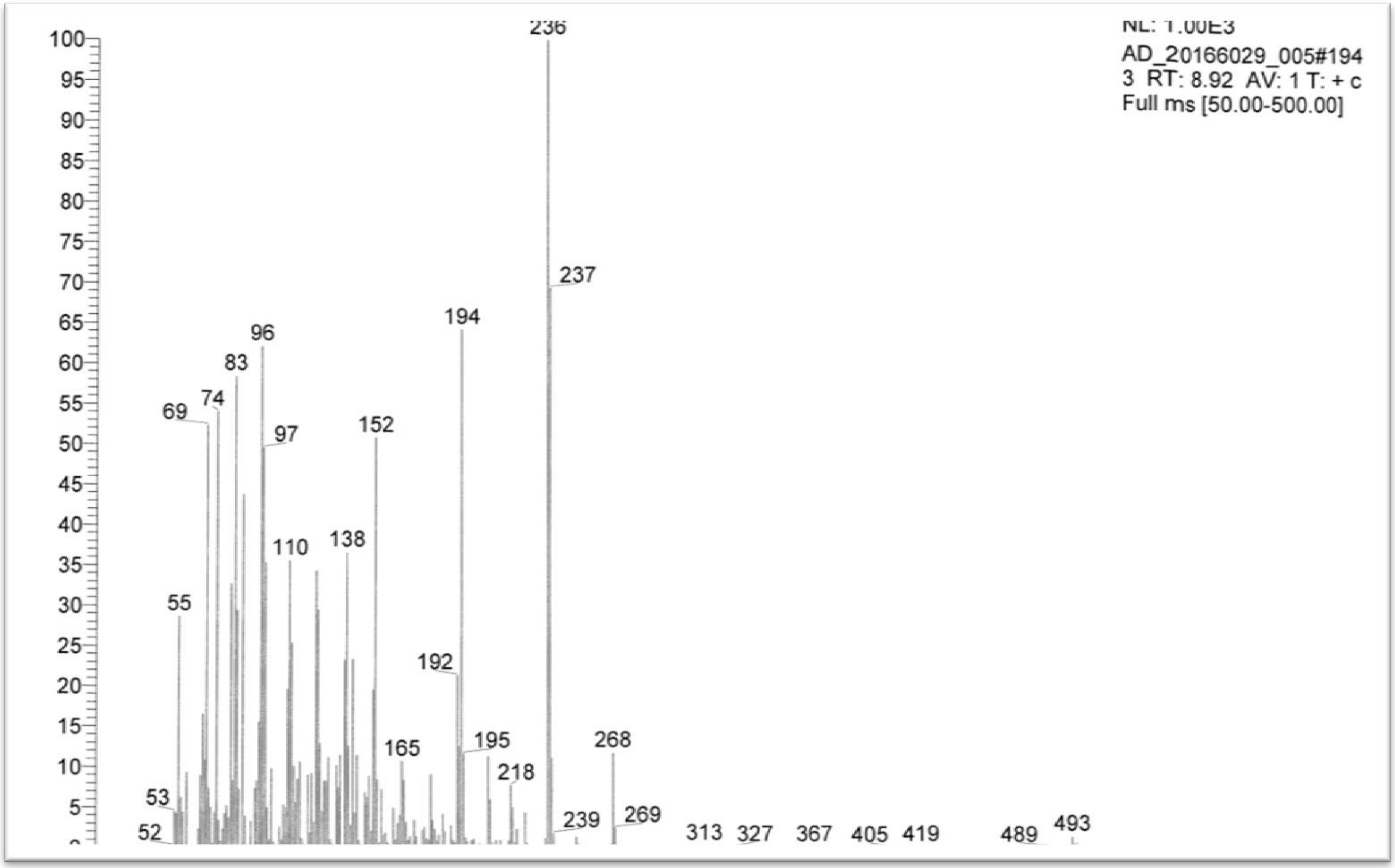
NL: 1.00E3
AD_20166029_005#194
3 RT: 8.92 AV: 1 T: + c
Full ms [50.00-500.00]
100
95
90
85
80
75
70
65
60
55
50
45
40
35
30
25
20
15
10
5
236
237
194
96
83
74
69
97
152
110
138
55
192
165
195
218
268
53
52
239
269
313
327
367
405
419
489
493

Figure G

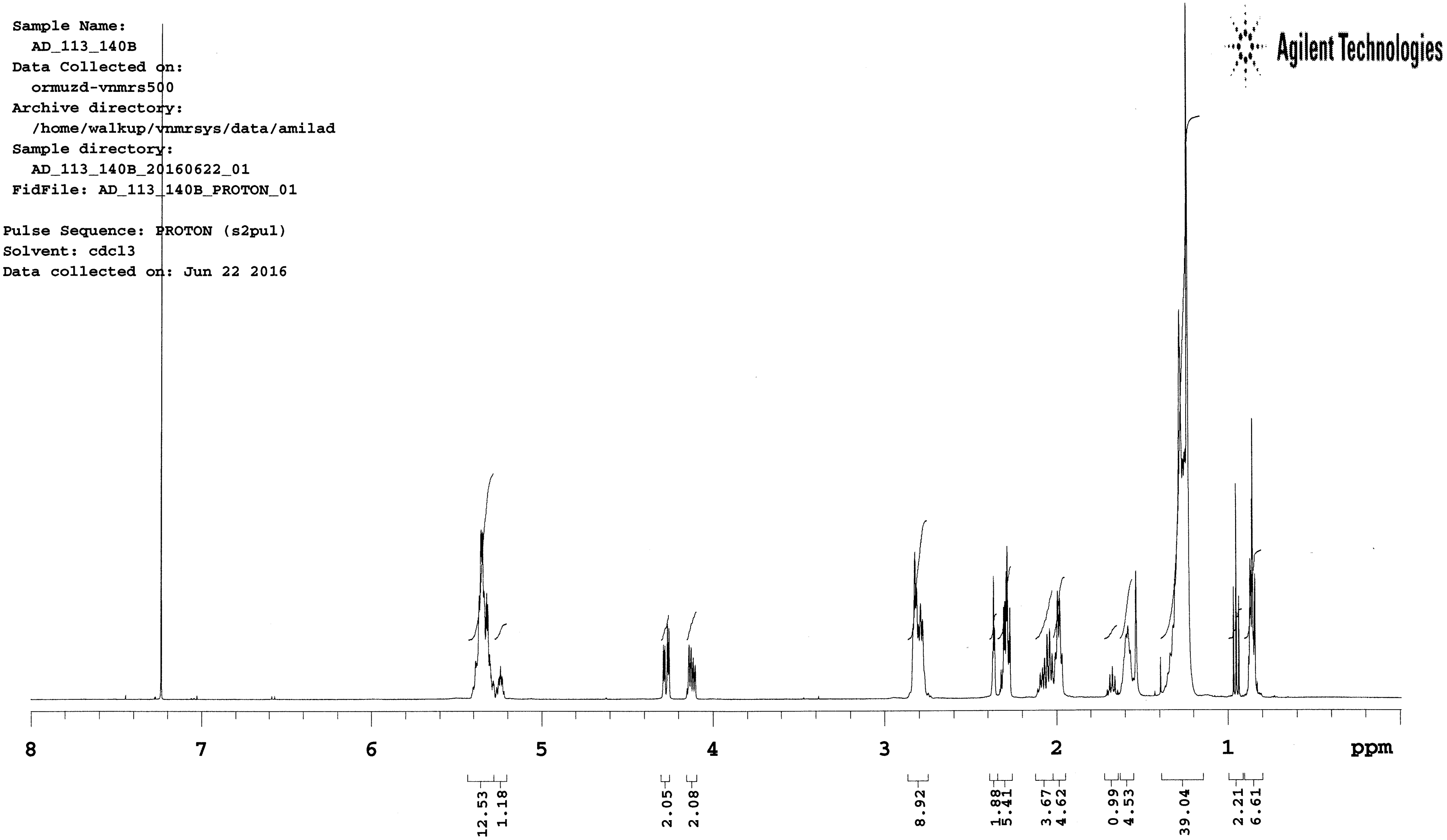
Sample Name:
AD_113_140B
Data Collected on:
ormuzd-vnmrs500
Archive directory:
/home/walkup/vnmrsys/data/amilad
Sample directory:
AD_113_140B_20160622_01
FidFile: AD_113_140B_PROTON_01
Pulse Sequence: PROTON (s2pul)
Solvent: cdcl3
Data collected on: Jun 22 2016
Agilent Technologies
8
7
6
5
4
3
2
1
ppm
12.53
1.18
2.05
2.08
8.92
1.88
5.41
3.67
4.62
0.99
4.53
39.04
2.21
6.61

Figure H

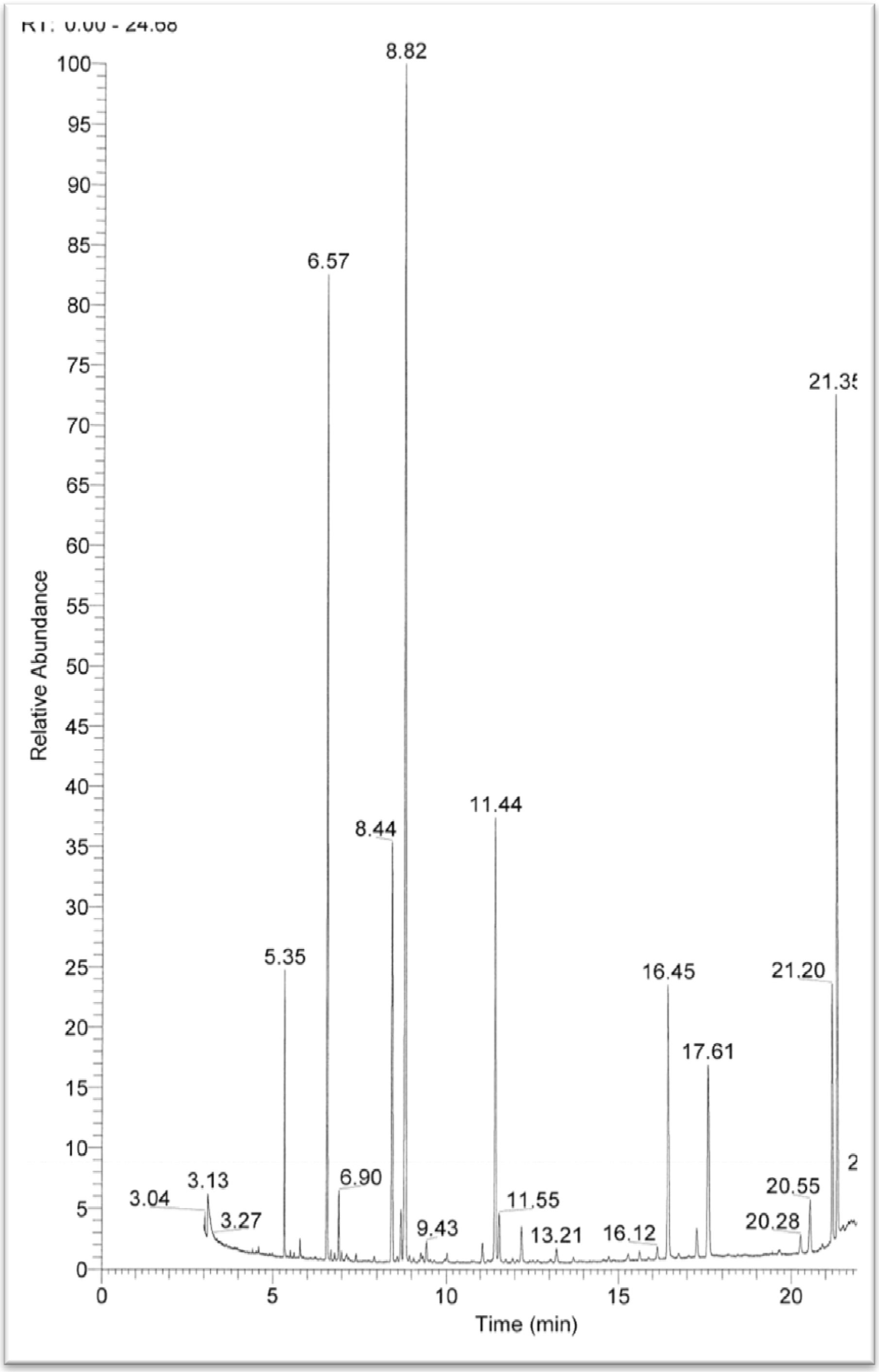

RT: 0.00 - 24.68
8.82
6.57
21.35
11.44
8.44
5.35
16.45
21.20
17.61
3.13
3.04
3.27
6.90
11.55
9.43
13.21
16.12
20.55
20.28
Relative Abundance
Time (min)

Figure I

INDEX	FREQUENCY	PPM	HEIGHT
1	4035.5	8.076	107.1
2	3617.8	7.240	51.4
3	2127.1	4.257	16.4
4	2121.3	4.245	35.7
5	2115.4	4.233	16.8
6	1821.0	3.644	11.0
7	855.6	1.712	8.0
8	729.4	1.460	8.5
9	726.9	1.455	8.2
10	722.1	1.445	10.4
11	720.1	1.441	10.5
12	715.7	1.432	8.0
13	712.8	1.426	9.0
14	693.2	1.387	8.0
15	687.8	1.376	11.0
16	663.9	1.329	10.6
17	660.4	1.322	14.7
18	656.5	1.314	22.0
19	652.6	1.306	21.2
20	649.2	1.299	14.7
21	614.9	1.231	30.0
22	472.6	0.946	33.5
23	465.3	0.931	69.7
24	458.0	0.916	26.8
25	448.7	0.898	16.2
26	441.8	0.884	35.3
27	437.4	0.875	8.8
28	434.5	0.869	14.7

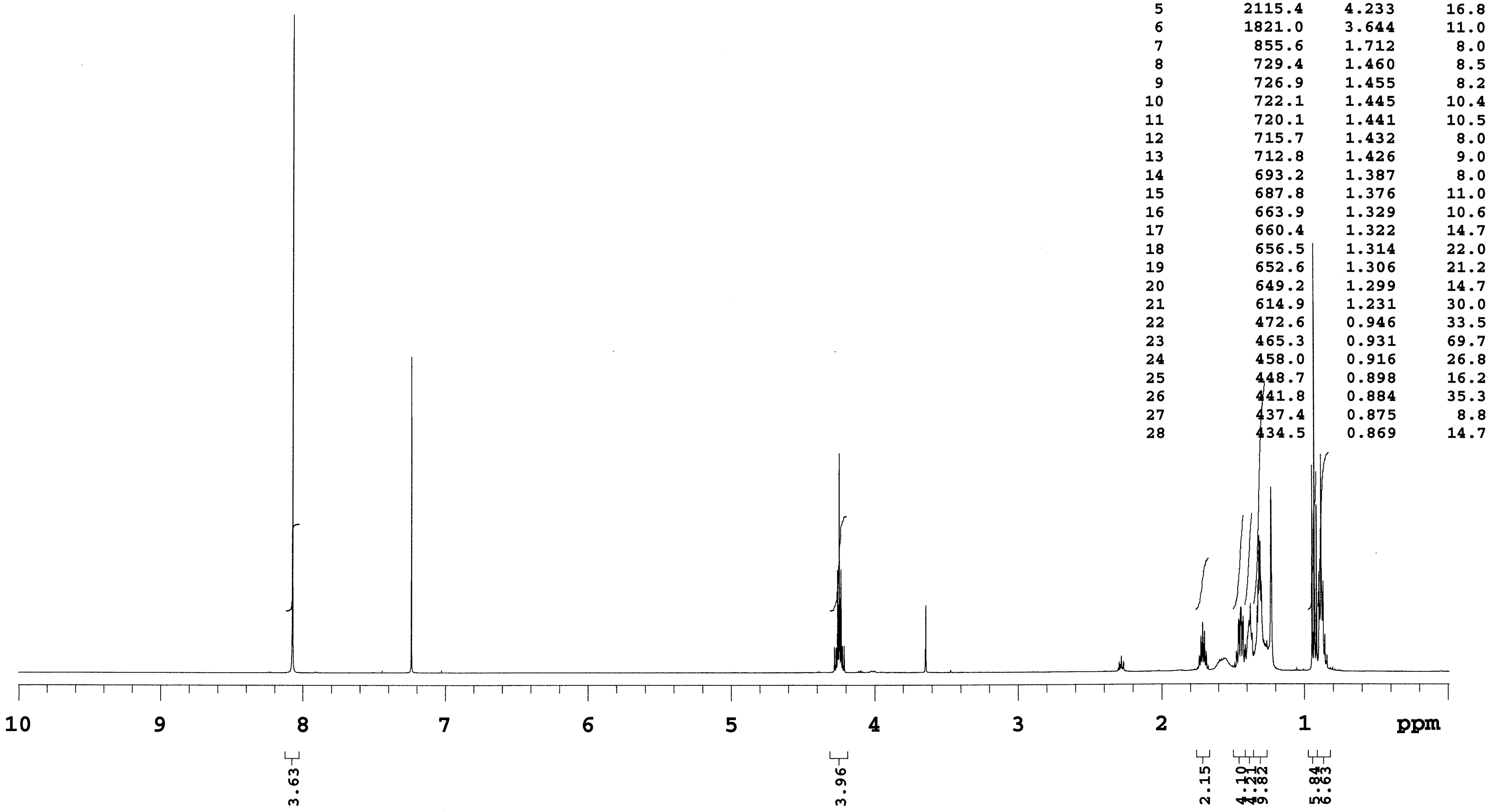

Figure J

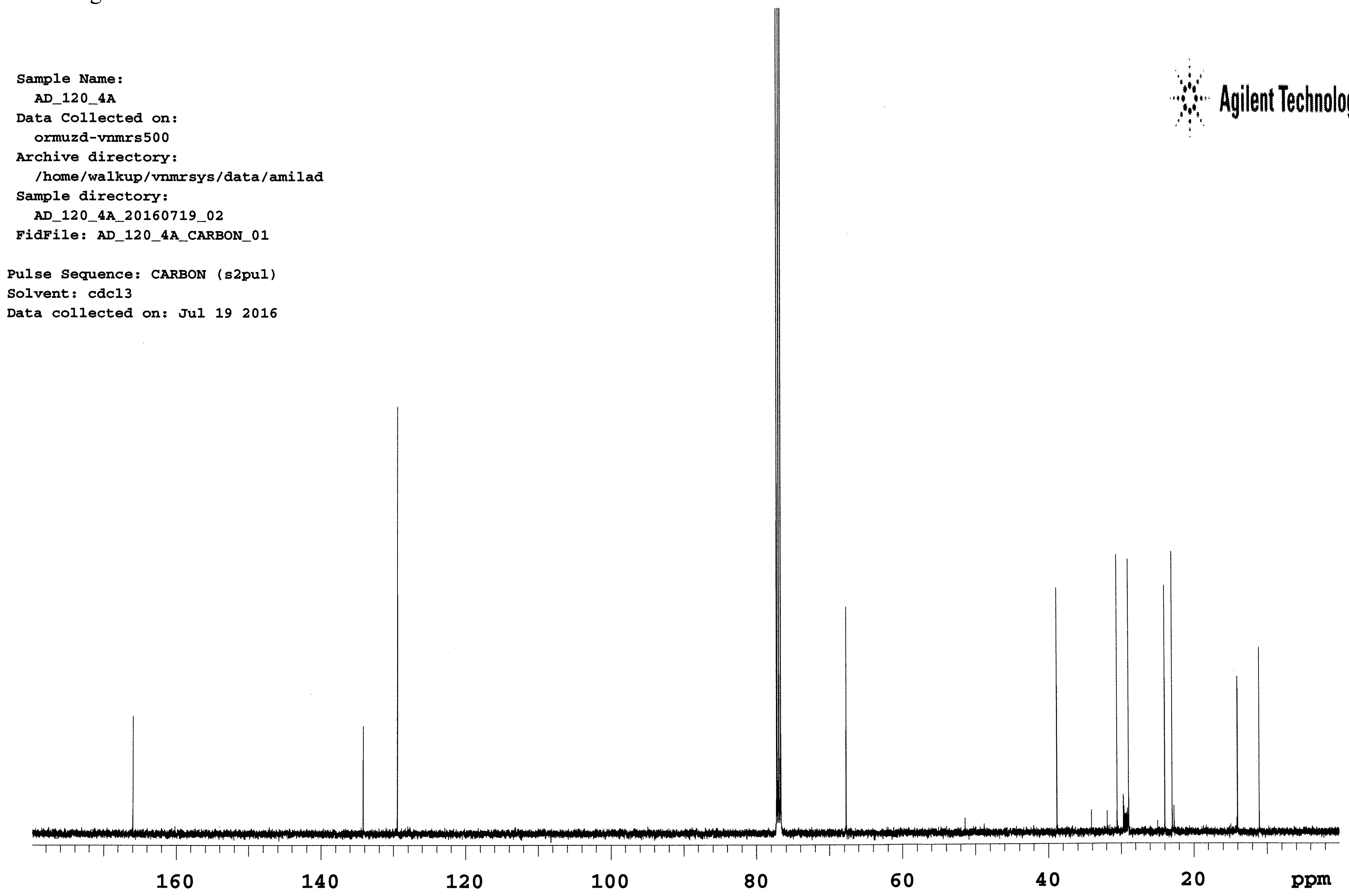

Sample Name:
AD_120_4A
Data Collected on:
ormuzd-vnmrs500
Archive directory:
/home/walkup/vnmrsys/data/amilad
Sample directory:
AD_120_4A_20160719_02
FidFile: AD_120_4A_CARBON_01
Pulse Sequence: CARBON (s2pul)
Solvent: cdcl3
Data collected on: Jul 19 2016
Agilent Technologies
160
140
120
100
80
60
40
20
ppm

Figure K

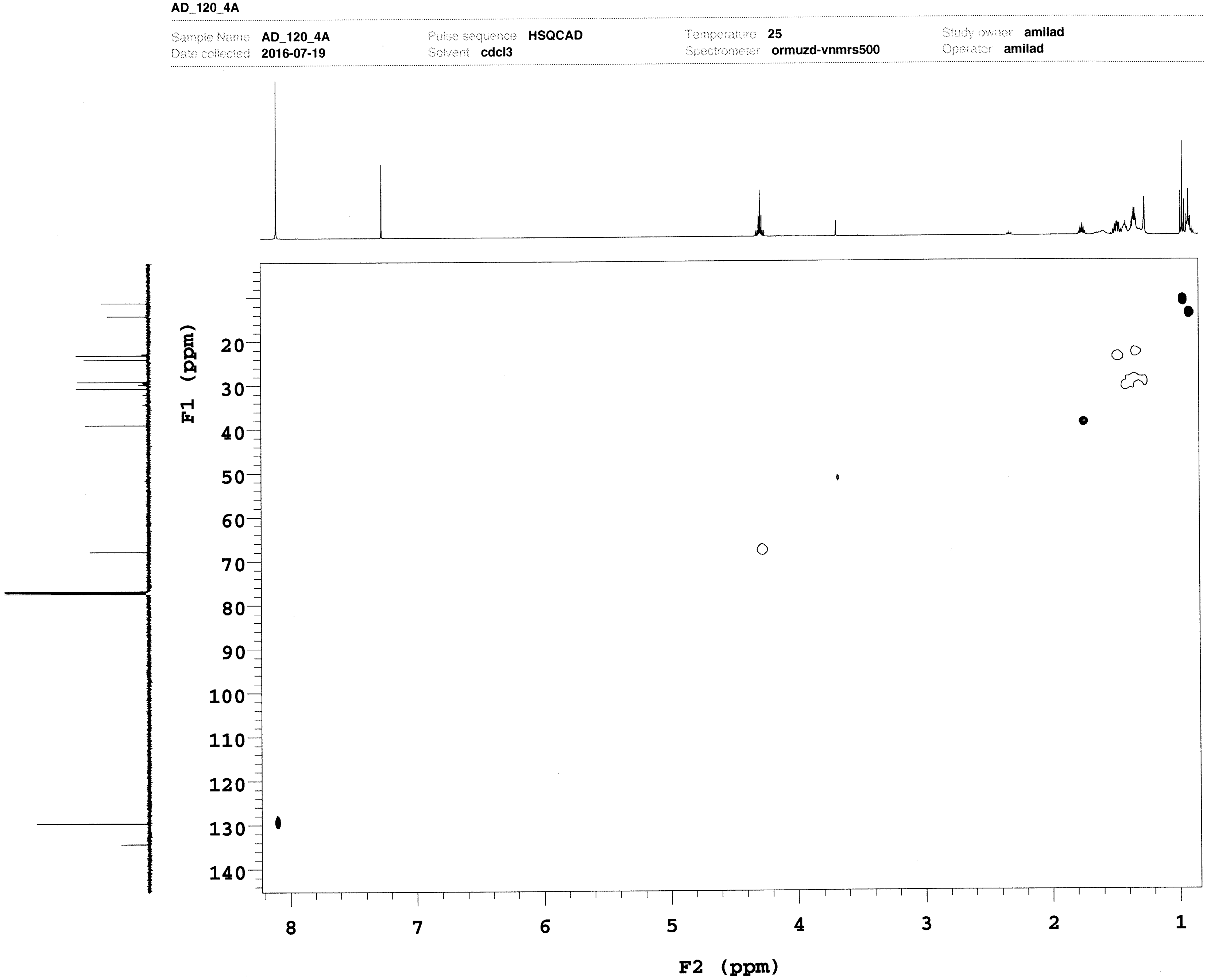

Data file /net/ormuzd/home/walkup/vnmrsys/data/amilad/AD_120_4A_20160719_02/AD_120_4A_HSQCAD_01.fid

Plot date 2016-07-20

Figure L

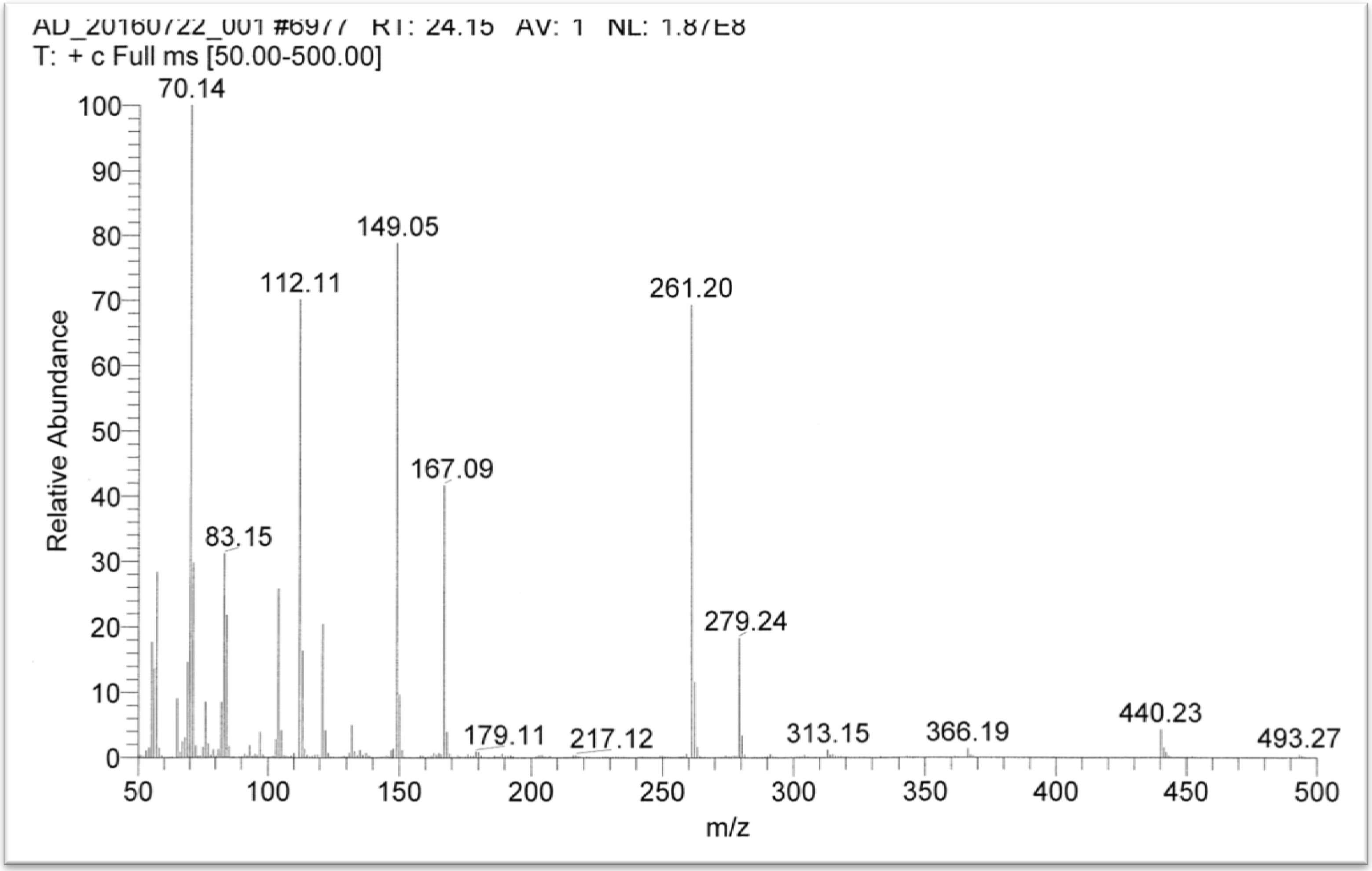

AD_20160722_001 #6977 RT: 24.15 AV: 1 NL: 1.87E8
T: + c Full ms [50.00-500.00]
70.14
149.05
112.11
261.20
167.09
83.15
279.24
179.11
217.12
313.15
366.19
440.23
493.27
Relative Abundance
m/z

Figure M

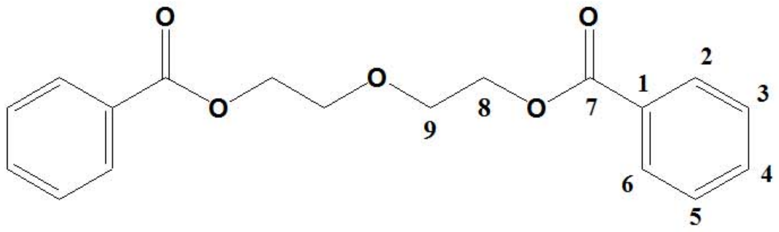

INDEX	FREQUENCY	PPM	HEIGHT
1	4010.5	8.026	71.8
2	4009.5	8.024	78.7
3	4004.2	8.013	22.8
4	4002.2	8.009	90.8
5	4000.7	8.006	67.5
6	3768.4	7.541	12.7
7	3767.0	7.539	21.1
8	3766.0	7.537	12.0
9	3759.6	7.524	55.5
10	3753.8	7.512	16.5
11	3752.3	7.509	35.9
12	3750.8	7.506	17.4
13	3695.1	7.395	72.5
14	3687.2	7.379	96.8
15	3681.4	7.367	18.4
16	3679.4	7.363	54.7
17	3617.8	7.240	34.6
18	2244.5	4.492	86.1
19	2240.1	4.483	99.2
20	2238.6	4.480	54.9
21	2235.2	4.473	107.1
22	1937.9	3.878	105.2
23	1934.4	3.871	54.2
24	1933.0	3.868	105.1
25	1928.1	3.859	89.5

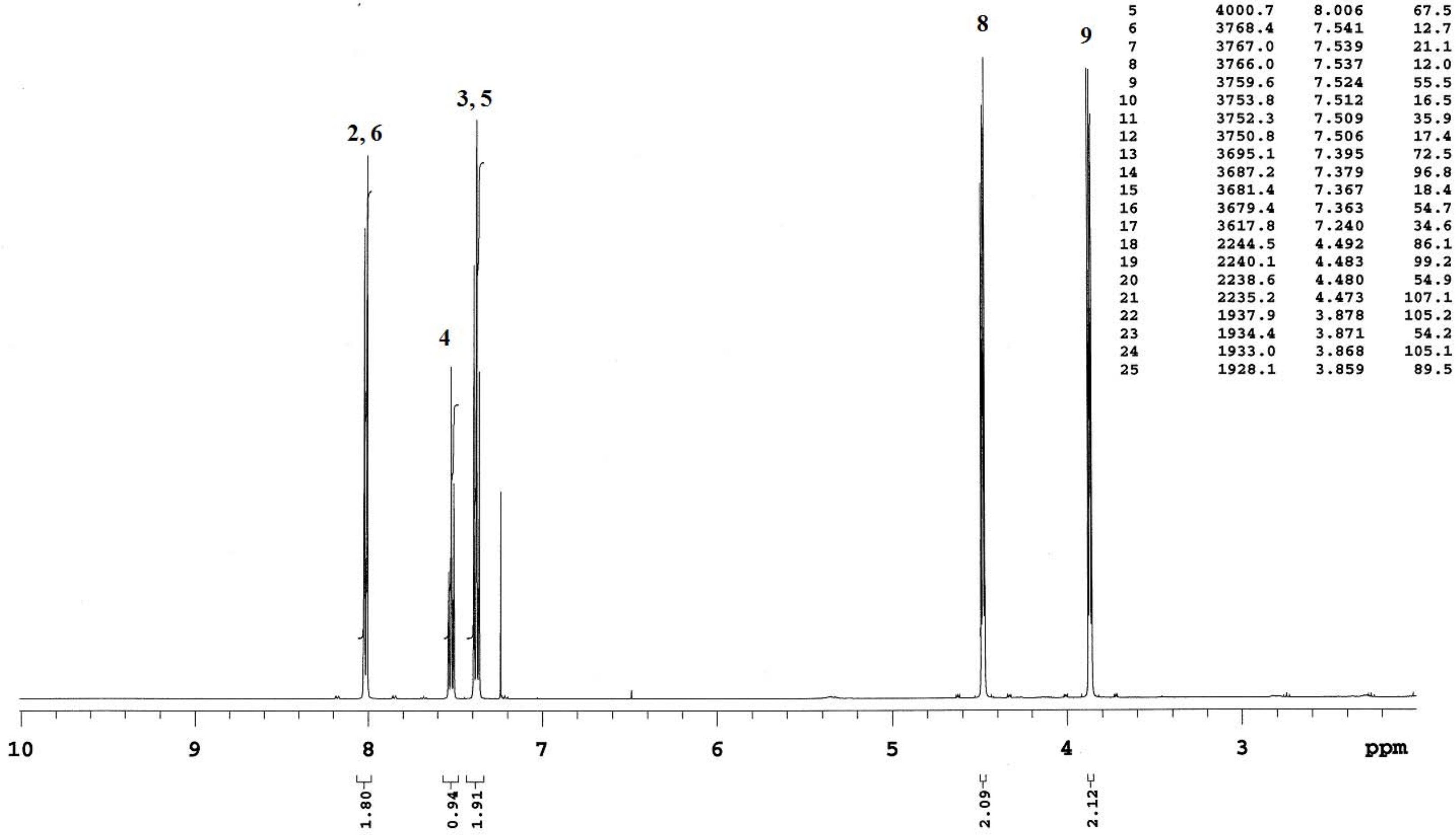

Figure N

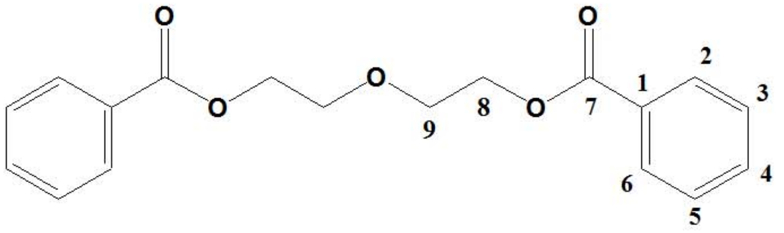

INDEX	FREQUENCY	PPM	HEIGHT
1	20921.6	166.509	14.5
2	16706.4	132.961	61.3
3	16329.7	129.963	19.0
4	16287.7	129.629	116.3
5	16120.8	128.301	117.9
6	9706.4	77.250	38.5
7	9674.9	77.000	34.4
8	9642.5	76.742	39.1
9	8690.8	69.167	35.7
10	8037.5	63.968	56.3

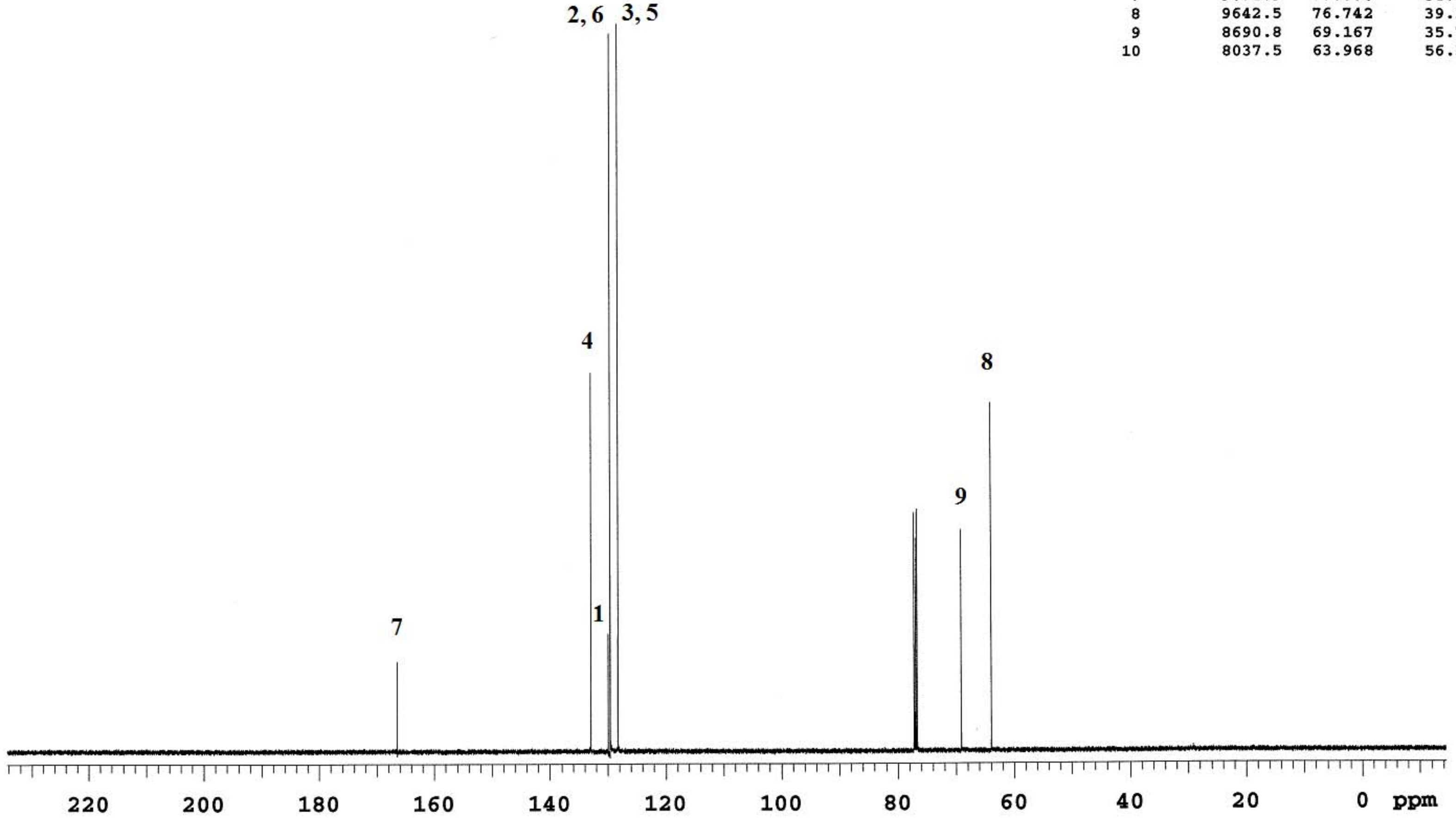

Figure O

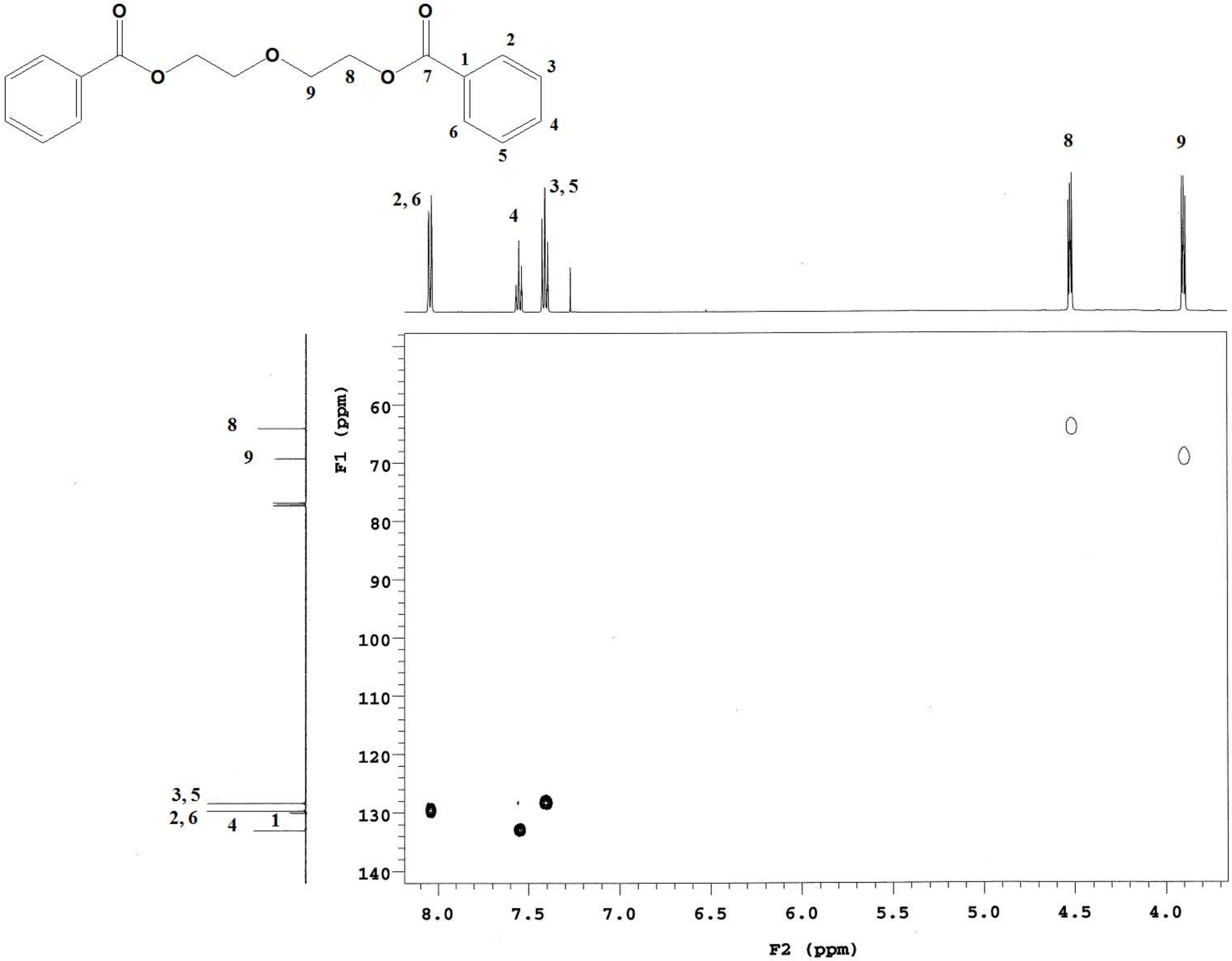

9
8
7
1
2
3
4
5
6
2, 6
4
3, 5
8
9
F1 (ppm)
60
70
80
90
100
110
120
130
140
3, 5
2, 6
4
1
8.0
7.5
7.0
6.5
6.0
5.5
5.0
4.5
4.0
F2 (ppm)

Figure P

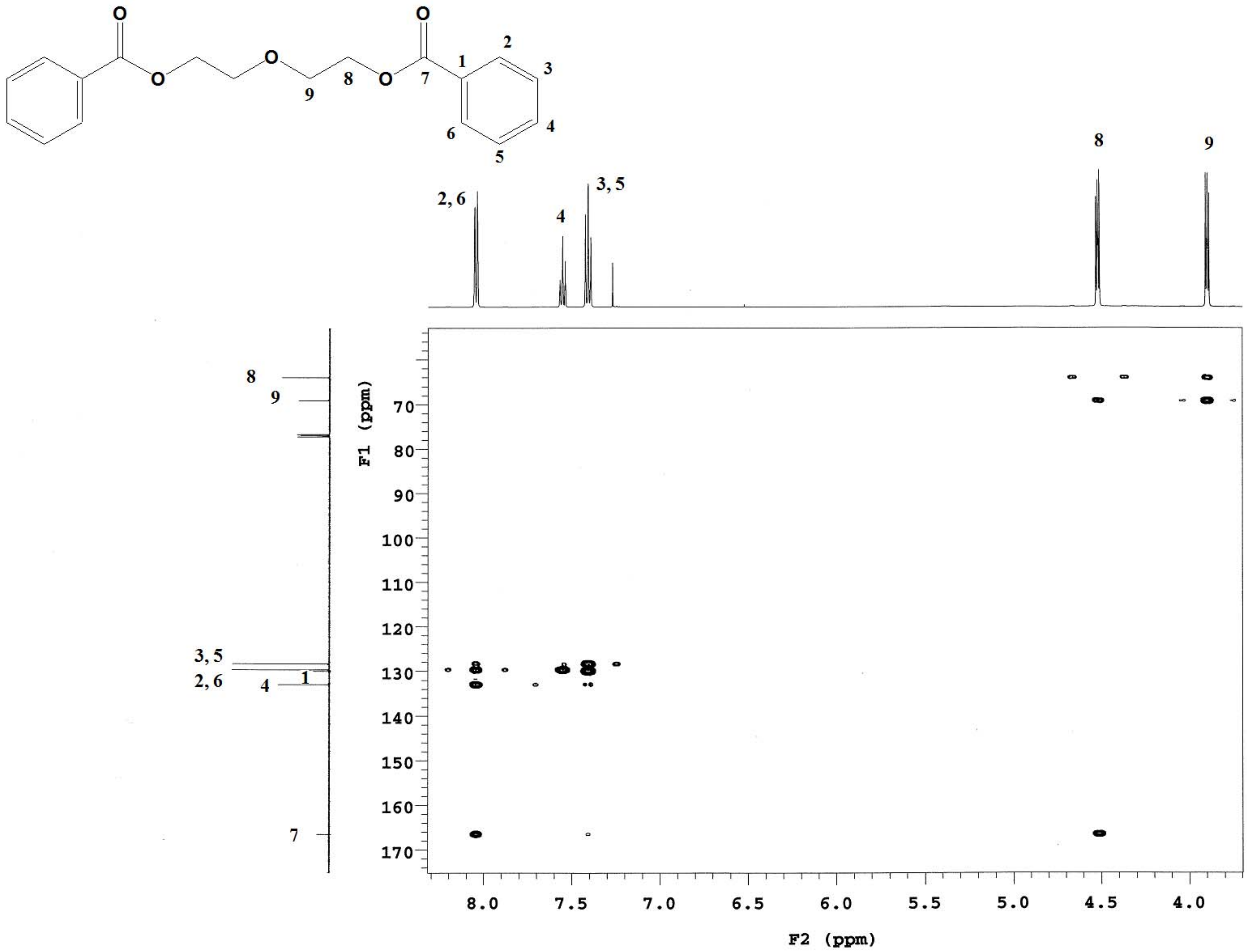

O
O
O
O
O
1
2
3
4
5
6
7
8
9
2, 6
4
3, 5
8
9
8
9
3, 5
2, 6
1
4
7
F1 (ppm)
70
80
90
100
110
120
130
140
150
160
170
8.0
7.5
7.0
6.5
6.0
5.5
5.0
4.5
4.0
F2 (ppm)

Figure Q

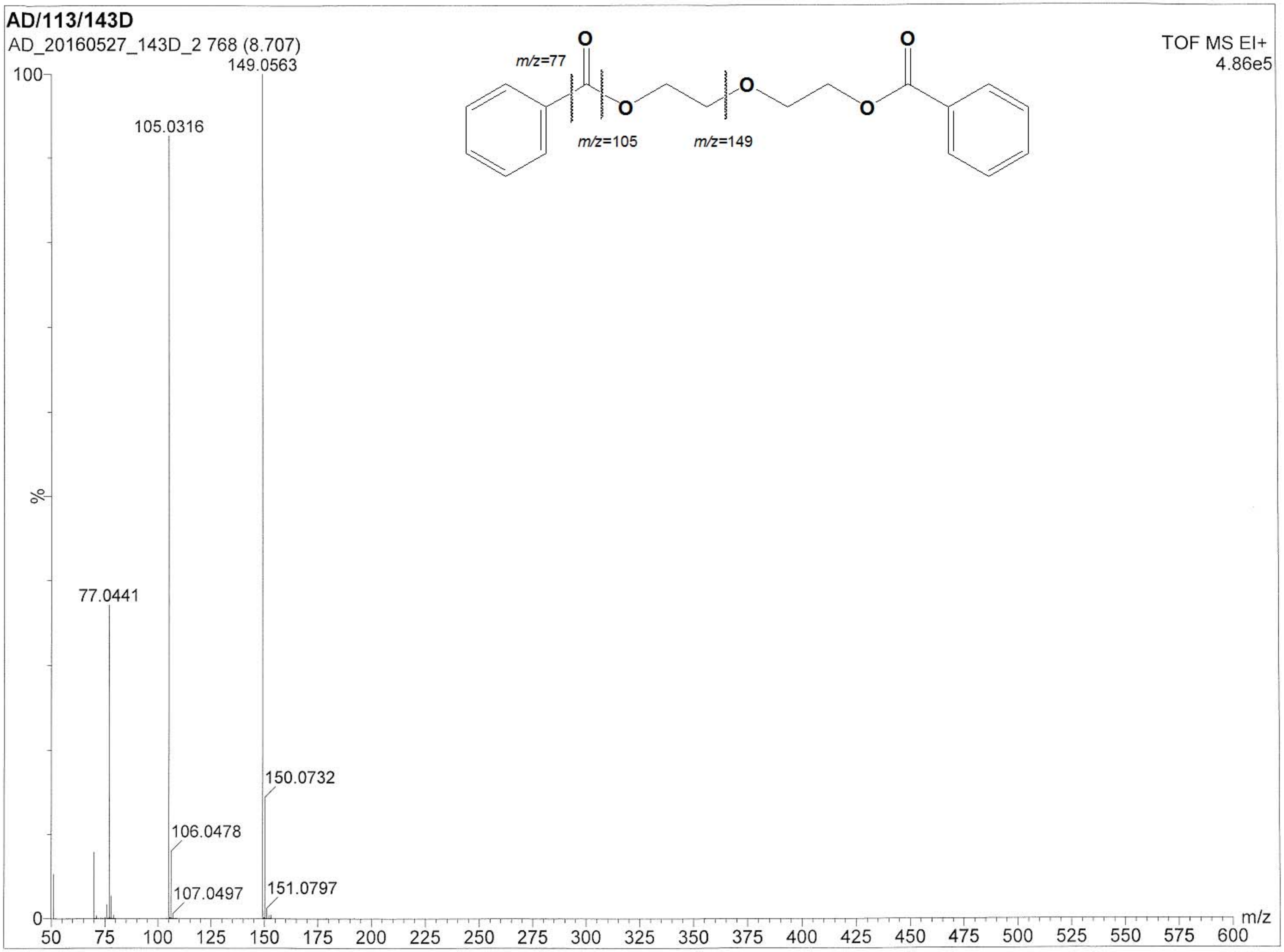

AD/113/143D
AD_20160527_143D_2 768 (8.707)
TOF MS EI+
4.86e5
149.0563
105.0316
77.0441
150.0732
106.0478
107.0497
151.0797
m/z=77
m/z=105
m/z=149
O
O
O
O
O
100
%
0
50
75
100
125
150
175
200
225
250
275
300
325
350
375
400
425
450
475
500
525
550
575
600
m/z

Figure R

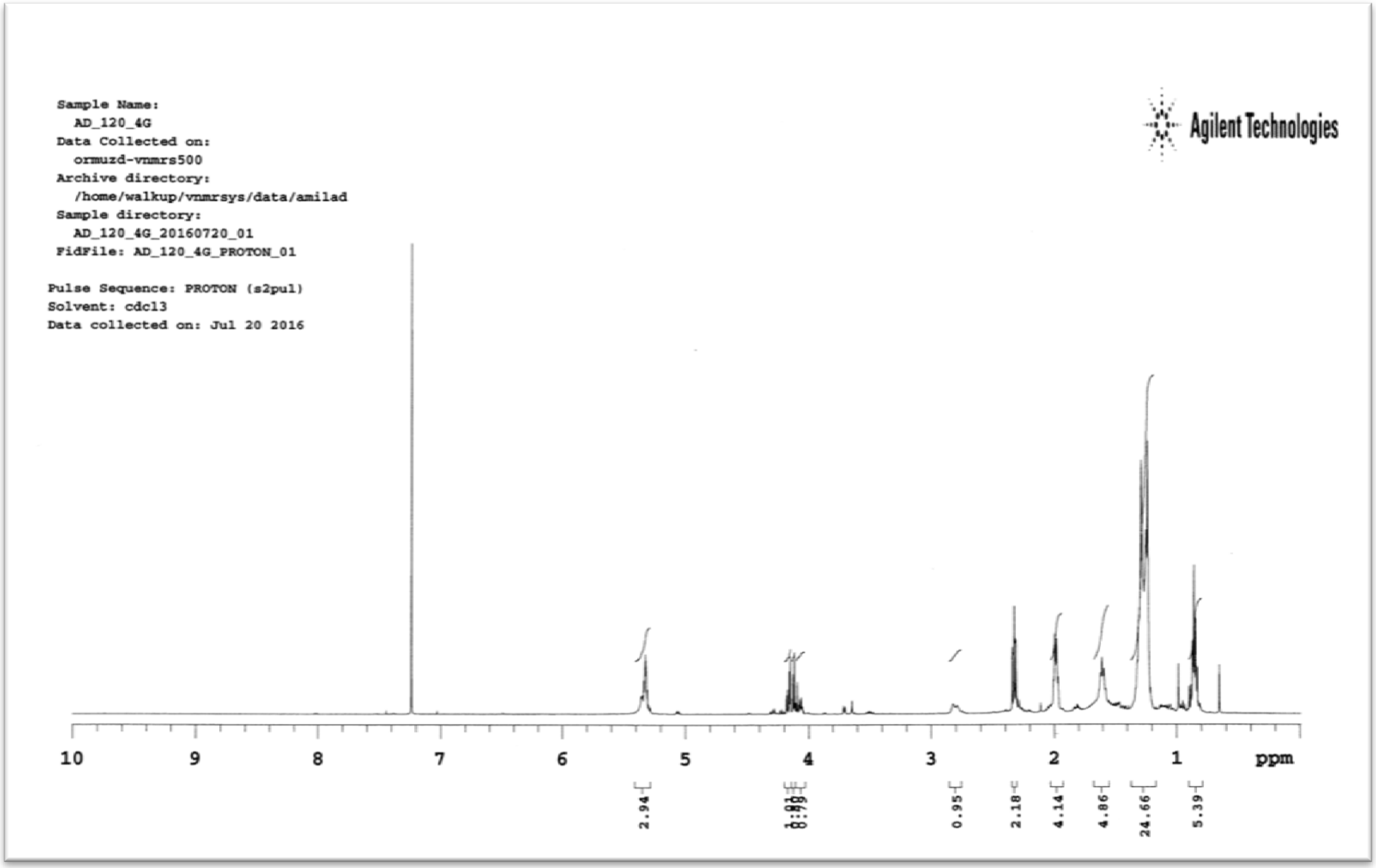

Sample Name:
AD_120_4G
Data Collected on:
ormuzd-vnmrs500
Archive directory:
/home/walkup/vnmrsys/data/amilad
Sample directory:
AD_120_4G_20160720_01
FidFile: AD_120_4G_PROTON_01
Pulse Sequence: PROTON (s2pul)
Solvent: cdcl3
Data collected on: Jul 20 2016
Agilent Technologies
10
9
8
7
6
5
4
3
2
1
ppm
2.94
0.95
2.18
4.14
4.86
24.66
5.39

Figure S

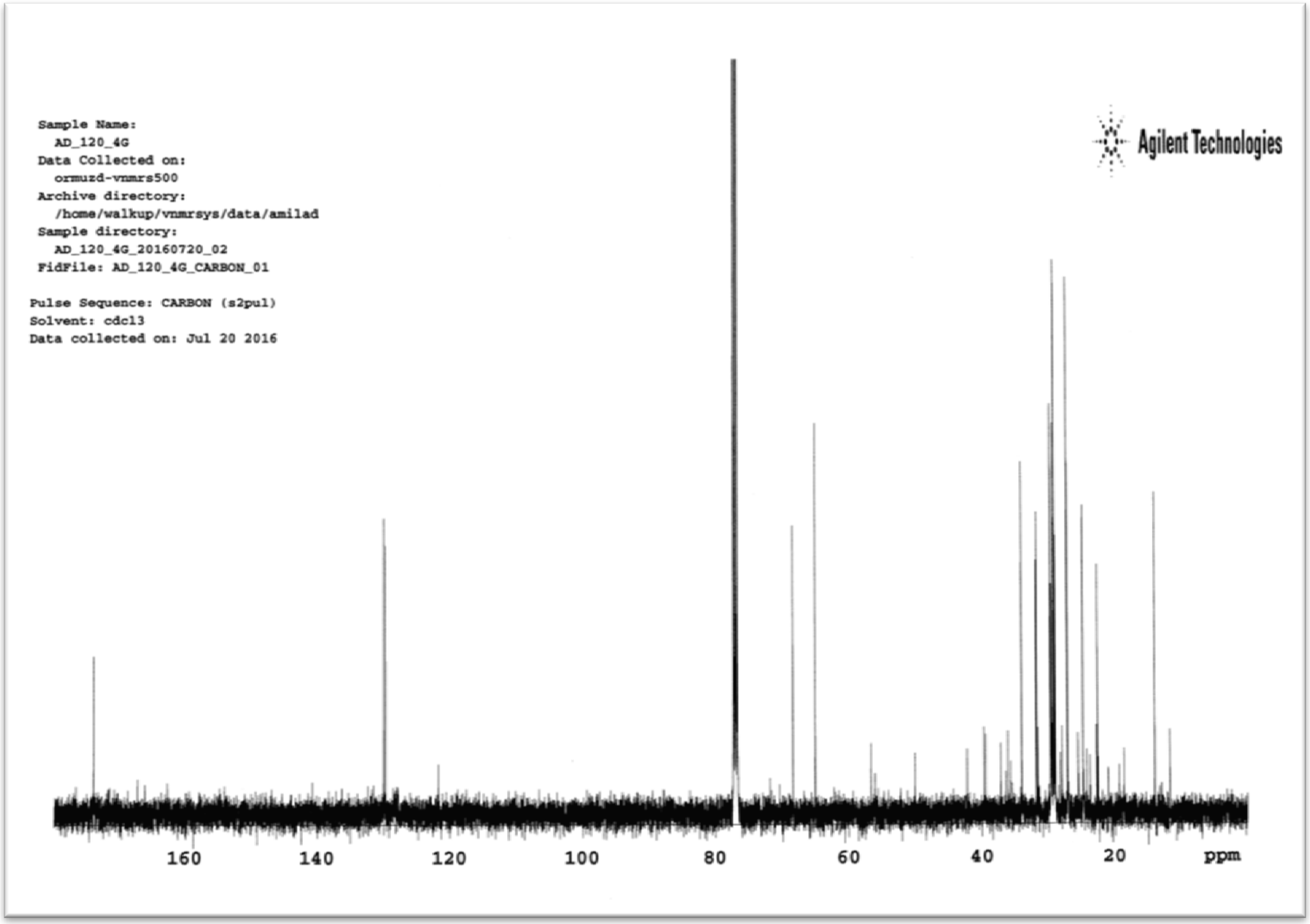

Sample Name:
AD_120_4G
Data Collected on:
ormuzd-vnmrs500
Archive directory:
/home/walkup/vnmrsys/data/amilad
Sample directory:
AD_120_4G_20160720_02
FidFile: AD_120_4G_CARBON_01
Pulse Sequence: CARBON (s2pul)
Solvent: cdcl3
Data collected on: Jul 20 2016
Agilent Technologies
160
140
120
100
80
60
40
20
ppm

Figure T

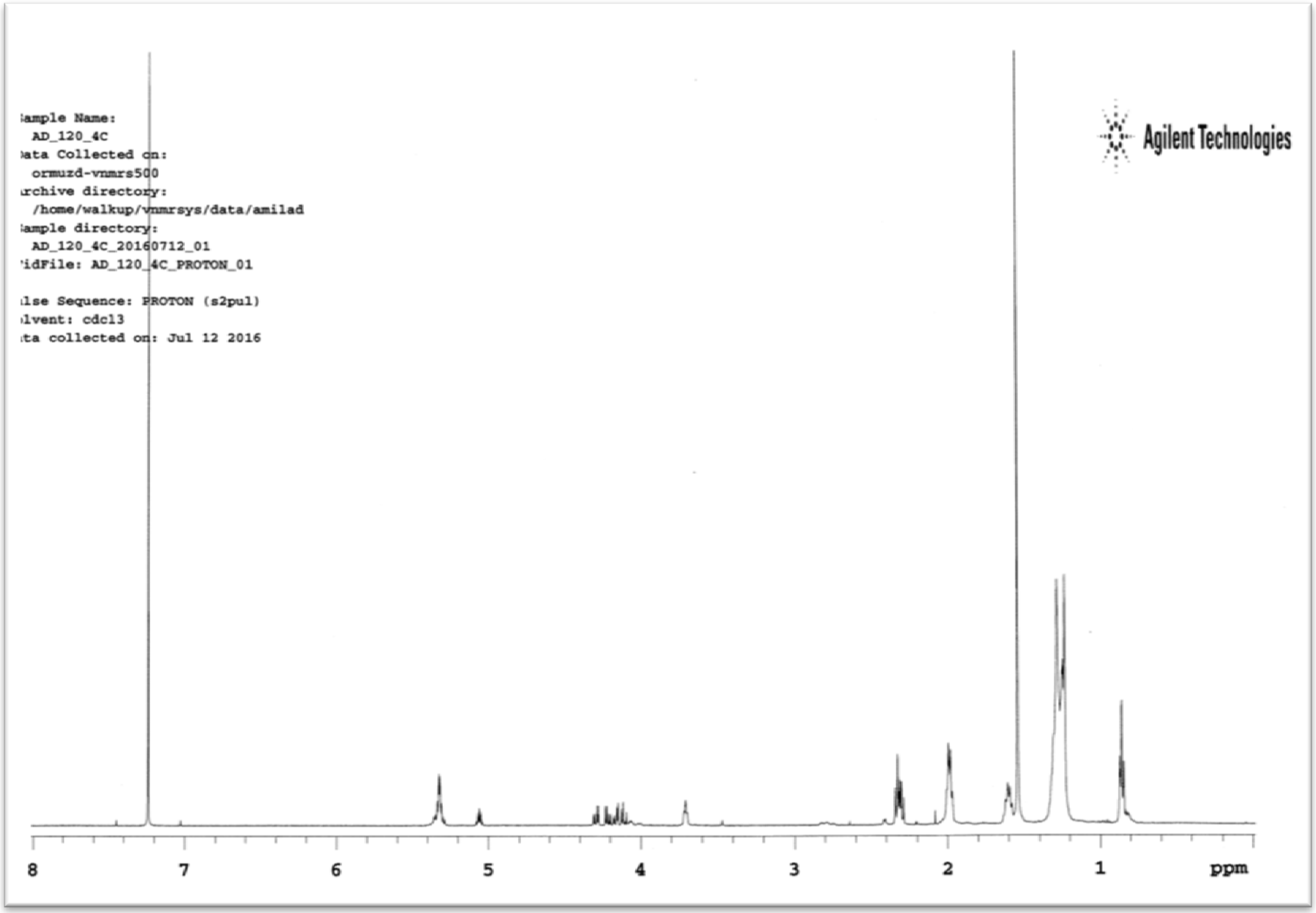

ample Name:
AD_120_4C
ata Collected on:
ormuzd-vnmrs500
rchive directory:
/home/walkup/vnmrsys/data/amilad
ample directory:
AD_120_4C_20160712_01
idFile: AD_120_4C_PROTON_01
lse Sequence: PROTON (s2pul)
lvent: cdcl3
ta collected on: Jul 12 2016
Agilent Technologies
8
7
6
5
4
3
2
1
ppm

Figure U

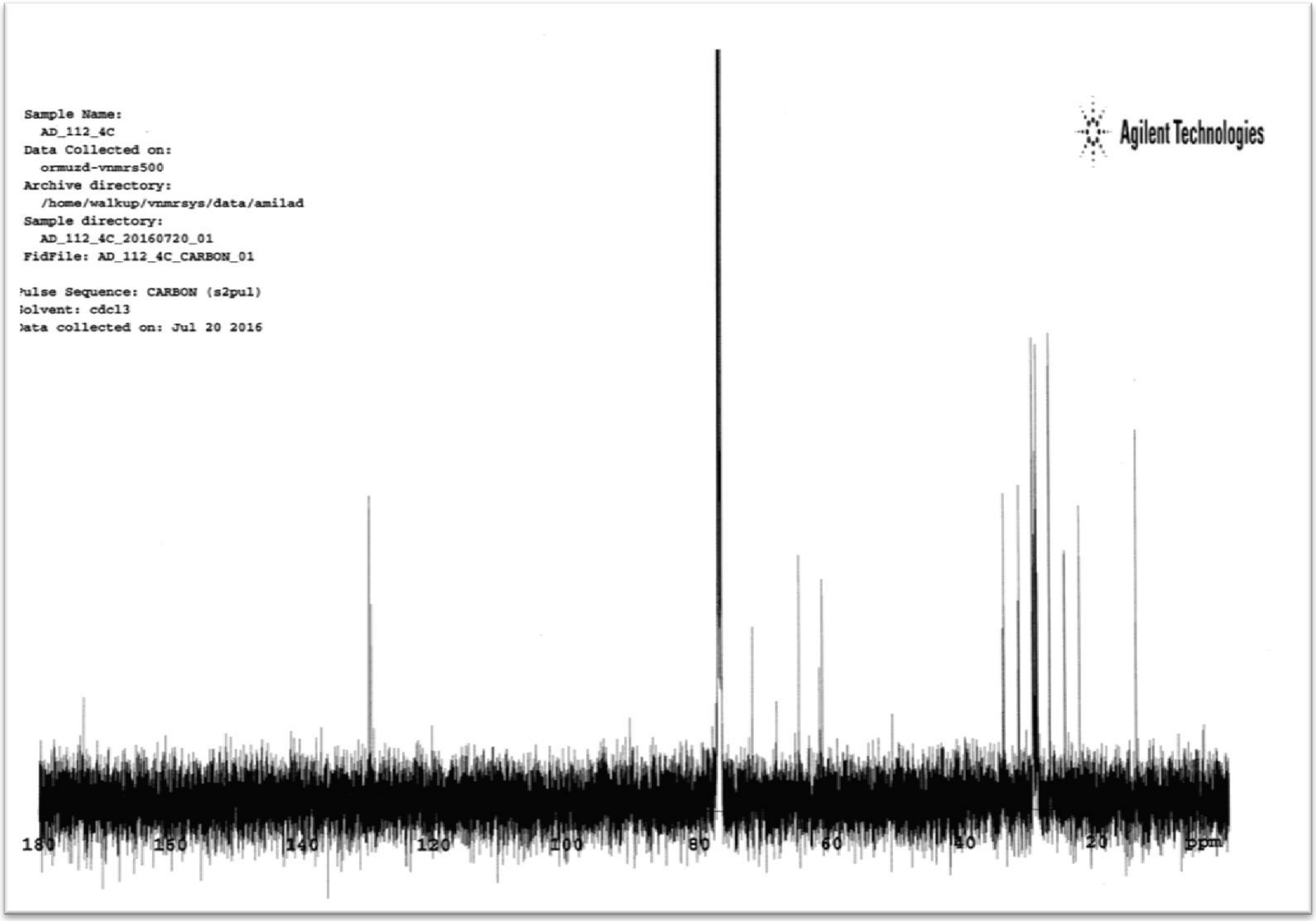
Sample Name:
AD_112_4C
Data Collected on:
ormuzd-vnmrs500
Archive directory:
/home/walkup/vnmrsys/data/amilad
Sample directory:
AD_112_4C_20160720_01
FidFile: AD_112_4C_CARBON_01
Pulse Sequence: CARBON (s2pul)
Solvent: cdcl3
Data collected on: Jul 20 2016
Agilent Technologies
180
160
140
120
100
80
60
40
20
ppm

Figure V

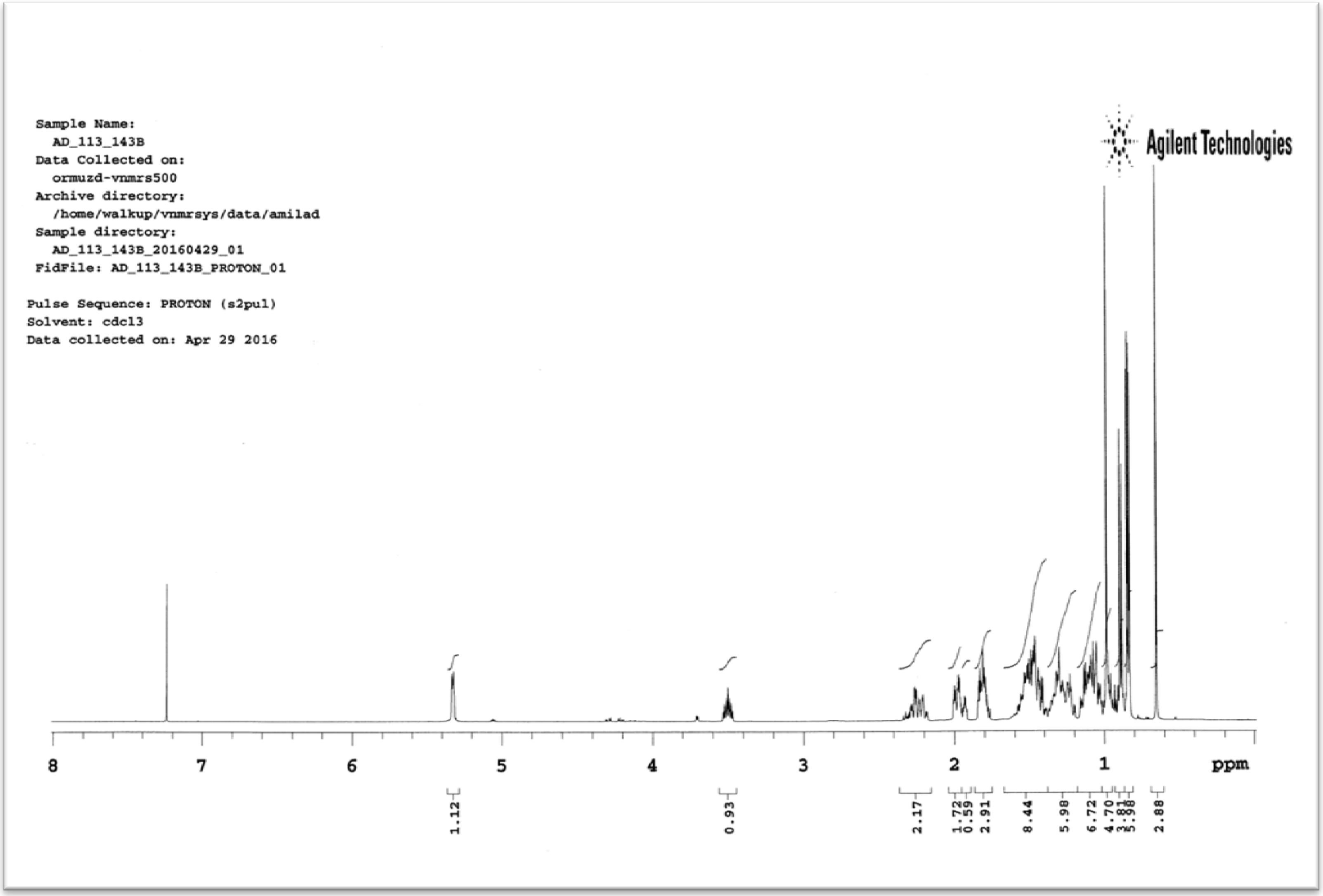
Sample Name:
AD_113_143B
Data Collected on:
ormuzd-vnmrs500
Archive directory:
/home/walkup/vnmrsys/data/amilad
Sample directory:
AD_113_143B_20160429_01
FidFile: AD_113_143B_PROTON_01
Pulse Sequence: PROTON (s2pul)
Solvent: cdcl3
Data collected on: Apr 29 2016
Agilent Technologies
8
7
6
5
4
3
2
1
ppm
1.12
0.93
2.17
1.72
0.59
2.91
8.44
5.98
6.72
4.70
3.81
5.98
2.88
