# Supplementary material for: Chemical Characterization of Lipophilic Constituents in the Skin of Migratory Adult Sea Lamprey from the Great Lakes Region
Source: PLoS One. 2016 Dec 19;11(12):e0168609. doi: 10.1371/journal.pone.0168609 (PMC5167404; doi:10.1371/journal.pone.0168609)
Supplement: S3 File — (PDF) [file pone.0168609.s003.pdf]

Chemical Characterization of Lipophilic Constituents in the Skin of Migratory Adult Sea Lamprey from the Great Lakes Region

Amila A. Dissanayake,[1] Muraleedharan G. Nair,[1,3*]

Bioactive Natural Products and Phytoceuticals Laboratory, Department of Horticulture, Michigan State University, East Lansing, Michigan 48824, USA

Supplemental data

For total lipid mixture
Figure A. ^{1}H NMR spectrum of total lipid mixture of sea lamprey in $CDCl_3$
Figure B. ^{13}C NMR spectrum of total lipid mixture of sea lamprey in $CDCl_3$
Figure C. GC profile of methyl esters of total lipid mixture

For fatty acid mixture **D**
Figure D. ^{1}H NMR spectrum of fatty acid mixture in $CDCl_3$
Figure E. GC profile of methyl esters of fatty acid mixture
Figure F. GCMS of the lauric acid, methyl ester
Figure G. GCMS of the myristic acid, methyl ester
Figure H. GCMS of the myristoleic acid, methyl ester
Figure I. GCMS of the palmitic acid, methyl ester
Figure J. GCMS of the stearic acid, methyl ester
Figure K. GCMS of the oleic acid, methyl ester
Figure L. GCMS of the arachidonic acid, methyl ester
Figure M. GCMS of the eicosapentaenoic acid, methyl ester
Figure N. GCMS of the docosahexaenoic acid, methyl ester

Figure A

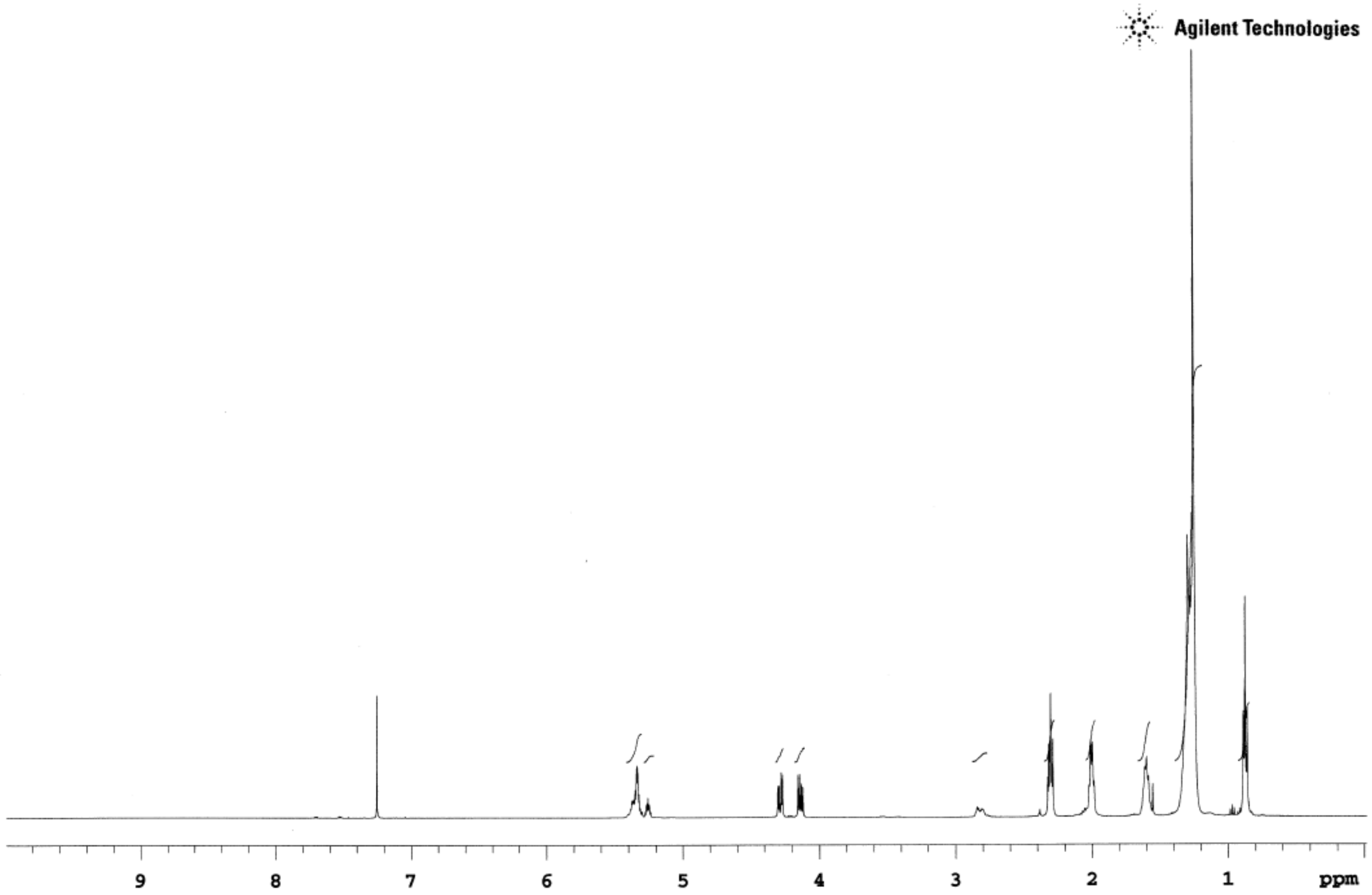

Figure B

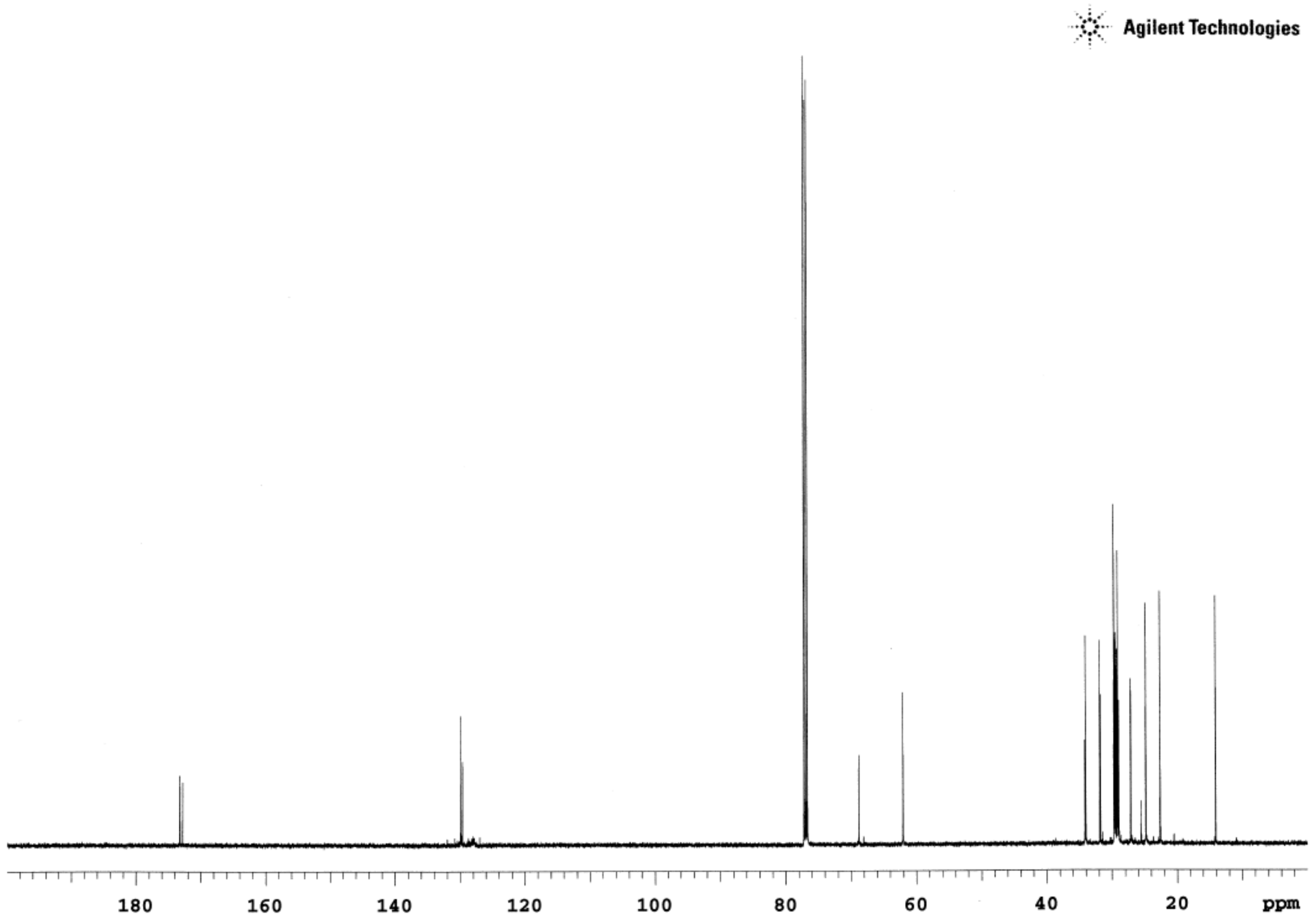

Figure C

```
File       :I:\Agilent A\Users\Nair\072814\a072814_06.D
Operator   :
Acquired   : 28 Jul 2014  17:40     using AcqMethod 30MDB5_GROB2_10SPL.M
Instrument :   EICI#1
Sample Name: 83f
Misc Info  :
Vial Number: 6
```

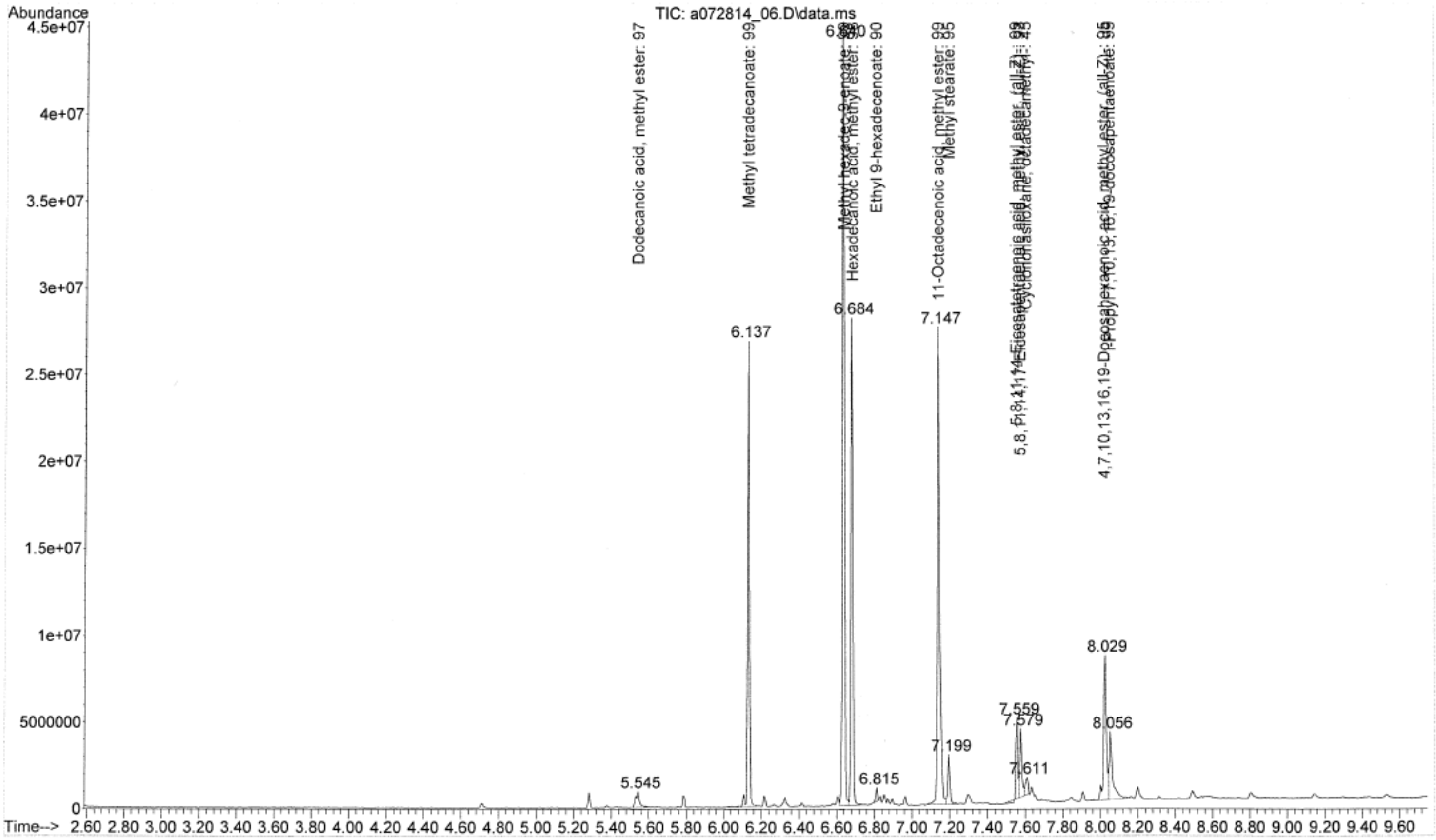

Figure D

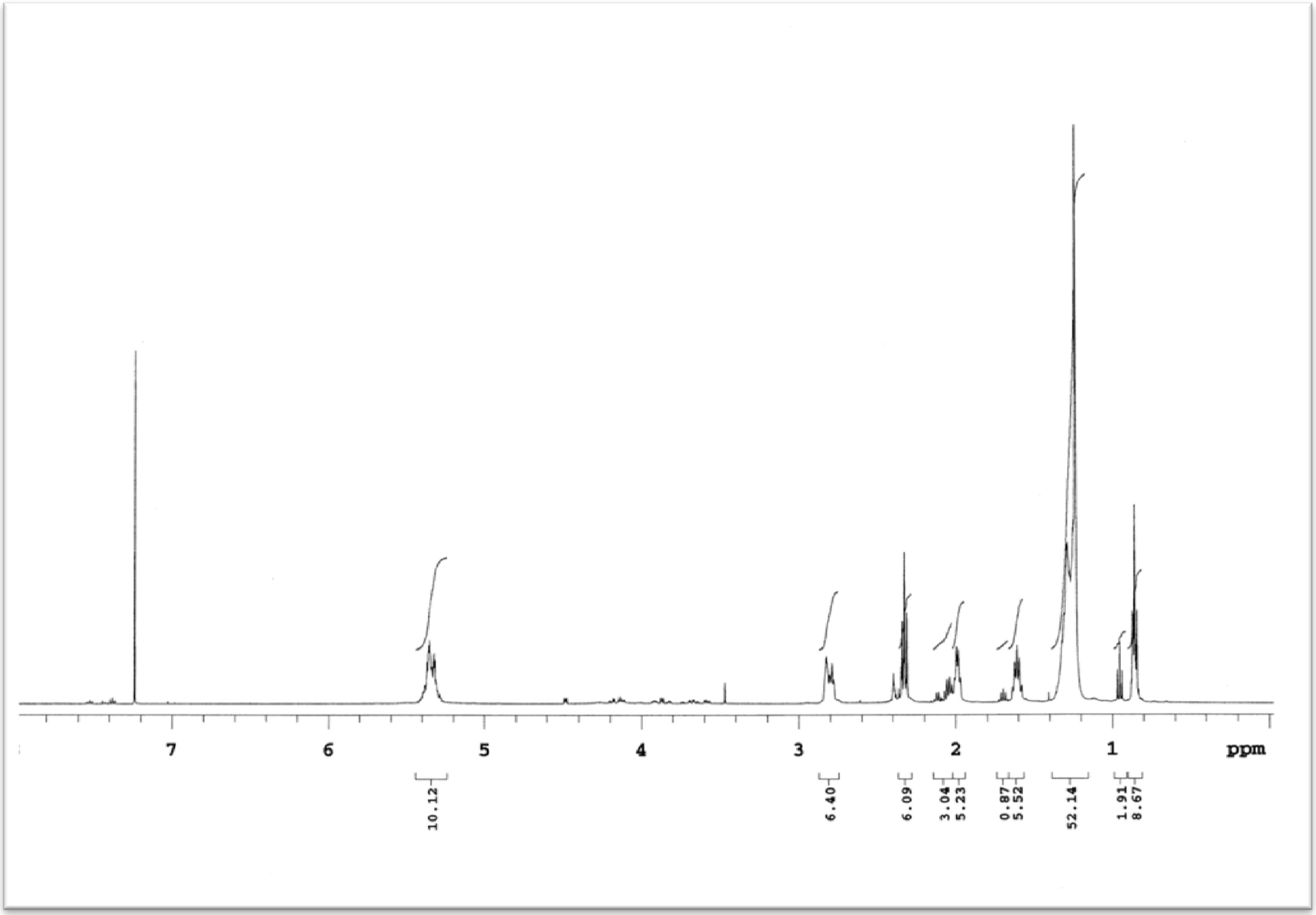

Figure E

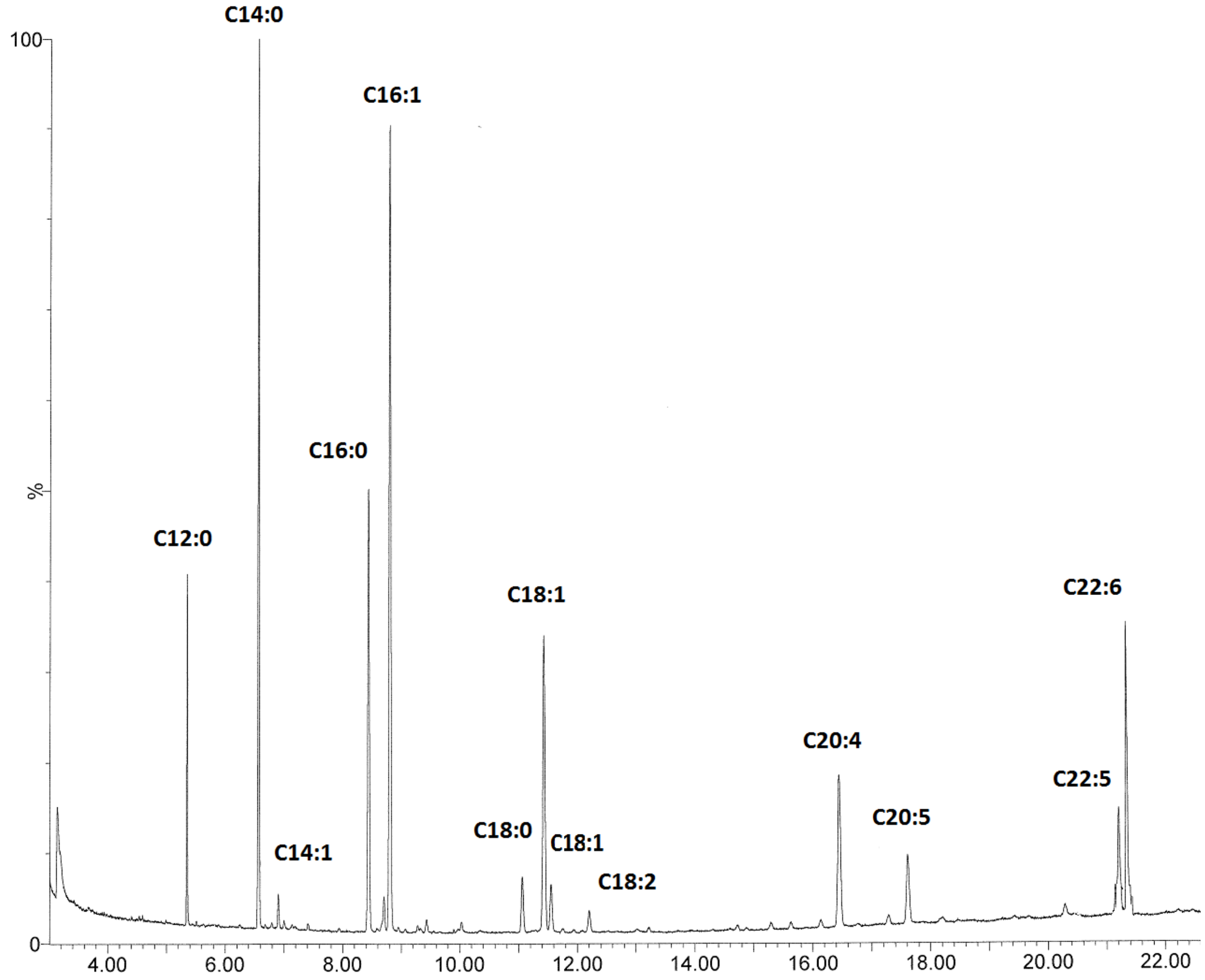

C14:0
C16:1
C16:0
C12:0
C18:1
C22:6
C20:4
C22:5
C20:5
C18:0
C18:1
C14:1
C18:2
100
%
0
4.00
6.00
8.00
10.00
12.00
14.00
16.00
18.00
20.00
22.00

Figure F

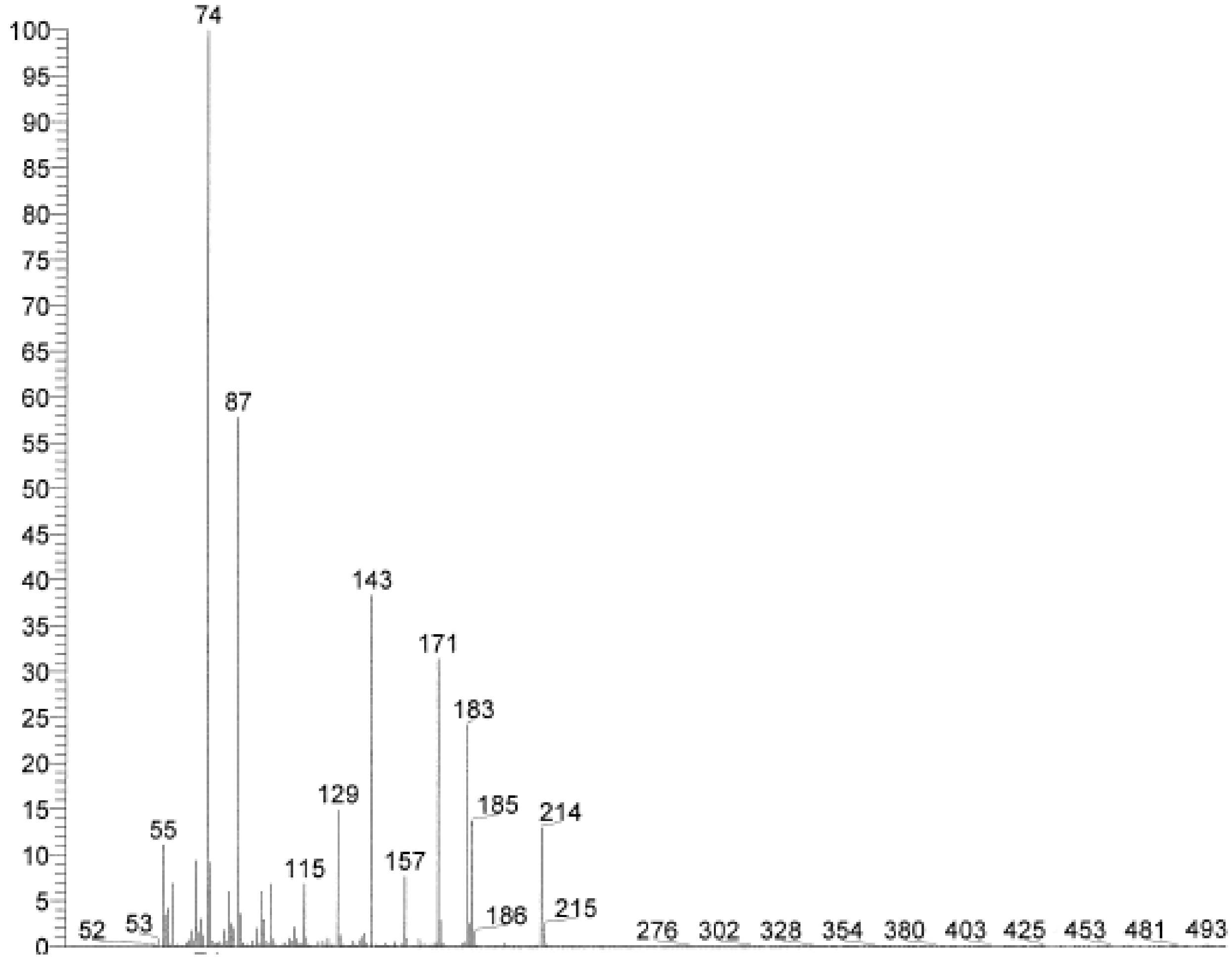
100
95
90
85
80
75
70
65
60
55
50
45
40
35
30
25
20
15
10
5
0
74
87
143
171
183
129
185
214
55
115
157
186
215
52
53
276
302
328
354
380
403
425
453
481
493

Figure G

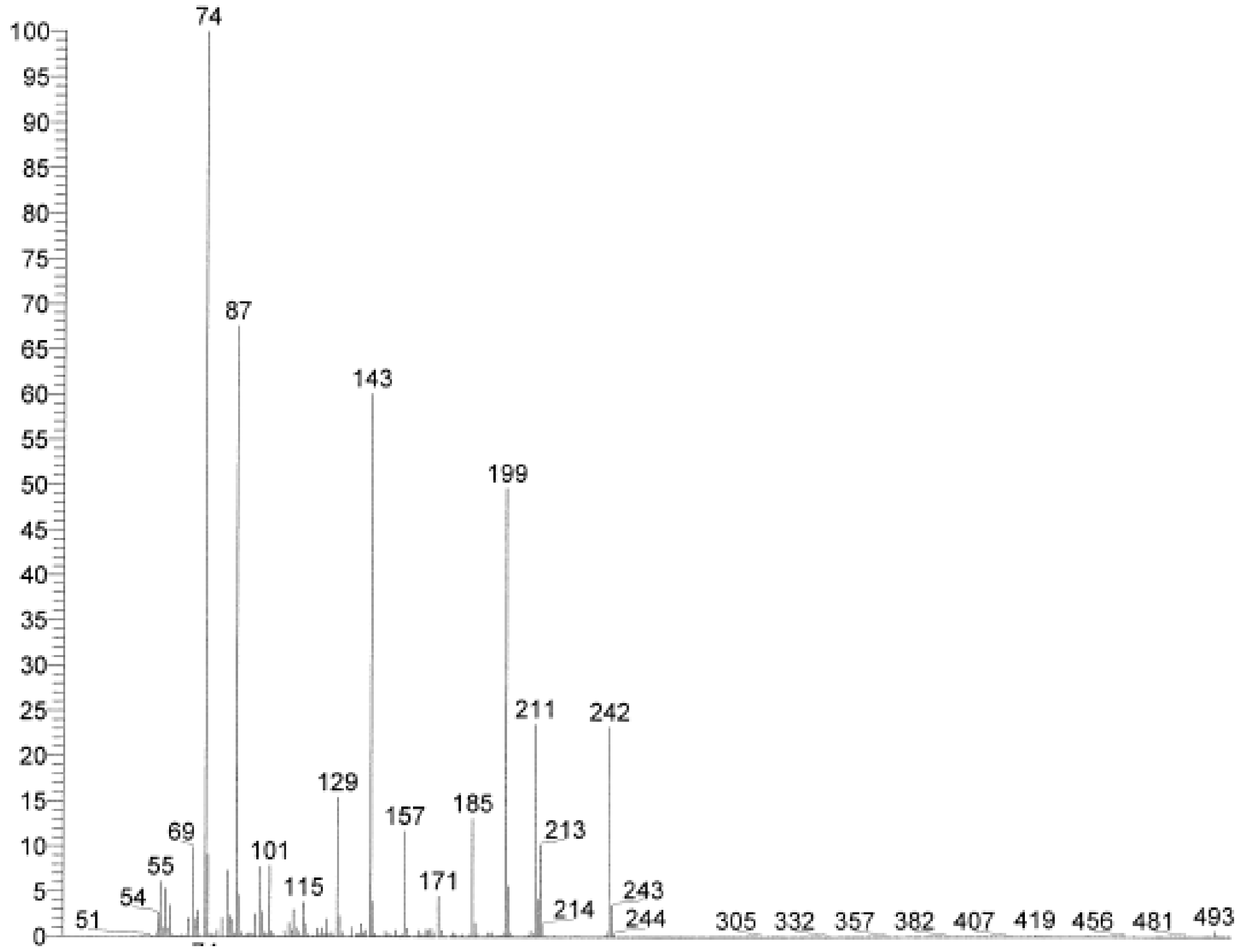

100
95
90
85
80
75
70
65
60
55
50
45
40
35
30
25
20
15
10
5
0
74
87
143
199
211
242
129
157
185
213
69
101
55
115
171
243
54
214
51
244
305
332
357
382
407
419
456
481
493

Figure H

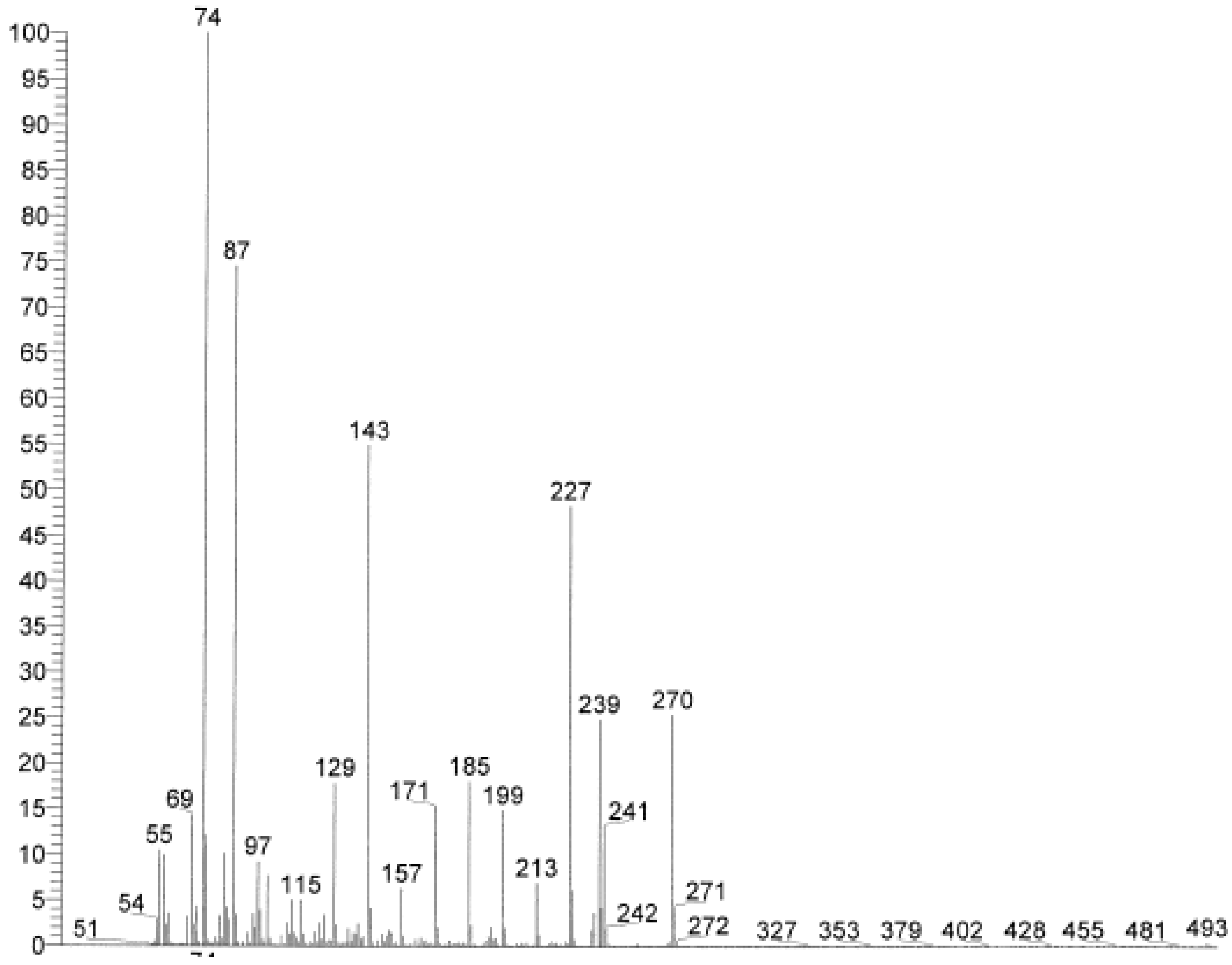

100
95
90
85
80
75
70
65
60
55
50
45
40
35
30
25
20
15
10
5
0
74
87
143
227
239
270
129
185
171
199
241
69
55
97
115
157
213
54
271
242
51
272
327
353
379
402
428
455
481
493

Figure I

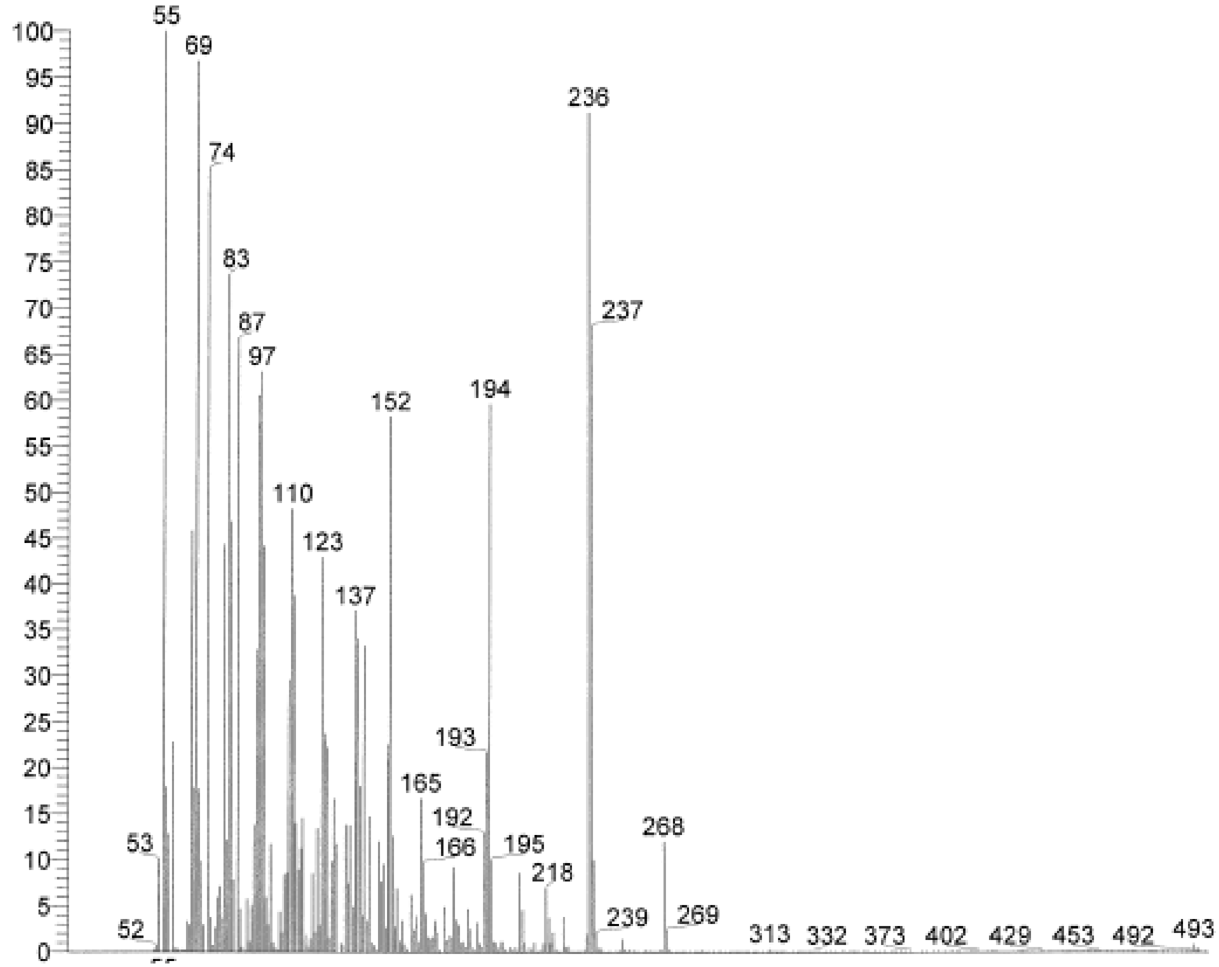

100
95
90
85
80
75
70
65
60
55
50
45
40
35
30
25
20
15
10
5
0
55
69
74
83
87
97
110
123
137
152
165
166
192
193
194
195
218
236
237
239
268
269
313
332
373
402
429
453
492
493
53
52

Figure J

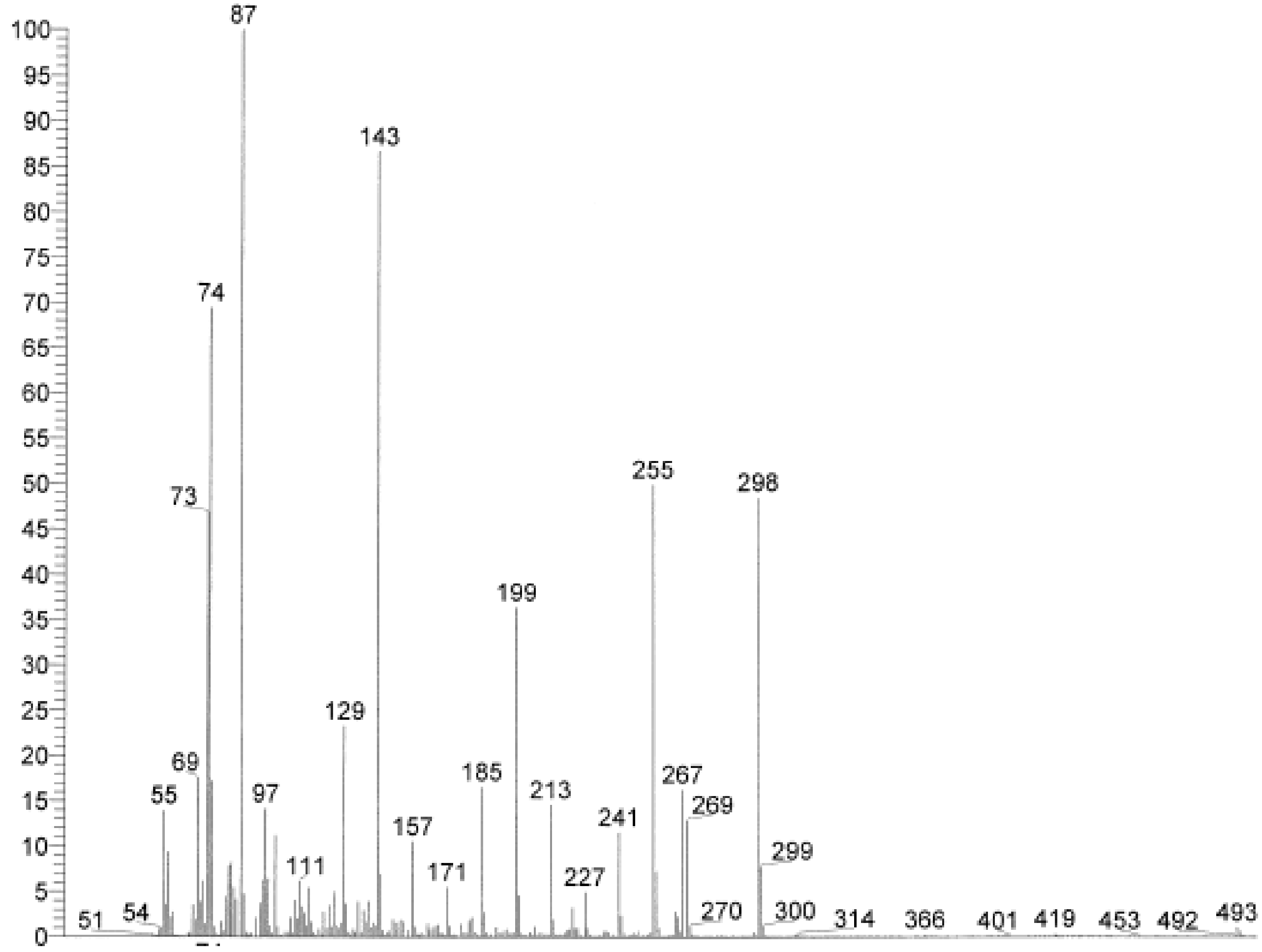

100
95
90
85
80
75
70
65
60
55
50
45
40
35
30
25
20
15
10
5
0
87
143
74
73
255
298
199
129
69
185
267
55
97
213
269
157
241
299
111
171
227
51
54
270
300
314
366
401
419
453
492
493

Figure K

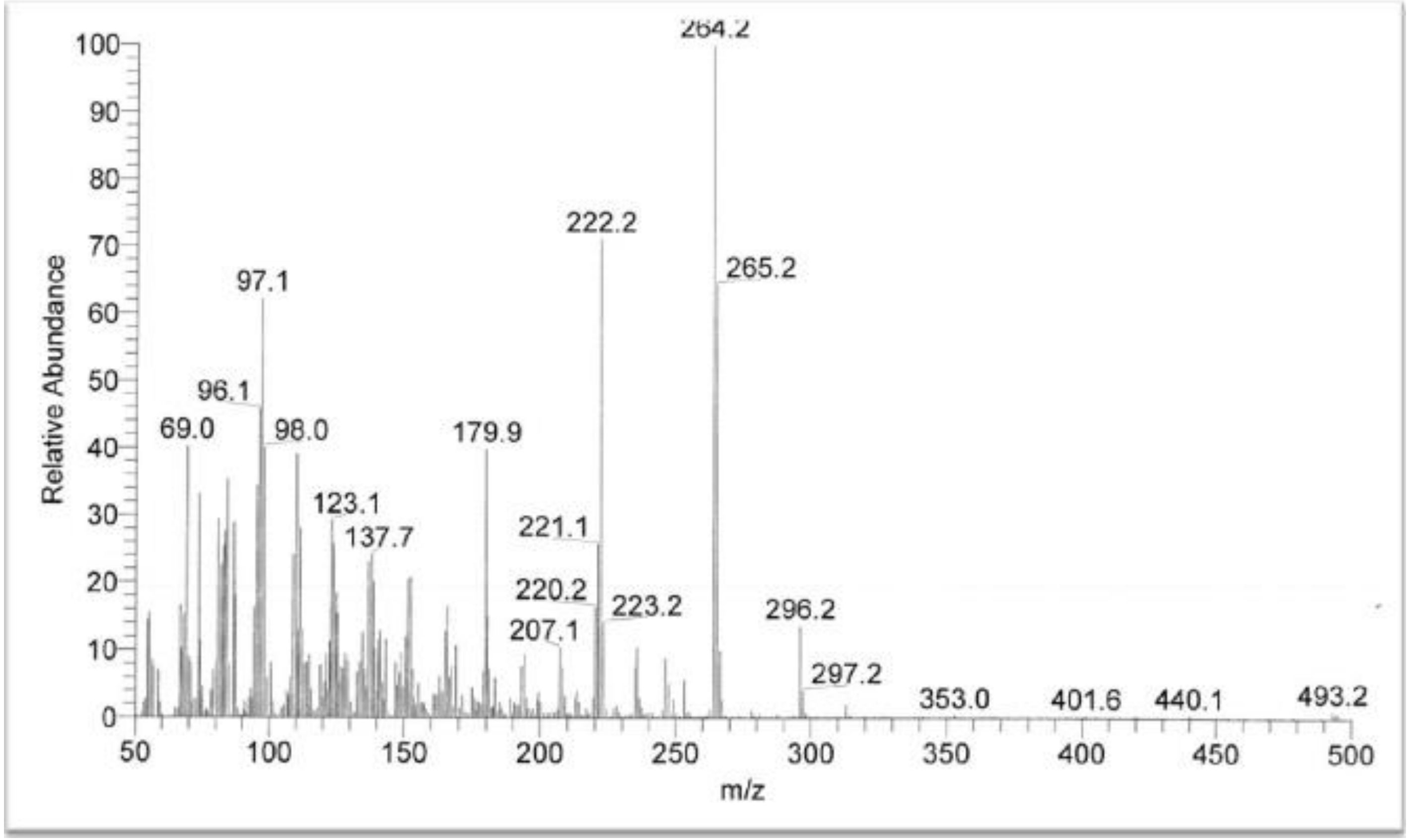

264.2
222.2
265.2
97.1
96.1
69.0
98.0
179.9
123.1
137.7
221.1
220.2
223.2
207.1
296.2
297.2
353.0
401.6
440.1
493.2
Relative Abundance
m/z

Figure L

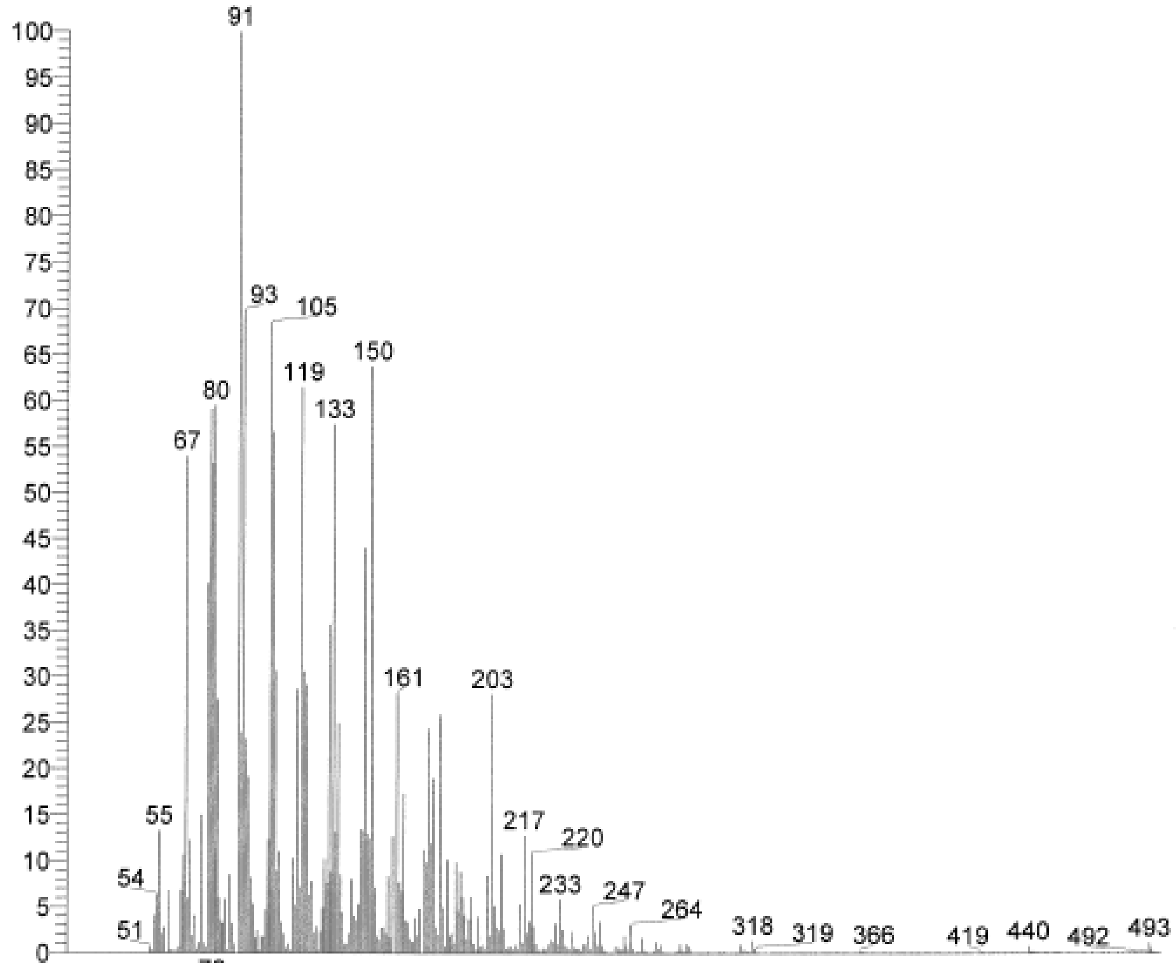

100
95
90
85
80
75
70
65
60
55
50
45
40
35
30
25
20
15
10
5
0
91
93
105
150
119
80
133
67
161
203
217
220
55
233
54
247
264
51
318
319
366
419
440
492
493

Figure M

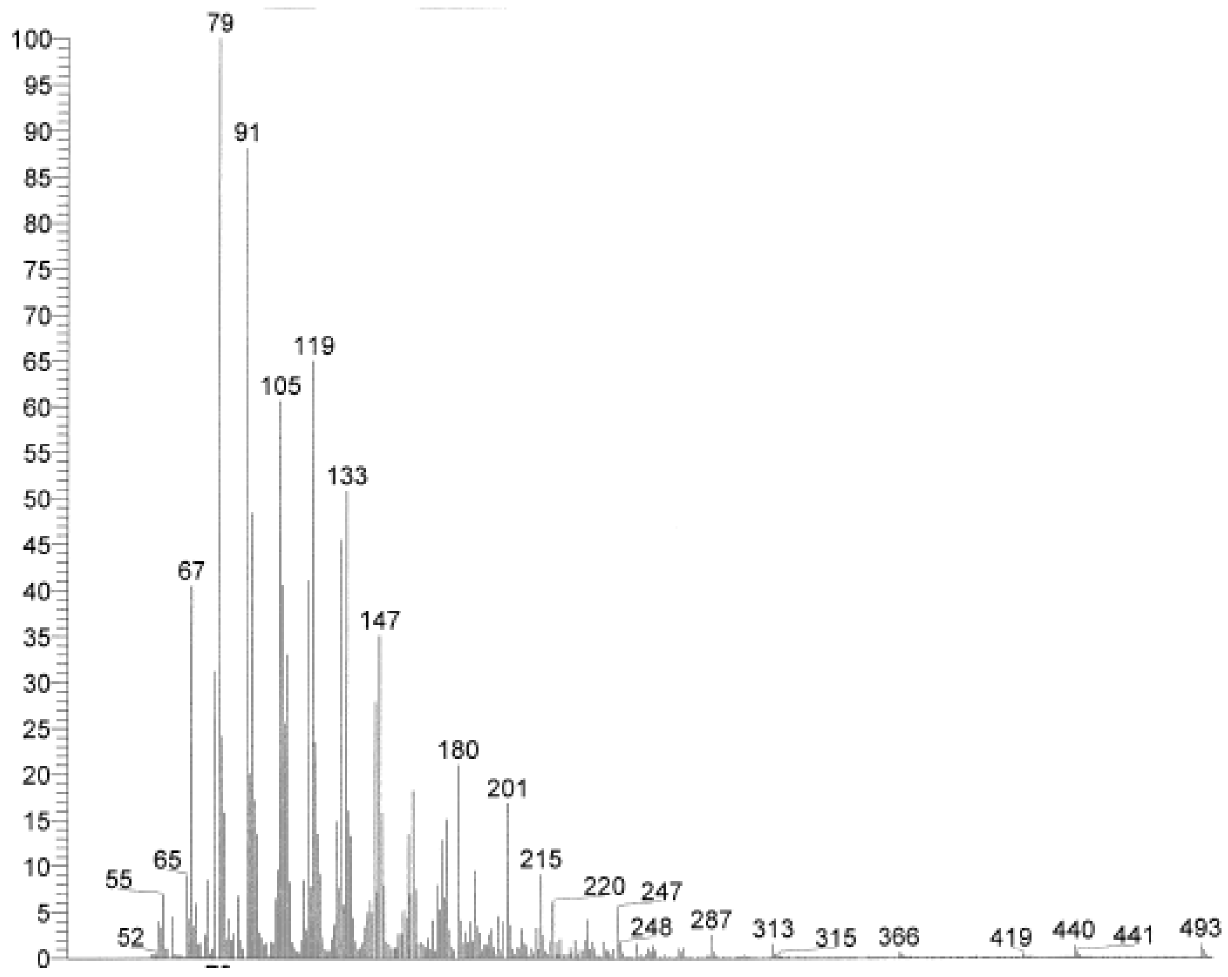

100
95
90
85
80
75
70
65
60
55
50
45
40
35
30
25
20
15
10
5
0
79
91
119
105
133
67
147
180
201
215
65
55
220
247
52
248
287
313
315
366
419
440
441
493

Figure N

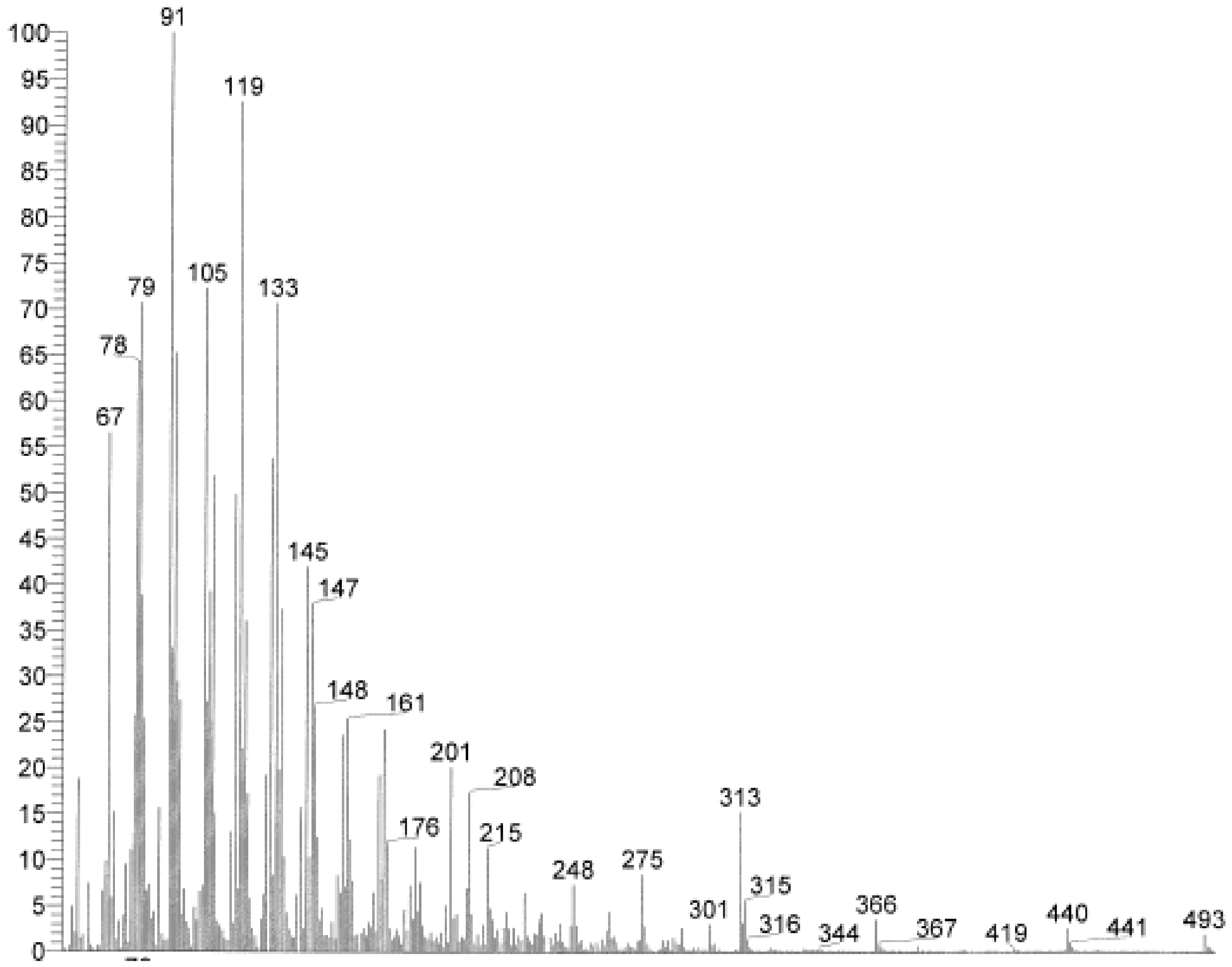

100
95
90
85
80
75
70
65
60
55
50
45
40
35
30
25
20
15
10
5
0
91
119
105
133
79
78
67
145
147
148
161
201
208
176
215
248
275
313
301
315
316
344
366
367
419
440
441
493
